# Supplementary material for: A fungal tolerance trait and selective inhibitors proffer HMG-CoA reductase as a herbicide mode-of-action
Source: Nat Commun. 2022 Sep 22;13:5563. doi: 10.1038/s41467-022-33185-0 (PMC9500038; doi:10.1038/s41467-022-33185-0)
Supplement: Supplementary file 1 — Supplementary Information [file 41467_2022_33185_MOESM1_ESM.pdf]

A fungal tolerance trait and selective inhibitors proffer HMG-CoA reductase as a herbicide mode-of-action

Haywood *et al.*

Supplementary Method 1. Compounds synthesis

Synthesis of 1-9 general experimental
All reagents and materials were purchased from commercial suppliers. Thin layer chromatography (TLC) was affected on Merck silica gel 60 F254 aluminium-backed plates and spots stained by heating with vanillin dip (6 g vanillin, 1 mL conc. H_2SO_4, 100 mL ethanol), unless stated otherwise. Flash column chromatography was performed on Merck silica gel using the specified solvents. NMR spectra were obtained on a Bruker Avance IIIHD 400, 500 or 600 spectrometers. The solvents used were $CDCl_3$ or DMSO-d_6 with $CHCl_3$ (^{1}H, δ 7.26 ppm), $CDCl_3$ (^{13}C, δ 77.16 ppm), $CD_3S(O)CD_2H$ (^{1}H, δ 2.50 ppm) or $(CD_3)_2SO$ (^{13}C, δ 39.52 ppm) used as an internal standard. Infrared spectra were obtained with neat samples on a PerkinElmer spectrum one FT-IR spectrometer fitted with a PerkinElmer Universal Attenuated Total Reflectance (ATR) sampling accessory. High resolution mass spectra (HR-MS) were obtained on a Waters LCT Premier XE TOF spectrometer, run in W-mode, using the ESI equipped ion source, in positive or negative mode. (**Supplementary Figure 7-10**).

Synthesis of 10-18 General Procedure A[1]
A solution of the methyl 3-oxo-alkanoate (9.50 mmol, 1.0 equiv), aniline (11.4 mmol, 1.2 equiv) and triethylamine (2.37 mmol, 0.25 equiv) in toluene (10 mL) were heated to reflux for 18 h. The solution was allowed to cool to r.t., and the resulting crystalline solid was filtered, washed with toluene (2 x 3 mL) and air dried to yield the compound of interest (**Supplementary Figure 7 and 8**).

Synthesis of β-Oxo-*N*-phenylcyclopropanepropanamide (10)
Prepared using General Procedure A (white solid, 1.45 g, 61%). ^{1}H NMR (600 MHz, $CDCl_3$): δ 9.37 (bs, 1H), 7.55-7.54 (m, 2H), 7.34-7.30 (m, 2H), 7.12-7.09 (m, 1H), 3.72 (s, 2H), 2.07-2.03 (m, 1H), 1.21-1.28 (m, 2H), 1.08-1.05 (m, 2H) (**Supplementary Figure 11**); ^{13}C NMR (151 MHz, $CDCl_3$): δ 207.9, 163.8, 137.7, 129.1, 124.6, 120.2, 49.1, 22.1, 12.6; HR-MS (ESI+): *m/z* calculated for $C_{12}H_{14}NO_2$ $[M+H]^+$: 204.1025, found: 204.1019 (**Supplementary Figure 12**).

Synthesis of 4,4-dimethyl-3-oxo-*N*-phenylpentanamide (11)
A solution of ethyl 4,4-dimethyl-3-oxo-pentanoate (2.07 mL, 11.6 mmol, 1.0 equiv), aniline (1.27 mL, 13.9 mmol, 1.2 equiv) and triethylamine (0.41 mL, 2.9 mmol, 0.25 equiv) in toluene (10 mL) were heated at 70 °C for 0.5 h, then to reflux for 4 h. The solution was allowed to cool to r.t., washed with 1M HCl (20 mL) and water (2 x 20 mL), dried over $MgSO_4$, filtered and concentrated, then purified by silica gel chromatography (10-15% EtOAc/hexanes) to yield the title compound as a pale yellow solid (1.61g, 63%). Spectral data matched those previously reported[1].

Synthesis of 3-Oxo-*N*-phenylhexanamide (12)
Prepared using General Procedure A (off-white solid, 2.32 g, 82%). ^{1}H NMR (400 MHz, $CDCl_3$): δ 9.15 (bs, 1H), 7.56-7.53 (m, 2H), 7.35-7.30 (m, 2H), 7.14-7.10 (m, 1H), 3.56 (s, 2H), 2.57 (t, J = 7.2 Hz, 2H), 1.67 (tt, J = 7.4, 7.2 Hz, 2H), 0.96 (t, J = 7.4 Hz, 3H) (**Supplementary Figure 13**); ^{13}C NMR (101 MHz, $CDCl_3$): δ 207.7, 163.7, 137.7, 129.1, 124.6, 120.2, 49.2, 46.1, 16.9, 13.6; FTIR (ATR): υ 3290, 1711, 1657, 1597, 1547 cm^{-1}; HR-MS (ESI+): *m/z* calculated for $C_{12}H_{16}NO_2$ $[M+H]^+$: 206.1181, found: 206.1183 (**Supplementary Figure 14**).

Synthesis of 3-Oxo-*N*-phenyl-5-(benzyloxy)pentanamide (13)
A solution of methyl 3-oxo-5-(benzyloxy)pentanoate[2] (2.98 g, 12.6 mmol, 1.0 equiv), aniline (2.30 mL, 25.2 mmol, 2.0 equiv) and DMAP (308 mg, 2.52 mmol, 0.20 equiv) in toluene (70 mL) were to reflux for 8 h. The solution was allowed to cool to r.t., then purified by silica gel chromatography (20-100% EtOAc/hexanes) to yield the title compound as a yellow oil (1.23 g, 31%). R_f 0.36 (40% EtOAc/hexanes); ^{1}H NMR (400 MHz, $CDCl_3$): δ 9.05 (bs, 1H), 7.53-7.51 (m, 2H), 7.35-7.27 (m, 7H), 9.13-7.09 (m, 1H), 4.52 (s, 2H), 3.78 (t, *J* = 5.9 Hz, 2H), 3.61 (s, 2H), 2.83 (t, *J* = 5.9 Hz, 2H) (**Supplementary Figure 15**); ^{13}C NMR (126 MHz, $CDCl_3$): δ 206.0, 163.6, 137.8, 137.6, 129.1, 128.6, 128.0, 127.9, 124.6, 120.3, 73.5, 64.9, 49.7, 44.2; FTIR (ATR): ʋ 3300, 1716, 1660, 1598, 1543 cm^{-1}; HR-MS (ESI+): *m/z* calculated for $C_{18}H_{19}NO_3Na$ $[M+Na]^+$: 320.1263, found: 320.1263 (**Supplementary Figure 16**).

Synthesis of 5-methyl-3-oxo-*N*-phenylhexanamide (14)
Prepared using General Procedure A (white solid, 843 mg, 61%). The filtrate was concentrated, redissolved in hot toluene (2 mL) and allowed to cool to r.t. The resulting solid was filtered, washed with toluene (2 x 1 mL) and air dried to yield further off-white crystalline solid (168 mg, 12%). Spectral data matched those previously reported[3].

Synthesis of 3-Oxo-*N*-phenylheptanamide (15)
A solution of methyl 3-oxo-heptanoate (1.50 g, 9.48 mmol, 1.0 equiv), aniline (1.04 mL, 11.4 mmol, 1.2 equiv) and triethylamine (0.33 mL, 2.4 mmol, 0.25 equiv) in toluene (10 mL) were heated to reflux for 18 h. The solution was allowed to cool to r.t., washed with 1M HCl (2 x 10 mL) and water (10 mL), dried over $MgSO_4$, filtered, concentrated to 5 mL and cooled in ice. The resultant solid was filtered, washed with cold toluene (2 x 3 mL) and air dried to yield the title compound as a cream solid (1.38 g, 66%). ^{1}H NMR (400 MHz, $CDCl_3$): δ 9.17 (bs, 1H), 7.55-7.53 (m, 2H), 7.34-7.29 (m, 2H), 7.13-7.09 (m, 1H), 3.56 (s, 2H), 2.58 (t, *J* = 7.4 Hz, 2H), 1.60 (tt, *J* = 7.5, 7.4 Hz, 2H), 1.34 (tt, *J* = 7.5, 7.3 Hz, 2H), 0.92 (t, *J* = 7.3 Hz, 3H) (**Supplementary Figure 17**); ^{13}C NMR (101 MHz, $CDCl_3$): δ 208.0, 163.6, 137.7, 129.1, 124.6, 120.2, 49.1, 44.0, 25.6, 22.2, 13.9; FTIR (ATR): ʋ 3254, 1713, 1657, 1598, 1548 cm^{-1}; HR-MS (ESI+): *m/z* calculated for $C_{13}H_{17}NO_2Na$ $[M+Na]^+$: 242.1157, found: 242.1153 (**Supplementary Figure 18**).

Synthesis of β-Oxo-*N*-phenylcyclobutanepropanamide (16)
A solution of ethyl 3-cyclobutyl-3-oxopropanoate (1.52 g, 8.93 mmol, 1.0 equiv), aniline (0.98 mL, 11 mmol, 1.2 equiv) and triethylamine (0.31 mL, 2.2 mmol, 0.25 equiv) in xylene (9 mL) were to reflux for 18 h. The solution was allowed to cool to r.t., washed with 1M HCl (10 mL) and water (2 x 10 mL), dried over $MgSO_4$, filtered and concentrated, then purified by silica gel chromatography (0-20% EtOAc/hexanes) to yield the title compound as a brown oil (1.32 g, 68%). R_f 0.24 (20% EtOAc/hexanes); ^{1}H NMR (400 MHz, $CDCl_3$): δ 9.27 (bs, 1H), 7.56-7.53 (m, 2H), 7.34-7.29 (m, 2H), 7.13-7.09 (m, 1H), 3.49 (s, 2H), 3.43-3.34 (m, 1H), 2.33-2.17 (m, 4H), 2.07-1.95 (m, 2H), 1.90-1.80 (m, 2H) (**Supplementary Figure 19**); ^{13}C NMR (101 MHz, $CDCl_3$): δ 208.5, 163.8, 137.7, 129.1, 124.6, 120.3, 46.5, 46.5, 24.3, 17.6; FTIR (ATR): ʋ 3289, 1699, 1654, 1599, 1532 cm^{-1}; HR-MS (ESI+): *m/z* calculated for $C_{13}H_{15}NO_2Na$ $[M+Na]^+$: 240.1000, found: 240.1000 (**Supplementary Figure 20**).

Synthesis of β-Oxo-N-phenylcyclopentanepropanamide (17)
A solution of ethyl 3-cyclopentyl-3-oxopropanoate (1.53 g, 8.31 mmol, 1.0 equiv), aniline (0.91 mL, 10 mmol, 1.2 equiv) and triethylamine (0.29 mL, 2.1 mmol, 0.25 equiv) in xylene (8 mL) were to reflux for 18 h. The solution was allowed to cool to r.t., washed with 1M HCl (10 mL) and water (2 x 10 mL), dried over $MgSO_4$, filtered and concentrated, then purified

by silica gel chromatography (0-20% EtOAc/hexanes) to yield the title compound as a brown oil (1.40 g, 73%). R_f 0.30 (20% EtOAc/hexanes); ^{1}H NMR (400 MHz, $CDCl_3$): δ 9.27 (bs, 1H), 7.56-7.53 (m, 2H), 7.34-7.29 (m, 2H), 7.13-7.08 (m, 1H), 3.60 (s, 2H), 3.03-2.95 (m, 1H), 1.92-1.74 (m, 4H), 1.72-1.58 (m, 4H) (**Supplementary Figure 21**); ^{13}C NMR (101 MHz, $CDCl_3$): δ 210.1, 163.9, 137.7, 129.1, 124.6, 120.2, 52.8, 48.2, 28.7, 26.1; FTIR (ATR): υ 3299, 1710, 1658, 1598, 1542 cm^{-1}; HR-MS (ESI+): *m/z* calculated for $C_{14}H_{17}NO_2Na$ $[M+Na]^+$: 254.1157, found: 254.1157 (**Supplementary Figure 22**).

Synthesis of β-Oxo-*N*-phenylcyclohexanepropanamide (18)
A solution of ethyl 3-cyclohexyl-3-oxopropanoate (1.20 g, 6.05 mmol, 1.0 equiv), aniline (0.66 mL, 7.3 mmol, 1.2 equiv) and triethylamine (0.21 mL, 1.5 mmol, 0.25 equiv) in toluene (6 mL) were heated to reflux for 24 h. The solution was allowed to cool to r.t., washed with 1M HCl (10 mL) and water (2 x 10 mL), dried over $MgSO_4$, filtered and concentrated, then purified by silica gel chromatography (0-20% EtOAc/hexanes) to yield the title compound as a cream solid (795 mg, 54%). R_f 0.37 (20% EtOAc/hexanes); ^{1}H NMR (400 MHz, $CDCl_3$): δ 9.22 (bs, 1H), 7.55-7.53 (m, 2H), 7.34-7.29 (m, 2H), 7.13-7.09 (m, 1H), 3.59 (s, 2H), 2.50-2.43 (m, 1H), 1.93-1.89 (m, 2H), 1.83-1.78 (m, 2H), 1.72-1.67 (m, 2H), 1.42-1.16 (m, 4H) (**Supplementary Figure 23**); ^{13}C NMR (101 MHz, $CDCl_3$): δ 211.0, 163.8, 137.7, 129.1, 124.6, 120.2, 52.1, 47.3, 28.1, 25.8, 25.5; FTIR (ATR): υ 3257, 1711, 1659, 1600, 1557 cm^{-1}; HR-MS (ESI+): *m/z* calculated for $C_{15}H_{19}NO_2Na$ $[M+Na]^+$: 268.1313, found: 268.1312 (**Supplementary Figure 24**).

Synthesis of 19-27 General Procedure B[4]
The 3-oxo-*N*-phenylalkanamide **10-18** (5.31 mmol, 1.0 equiv) and 2-bromo-1-(3-fluorophenyl)-2-phenylethanone[5] (5.31 mmol, 1.0 equiv) and potassium carbonate (7.97 mmol, 1.5 equiv) were stirred in acetone (8 mL) at r.t. while protected from light for 18 h. The mixture was then filtered, and the filtrate was purified by silica gel chromatography (10-20% EtOAc/hexanes) to yield the intermediate 4-fluoro-α-(1-oxoalkyl)-γ-oxo-*N*,β-diphenylbenzene butyramide as a mixture of diastereomers, which were then used in Procedure C. (**Supplementary Figure 7**)

Synthesis of 19-27 General Procedure C[6]
The 4-fluoro-α-(1-oxoalkyl)-γ-oxo-*N*,β-diphenylbenzene butyramide from General Procedure B (2.32 mmol, 1.0 equiv), (4*R*,6*R*)-*tert*-butyl-6-(2-aminoethyl)-2,2-dimethyl-1,3-dioxane-4-acetate (2.39 mmol, 1.03 equiv) and pivalic acid (1.55 mmol, 0.67 equiv) in 4:1:1 heptane/toluene/THF (15 mL) was heated to reflux for 18 h, then cooled to r.t., washed with 0.5 M NaOH (15 mL), 0.5 M HCl (15 mL) and water (5 mL), then dried over $MgSO_4$, filtered and concentrated. The residue was purified by silica gel chromatography (5-40% EtOAc/hexanes) to yield the compound of interest. (**Supplementary Figure 7 and 9**)

Synthesis of 1,1-dimethylethyl (4*R*,6*R*)-6-[2-[5-cyclopropyl-2-(4-fluorophenyl)-3-phenyl-4-[(phenylamino)carbonyl]-1*H*-pyrrol-1-yl]ethyl]-2,2-dimethyl-1,3-dioxane-4-acetate (19)
Prepared using General Procedure B (pale yellow resin, 706 mg, 24%; R_f 0.19 (20% EtOAc/hexanes)), followed by General Procedure C (pale yellow resin, 800 mg, 75%). R_f 0.16 (20% EtOAc/hexanes); ^{1}H NMR (500 MHz, $CDCl_3$): δ 7.22-7.13 (m, 11H), 7.02-6.97 (m, 3H), 6.89 (s, 1H), 4.25-4.19 (m, 1H), 4.16-4.10 (m, 2H), 3.66-3.61 (m, 1H), 2.36 (dd, J = 15.2, 7.0 Hz, 1H), 2.22 (dd, J = 15.2, 6.2 Hz, 1H), 1.95-1.89 (m, 1H), 1.66-1.54 (m, 2H), 1.43 (s, 9H), 1.36 (s, 3H), 1.32-1.29 (m, 1H), 1.28 (s, 3H), 1.13-1.09 (m, 2H), 1.02 (ddd, J = 11.9, 11.9, 11.9 Hz, 1H), 0.79-0.76 (m, 2H) (**Supplementary Figure 25**); ^{13}C NMR (126 MHz, $CDCl_3$): δ 170.3, 163.9, 162.4 (d, J = 248 Hz), 138.6, 136.7, 134.5, 133.0 (d, J = 8 Hz),

130.6, 129.3, 128.9, 128.4, 128.2 (d, *J* = 3 Hz), 126.7, 123.7, 121.2, 119.6, 118.0, 115.6 (d, *J* = 22 Hz), 98.8, 80.8, 66.3, 66.0, 42.6, 40.5, 37.3, 36.1, 30.1, 28.2, 19.7, 7.8, 7.7, 7.0; FTIR (ATR): ʋ 1727, 1667, 1595, 1509 cm^{-1}; HR-MS (ESI+): *m/z* calculated for $C_{40}H_{45}N_2O_5FNa$ $[M+Na]^+$: 675.3210, found: 675.3208 (**Supplementary Figure 26**).

Synthesis of 1,1-dimethylethyl (4*R*,6*R*)-6-[2-[2-(4-fluorophenyl)-5-(1,1-dimethylethyl)-3-phenyl-4-[(phenylamino)carbonyl]-1*H*-pyrrol-1-yl]ethyl]-2,2-dimethyl-1,3-dioxane-4-acetate (20)

Prepared using General Procedure B (white solid, 594 mg, 19%; R_f 0.30 (20% EtOAc/hexanes)), followed by General Procedure C (light yellow resin, 102 mg, 11%). R_f 0.28 (20% EtOAc/hexanes); ^1H NMR (600 MHz, $CDCl_3$): δ 7.23-7.21 (m, 2H), 7.18-7.16 (m, 4H), 7.08-7.06 (m, 4H), 7.05-6.99 (m, 5H), 4.38-4.33 (m, 1H), 4.12-4.08 (m, 1H), 4.06-4.01 (m, 1H), 3.55-3.51 (m, 1H), 2.34 (dd, *J* = 15.3, 7.0 Hz, 1H), 2.20 (dd, *J* = 15.3, 6.2 Hz, 1H), 1.58 (s, 9H), 1.53-1.44 (m, 2H), 1.42 (s, 9H), 1.34 (s, 3H), 1.27 (s, 3H), 1.19 (ddd, *J* = 12.7, 2.4, 2.4 Hz, 1H), 0.93 (ddd, *J* = 11.9, 11.9, 11.9 Hz, 1H) (**Supplementary Figure 27**); ^{13}C NMR (151 MHz, $CDCl_3$): δ 170.3, 168.0, 162.3 (d, *J* = 248 Hz), 138.1, 134.5, 133.2 (d, *J* = 8 Hz), 130.4, 129.6, 128.9, 128.8 (d, *J* = 3 Hz), 128.1, 125.9, 124.3, 122.0, 120.5, 118.8, 115.6 (d, *J* = 22 Hz), 98.7, 80.8, 66.6, 66.0, 42.6, 37.4, 35.9, 34.2, 31.5, 30.0, 28.2, 19.8; FTIR (ATR): ʋ 3296, 1741, 1650, 1533 cm^{-1}; HR-MS (ESI+): *m/z* calculated for $C_{41}H_{49}N_2O_5FNa$ $[M+Na]^+$: 691.3523, found: 691.3524 (**Supplementary Figure 28**).

Synthesis of 1,1-dimethylethyl (4*R*,6*R*)-6-[2-[2-(4-fluorophenyl)-3-phenyl-4-[(phenylamino)carbonyl-5-propyl]-1*H*-pyrrol-1-yl]ethyl]-2,2-dimethyl-1,3-dioxane-4-acetate (21)

Prepared using General Procedure B, followed by trituration with DCM/hexane (1:5, 4 mL) (white solid, 1.13 g, 56%; R_f 0.30 (20% EtOAc/hexanes)), followed by General Procedure C (light yellow resin, 1.21 g, 71%). R_f 0.44 (20% EtOAc/hexanes); ^1H NMR (400 MHz, $CDCl_3$): δ 7.28-7.25 (m, 3H), 7.24-7.21 (m, 2H), 7.19-7.13 (m, 4H), 7.06-7.03 (m, 2H), 7.00-6.93 (m, 4H), 4.19-4.13 (m, 1H), 4.06-3.98 (m, 1H), 3.90-3.83 (m, 1H), 3.68-3.61 (m, 1H), 3.20 (dt, *J* = 15.8 7.1 Hz, 1H), 3.04 (dt, *J* = 15.8 7.1 Hz, 1H), 2.38 (dd, *J* = 15.3, 7.0 Hz, 1H), 2.23 (dd, *J* = 15.3, 6.2 Hz, 1H), 1.79-1.70 (m, 2H), 1.65-1.54 (m, 2H), 1.43 (s, 9H), 1.38 (s, 3H), 1.36-1.32 (m, 1H), 1.32 (s, 3H), 1.08-1.00 (m, 1H), 1.07 (t, *J* = 7.3 Hz, 3H) (**Supplementary Figure 29**); ^{13}C NMR (101 MHz, $CDCl_3$): δ 170.3, 163.9, 162.4 (d, *J* = 248 Hz), 139.7, 138.7, 134.9, 133.0 (d, *J* = 8 Hz), 131.3, 129.4, 128.8, 128.7, 128.2 (d, *J* = 3 Hz), 127.3, 123.3, 121.6, 119.4, 115.5 (d, *J* = 21 Hz), 114.1, 98.9, 80.8, 66.0, 42.6, 40.2, 37.8, 36.2, 30.1, 28.2, 27.5, 23.9, 19.8, 14.5; FTIR (ATR): ʋ 3401, 1727, 1661, 1595, 1529 cm^{-1}; HR-MS (ESI+): *m/z* calculated for $C_{40}H_{47}N_2O_5FNa$ $[M+Na]^+$: 677.3367, found: 677.3369 (**Supplementary Figure 30**).

Synthesis of 1,1-dimethylethyl (4*R*,6*R*)-6-[5-(2-benzyloxyethyl)-2-[2-(4-fluorophenyl)-3-phenyl-4-[(phenylamino)carbonyl]-1*H*-pyrrol-1-yl]ethyl]-2,2-dimethyl-1,3-dioxane-4-acetate (22)

Prepared using General Procedure B with 2-iodo-1-(3-fluorophenyl)-2-phenylethanone[7] (yellow resin, 936 mg, 46%; R_f 0.22 (20% EtOAc/hexanes)), followed by General Procedure C (light yellow resin, 204 mg, 15%). *Note* - unstable in $CHCl_3$. ^1H NMR (500 MHz, CD_3CN): δ 8.18 (s, 1H), 7.33-7.18 (m, 16H), 2.09-2.05 (m, 2H), 7.00-6.97 (m, 1H), 4.62-4.57 (m, 2H), 4.16-4.11 (m, 1H), 3.99-3.93 (m, 1H), 3.89-3.82 (m, 3H), 3.67-3.62 (m, 1H), 3.38-3.32 (m, 1H), 3.30-3.24 (m, 1H), 2.23 (dd, *J* = 15.0, 4.9 Hz, 1H), 2.18-2.13 (m, 1H), 1.53-1.40 (m, 11H), 1.32 (s, 3H), 1.27-1.23 (m, 1H), 1.20 (m, 3H), 0.92-0.85 (ddd, *J* = 11.9, 11.9, 11.9 Hz, 1H) (**Supplementary Figure 31**); ^{13}C NMR (126 MHz, CD_3CN): δ 170.9, 164.7,

163.3 (d, *J* = 246 Hz), 140.1, 139.5, 136.3, 134.5 (d, *J* = 8 Hz), 134.0, 131.7, 131.0, 129.6, 129.5 (d, *J* = 3 Hz), 129.4, 129.0, 128.7, 128.6, 127.5, 124.1, 123.2, 120.1, 117.9, 116.1 (d. *J* = 22 Hz), 99.4, 81.0, 73.6, 70.7, 67.0, 43.4, 41.2, 38.1, 36.6, 30.4, 28.3, 26.8, 20.0. FTIR (ATR): ʋ 3401, 1727, 1660, 1530 cm^{-1}; HR-MS (ESI+): *m/z* calculated for $C_{46}H_{52}N_2O_6F$ $[M+H]^+$: 747.3809, found: 747.3804 (**Supplementary Figure 32**).

Synthesis of 1,1-dimethylethyl (4*R*,6*R*)-6-[2-[2-(4-fluorophenyl)-5-(2-methylpropyl)-3-phenyl-4-[(phenylamino)carbonyl]-1*H*-pyrrol-1-yl]ethyl]-2,2-dimethyl-1,3-dioxane-4-acetate (23)

Prepared using General Procedure B (off-white solid, 977 mg, 49%; R_f 0.34 (20% EtOAc/hexanes)), followed by General Procedure C (off-white resin, 793 mg, 53%). R_f 0.40 (20% EtOAc/hexanes); ^{1}H NMR (500 MHz, $CDCl_3$): δ 7.29-7.23 (m, 3H), 7.22-7.20 (m, 2H), 7.18-7.13 (m, 4H), 7.04-7.02 (m, 2H), 7.00-6.94 (m, 3H), 6.91 (s, 1H), 4.14-4.12 (m, 1H), 4.06-4.00 (m, 1H), 3.94-3.88 (m, 1H), 3.62-3.57 (m, 1H), 3.19 (dd, *J* = 14.2, 7.6 Hz, 1H), 3.01 (dd, *J* = 14.2, 7.1 Hz, 1H), 2.37 (dd, *J* = 15.3, 7.0 Hz, 1H), 2.22 (dd, *J* = 15.3, 6.2 Hz, 1H), 2.05-1.97 (m, 1H), 1.56-1.44 (m, 2H), 1.43 (s, 9H), 1.38 (s, 3H), 1.33 (s, 3H), 1.30 (ddd, *J* = 12.8, 2.4, 2.4 Hz, 1H), 1.05-0.98 (m, 7H) (**Supplementary Figure 33**); ^{13}C NMR (126 MHz, $CDCl_3$): δ 170.3, 164.2, 162.3 (d, *J* = 248 Hz), 138.6, 138.6, 134.9, 133.0 (d, *J* = 8 Hz), 131.2, 129.5, 128.8, 128.7, 128.3 (d, *J* = 3 Hz), 127.2, 123.3, 121.6, 119.5, 115.5 (d, *J* = 22 Hz), 115.1, 98.9, 80.8, 66.0, 65.8, 42.6, 40.3, 37.4, 36.2, 30.1, 30.1, 28.2, 22.7, 22.5, 19.8; FTIR (ATR): ʋ 3405, 1727, 1663, 1595, 1528 cm^{-1}; HR-MS (ESI+): *m/z* calculated for $C_{41}H_{49}N_2O_5FNa$ $[M+Na]^+$: 691.3523, found: 691.3528 (**Supplementary Figure 34**).

Synthesis of 1,1-dimethylethyl (4*R*,6*R*)-6-[2-[5-butyl-2-(4-fluorophenyl)-3-phenyl-4-[(phenylamino)carbonyl]-1*H*-pyrrol-1-yl]ethyl]-2,2-dimethyl-1,3-dioxane-4-acetate (24)

Prepared using General Procedure B (off-white resin, 1.29 g, 56%; R_f 0.35 (20% EtOAc/hexanes)), followed by General Procedure C (light yellow resin, 914 mg, 59%). R_f 0.50 (20% EtOAc/hexanes); ^{1}H NMR (500 MHz, $CDCl_3$): δ 7.30-7.25 (m, 3H), 7.24-7.21 (m, 2H), 7.18-7.13 (m, 4H), 7.06-7.04 (m, 2H), 7.00-6.94 (m, 4H), 4.19-4.13 (m, 1H), 4.02 (ddd, *J* = 14.4, 9.7, 4.7 Hz, 1H), 3.87 (ddd, *J* = 14.4, 9.4, 7.0 Hz, 1H), 3.67-3.62 (m, 1H), 3.23 (ddd, *J* = 14.1, 9.0, 7.0 Hz, 1H), 3.07 (ddd, *J* = 14.1, 8.9, 6.9 Hz, 1H), 2.38 (dd, *J* = 15.3, 7.0 Hz, 1H), 2.23 (dd, *J* = 15.3, 6.2 Hz, 1H), 1.72-1.46 (m, 6H), 1.43 (s, 9H), 1.38 (s, 3H), 1.35-1.32 (m, 1H), 1.32 (s, 3H), 1.05 (ddd, *J* = 11.9, 11.9, 11.9 Hz, 1H), 0.99 (t, *J* = 7.4 Hz, 3H) (**Supplementary Figure 35**); ^{13}C NMR (126 MHz, $CDCl_3$): δ 170.3, 163.9, 162.3 (d, *J* = 248 Hz), 139.9, 138.7, 134.9, 133.0 (d, *J* = 8 Hz), 131.3, 129.4, 128.8, 128.7, 128.2 (d, *J* = 3 Hz), 127.3, 123.3, 121.6, 119.4, 115.5 (d, *J* = 21 Hz), 114.0, 98.9, 80.8, 66.0, 42.6, 40.2, 37.7, 36.2, 32.8, 30.1, 28.2, 25.2, 23.1, 19.8, 14.2; FTIR (ATR): ʋ 3404, 1727, 1662, 1594, 1529 cm^{-1}; HR-MS (ESI+): *m/z* calculated for $C_{41}H_{49}N_2O_5FNa$ $[M+Na]^+$: 691.3523, found: 691.3521 (**Supplementary Figure 36**).

Synthesis of 1,1-dimethylethyl (4*R*,6*R*)-6-[2-[5-cyclobutyl-2-(4-fluorophenyl)-3-phenyl-4-[(phenylamino)carbonyl]-1*H*-pyrrol-1-yl]ethyl]-2,2-dimethyl-1,3-dioxane-4-acetate (25)

Prepared using General Procedure B (cream solid, 1.08 g, 55%; R_f 0.28 (20% EtOAc/hexanes)), followed by General Procedure C (light yellow resin, 1.30 g, 88%). R_f 0.32 (20% EtOAc/hexanes); ^{1}H NMR (600 MHz, $CDCl_3$): δ 7.22-7.11 (m, 11H), 7.02-6.98 (m, 3H), 6.88 (bs, 1H), 4.16-4.11 (m, 1H), 4.04 (ddd, *J* = 14.5, 9.9, 4.8 Hz, 1H), 3.97-3.91 (m, 1H), 3.85 (ddd, *J* = 14.5, 9.8, 6.3 Hz, 1H), 3.64-3.60 (m, 1H), 2.64-2.55 (m, 2H), 2.47-2.42 (m, 2H), 2.37 (dd, *J* = 15.3, 7.0 Hz, 1H), 2.22 (dd, *J* = 15.3, 6.2 Hz, 1H), 2.06-1.98 (m, 1H), 1.92-1.87 (m, 1H), 1.61-1.58 (m, 2H), 1.43 (s, 9H), 1.36 (s, 3H), 1.32-1.29 (m, 1H), 1.31 (s, 3H), 1.02 (ddd, *J* = 11.9, 11.9, 11.9 Hz, 1H) (**Supplementary Figure 37**); ^{13}C NMR (151

MHz, $CDCl_3$): δ 170.3, 165.1, 162.4 (d, *J* = 248 Hz), 138.4, 138.1, 134.6, 133.1 (d, *J* = 8 Hz), 130.3, 129.1, 128.9, 128.4, 128.3 (d, *J* = 3 Hz), 126.5, 123.8, 121.6, 119.9, 117.1, 115.6 (d, *J* = 21 Hz), 98.8, 80.8, 66.3, 66.0, 42.6, 40.8, 37.7, 36.1, 33.0, 30.1, 29.6, 29.3, 28.2, 19.8, 18.9; FTIR (ATR): υ 3409, 1725, 1667, 1595, 1526, 1509 cm^{-1}; HR-MS (ESI+): *m/z* calculated for $C_{41}H_{47}N_2O_5FNa$ $[M+Na]^+$: 689.3367, found: 689.3372 (**Supplementary Figure 38**).

Synthesis of 1,1-dimethylethyl (4*R*,6*R*)-6-[2-[5-cyclopentyl-2-(4-fluorophenyl)-3-phenyl-4-[(phenylamino)carbonyl]-1*H*-pyrrol-1-yl]ethyl]-2,2-dimethyl-1,3-dioxane-4-acetate (26)

Prepared using General Procedure B (white solid, 1.20 g, 62%; R_f 0.32 (20% EtOAc/hexanes)), followed by General Procedure C (pale yellow resin, 910 mg, 58%). R_f 0.44 (20% EtOAc/hexanes); ^{1}H NMR (600 MHz, $CDCl_3$): δ 7.21-7.15 (m, 9H), 7.06-7.04 (m, 2H), 7.00-6.96 (m, 3H), 6.85 (bs, 1H), 4.17-4.13 (m, 1H), 4.11-4.06 (m, 1H), 3.83 (ddd, *J* = 14.5, 10.5, 5.9 Hz, 1H), 3.69-3.64 (m, 1H), 3.64-3.58 (m, 1H), 2.38 (dd, *J* = 15.3, 7.0 Hz, 1H), 2.23 (dd, *J* = 15.3, 6.2 Hz, 1H), 2.16-2.11 (m, 2H), 2.09-2.01 (m, 2H), 1.99-1.92 (m, 2H), 1.73-1.58 (m, 4H), 1.43 (s, 9H), 1.36 (s, 3H), 1.33 (ddd, *J* = 12.7, 2.4, 2.4 Hz, 1H), 1.30 (s, 3H), 1.04 (ddd, *J* = 11.9, 11.9, 11.9 Hz, 1H) (**Supplementary Figure 39**); ^{13}C NMR (151 MHz, $CDCl_3$): δ 170.3, 164.8, 162.4 (d, *J* = 248 Hz), 139.6, 138.6, 134.8, 133.2 (d, *J* = 8 Hz), 130.7, 129.3, 128.8, 128.5, 128.4 (d, *J* = 3 Hz), 126.7, 123.6, 122.0, 119.6, 115.8, 115.5 (d, *J* = 21 Hz), 98.8, 80.8, 66.5, 66.0, 42.6, 41.0, 38.1, 37.1, 36.1, 32.7, 32.5, 30.0, 28.2, 26.6, 26.5, 19.8; FTIR (ATR): υ 3409, 1726, 1666, 1595, 1525, 1509 cm^{-1}; HR-MS (ESI+): *m/z* calculated for $C_{42}H_{49}N_2O_5FNa$ $[M+Na]^+$: 703.3523, found: 703.3519 (**Supplementary Figure 40**).

Synthesis of 1,1-dimethylethyl (4*R*,6*R*)-6-[2-[5-cyclohexyl-2-(4-fluorophenyl)-3-phenyl-4-[(phenylamino)carbonyl]-1*H*-pyrrol-1-yl]ethyl]-2,2-dimethyl-1,3-dioxane-4-acetate (27)

Prepared using General Procedure B (cream solid, 600 mg, 43%; R_f 0.36 (20% EtOAc/hexanes)), followed by General Procedure C (off-white resin, 295 mg, 33%). R_f 0.40 (20% EtOAc/hexanes); ^{1}H NMR (400 MHz, $CDCl_3$): δ 7.21-7.13 (m, 9H), 7.10-7.08 (m, 2H), 7.01-6.97 (m, 3H), 6.85 (bs, 1H), 4.20-4.13 (m, 1H), 4.12-4.05 (m, 1H), 3.84-3.76 (m, 1H), 3.72-3.66 (m, 1H), 3.11-3.05 (m, 1H), 2.39 (dd, *J* = 15.3, 6.9 Hz, 1H), 2.25 (dd, *J* = 15.3, 6.2 Hz, 1H), 2.24-2.13 (m, 2H), 1.88-1.85 (m, 4H), 1.68-1.59 (m, 2H), 1.44 (s, 9H), 1.40-1.34 (m, 6H), 1.33 (s, 3H), 1.08 (ddd, *J* = 11.9, 11.9, 11.9 Hz, 1H) (**Supplementary Figure 41**); ^{13}C NMR (101 MHz, $CDCl_3$): δ 170.3, 165.2, 162.4 (d, *J* = 248 Hz), 140.3, 138.6, 134.8, 133.3 (d, *J* = 8 Hz), 130.5, 128.8, 128.8, 128.5 (d, *J* = 3 Hz), 128.4, 126.5, 123.7, 121.9, 119.8, 116.0, 115.5 (d, *J* = 21 Hz), 98.8, 80.8, 66.5, 66.1, 42.6, 40.9, 38.4, 37.2, 36.2, 31.7, 31.6, 30.2, 28.2, 27.5, 25.9, 19.8; FTIR (ATR): υ 3409, 1727, 1667, 1595, 1525, 1509 cm^{-1}; HR-MS (ESI+): *m/z* calculated for $C_{43}H_{51}N_2O_5FNa$ $[M+Na]^+$: 717.3680, found: 717.3683 (**Supplementary Figure 42**).

Synthesis of 1-9 General Procedure D[6]

To a stirred solution of the 1,1-dimethylethyl (4*R*,6*R*)-6-[2-[5-(alkyl)-2-(4-fluorophenyl)-3-phenyl-4-[(phenylamino)carbonyl]-1*H*-pyrrol-1-yl]ethyl]-2,2-dimethyl-1,3-dioxane-4-acetate **19**-**21**, **23**-**27** (0.75 mmol, 1.0 equiv) in methanol (12 mL) was added 1M HCl (1.1 mL, 1.1 mmol, 1.5 equiv). After 1.5 h at r.t., the solution was purified by silica gel chromatography (20-50% EtOAc/hexanes), then dissolved in methanol (5 mL) and 1M NaOH (1.5 mL, 1.5 mmol, 2.0 equiv) was added. The solution was stirred at r.t. for 2 h, then a solution of calcium acetate hydrate (72 mg, 0.41 mmol, 0.55 equiv) in water (1 mL) was added

dropwise. After stirring for an additional 15 min, the precipitate was filtered, washed with water (3 x 5 mL) and dried to yield the compound of interest. (**Supplementary Figure 7 and 9**)

Synthesis of (β*R*,δ*R*)-5-cyclopropyl-2-(4-fluorophenyl)-β,δ-dihydroxy-3-phenyl-4-[(phenylamino)carbonyl]-1*H*-pyrrole-1-heptanoic acid hemicalcium salt (1)
Prepared using General Procedure D (white powder, 272 mg, 41%). ^{1}H NMR (600 MHz, DMSO-d_6): δ 10.02 (s, 1H), 7.62-7.61 (m, 2H), 7.27-7.22 (m, 4H), 7.20-7.17 (m, 2H), 7.10-7.07 (m, 2H), 7.07-6.99 (m, 4H), 5.95 (bs, 1H), 4.68 (bs, 1H), 4.13-4.08 (m, 1H), 4.00-3.95 (m, 1H), 3.79-3.76 (m, 1H), 3.57-3.54 (m, 1H), 2.07 (dd, *J* = 15.3, 4.1 Hz, 1H), 1.97-1.92 (m, 2H), 1.66-1.60 (m, 1H), 1.57-1.51 (m, 1H), 1.42-1.37 (m, 1H), 1.26-1.22 (m, 1H), 0.87-0.85 (m, 2H), 0.67-0.65 (m, 2H) (**Supplementary Figure 43**); ^{13}C NMR (151 MHz, DMSO-d_6): δ 178.0, 165.1, 161.6 (d, *J* = 245 Hz), 139.6, 134.9, 133.2 (d, *J* = 8 Hz), 132.0, 129.3, 128.5, 128.3, 127.6, 125.3, 122.9, 120.2, 119.3, 119.0, 115.4 (d, *J* = 21 Hz), 66.3, 66.3, 44.0, 43.8, 41.1, 38.1, 6.6, 5.9, 5.9; FTIR (ATR): υ 3399, 1650, 1594, 1558, 1509 cm^{-1}; HR-MS (ESI-): *m/z* calculated for $C_{33}H_{32}N_2O_5F$ [M-0.5Ca]$^-$: 555.2295, found: 555.2299 (**Supplementary Figure 44**).

Synthesis of (β*R*,δ*R*)-2-(4-fluorophenyl)-β,δ-dihydroxy-5-(1,1-dimethylethyl)-3-phenyl-4-[(phenylamino)carbonyl]-1*H*-pyrrole-1-heptanoic acid hemicalcium salt (2)
Prepared using General Procedure D (white powder, 27 mg, 34%). ^{1}H NMR (600 MHz, DMSO-d_6): δ 10.16 (s, 1H), 7.52-7.50 (m, 2H), 7.22-7.15 (m, 6H), 7.07-7.03 (m, 4H), 6.99-6.97 (m, 2H), 6.13 (bs, 1H), 4.72 (bs, 1H), 4.29-4.25 (m, 1H), 4.07-4.03 (m, 1H), 3.60-3.57 (m, 1H), 3.42-3.38 (m, 1H), 1.99 (dd, *J* = 14.9, 2.9 Hz, 1H), 1.85 (dd, *J* = 14.9, 8.0 Hz, 1H), 1.50 (s, 9H), 1.41-1.32 (m, 2H), 1.30-1.23 (m, 1H), 1.05-1.03 (m, 1H) (**Supplementary Figure 45**); ^{13}C NMR (151 MHz, DMSO-d_6): δ 177.7, 167.5, 161.5 (d, *J* = 245 Hz), 139.4, 135.2, 134.9, 133.2 (d, *J* = 8 Hz), 129.6, 129.2, 128.9 (d, *J* = 3 Hz), 128.4, 127.5, 125.4, 123.0, 120.8, 119.5, 118.9, 115.4 (d, *J* = 21 Hz), 66.4, 66.2, 43.8, 42.8, 38.2, 33.4, 30.8; FTIR (ATR): υ 3317, 1661, 1594, 1563, 1508 cm^{-1}; HR-MS (ESI-): *m/z* calculated for $C_{34}H_{36}N_2O_5F$ [M-0.5Ca]$^-$: 571.2613, found: 571.2611 (**Supplementary Figure 46**).

Synthesis of (β*R*,δ*R*)-2-(4-fluorophenyl)-β,δ-dihydroxy-3-phenyl-4-[(phenylamino)carbonyl-5-propyl]-1*H*-pyrrole-1-heptanoic acid hemicalcium salt (3)
Prepared using General Procedure D (white powder, 107 mg, 24%). ^{1}H NMR (600 MHz, DMSO-d_6): δ 9.28 (s, 1H), 7.43-7.42 (m, 2H), 7.26-7.04 (m, 11H), 6.98-6.96 (m, 1H), 6.03 (bs, 1H), 4.77 (bs, 1H), 3.97-3.93 (m, 1H), 3.81-3.73 (m, 2H), 3.54-3.50 (m, 1H), 2.85-2.82 (m, 2H), 2.08-2.06 (m, 1H), 1.95-1.91 (m, 1H), 1.64-1.53 (m, 3H), 1.49-1.43 (m, 1H), 1.41-1.36 (m, 1H), 1.23-1.19 (m, 1H), 0.93 (t, *J* = 6.7 Hz, 3H) (**Supplementary Figure 47**); ^{13}C NMR (151 MHz, DMSO-d_6): δ 178.0, 164.8, 161.6 (d, *J* = 245 Hz), 139.5, 134.8, 133.9, 133.3 (d, *J* = 8 Hz), 129.6, 128.5, 128.3, 127.7, 125.6, 122.8, 120.8, 119.2, 117.1, 115.4 (d, *J* = 21 Hz), 66.3, 66.1, 43.9, 43.7, 40.8, 38.6, 26.6, 23.4, 14.2; FTIR (ATR): υ 3396, 1660, 1594, 1558, 1530 cm^{-1}; HR-MS (ESI-): *m/z* calculated for $C_{33}H_{34}N_2O_5F$ [M-0.5Ca]$^-$: 557.2416, found: 557.2433 (**Supplementary Figure 48**).

Synthesis of (β*R*,δ*R*)-2-(4-fluorophenyl)-β,δ-dihydroxy-5-(2-hydroxyethyl)-3-phenyl-4-[(phenylamino)carbonyl]-1*H*-pyrrole-1-heptanoic acid hemicalcium salt (4)
To a solution of 1,1-dimethylethyl (4*R*,6*R*)-6-[5-(2-benzyloxyethyl)-2-[2-(4-fluorophenyl)-3-phenyl-4-[(phenylamino)carbonyl]-1*H*-pyrrol-1-yl]ethyl]-2,2-dimethyl-1,3-dioxane-4-acetate (**22**) (100 mg, 0.134 mmol) in methanol (2 mL) was added 1M HCl (0.30 mL) and the solution was stirred at r.t. for 2 h. The solution was diluted with EtOAc (10 mL) and washed with water (2 x 5 mL), dried over $MgSO_4$, filtered and concentrated, then purified by silica

gel chromatography (20-100% EtOAc/hexanes) to yield 1,1-dimethylethyl (3*R*,5*R*)-7-[5-(2-benzyloxyethyl)-2-(4-fluorophenyl)-3-phenyl-4-phenylcarbamoylpyrrol-1-yl]-3,5-dihydroxyheptanoate as a colourless resin (68 mg, 72%). R_f 0.17 (40% EtOAc/hexanes); ^{1}H NMR (400 MHz, $CDCl_3$): δ 7.40 (s, 1H), 7.33-7.29 (m, 4H), 7.28-7.22 (m, 6H), 7.19-7.14 (m, 4H), 7.10-7.08 (m, 2H), 7.01-6.95 (m, 3H), 4.58 (s, 2H), 4.14-3.97 (m, 3H), 3.93 (t, *J* = 6.1 Hz, 2H), 3.69-3.63 (m, 1H), 3.46 (t, *J* = 6.1 Hz, 2H), 3.11 (bs, 2H), 2.30-2.27 (m, 2H), 1.65-1.51 (m, 2H), 1.46 (s, 9H), 1.38 (ddd, *J* = 14.2, 10.1, 10.1 Hz, 1H), 1.18 (ddd, *J* = 14.2, 2.2, 2.2 Hz, 1H) (**Supplementary Figure 49**); ^{13}C NMR (101 MHz, $CDCl_3$): δ 172.2, 163.8, 162.4 (d, *J* = 248 Hz), 138.7, 138.5, 135.7, 134.9, 133.1 (d, *J* = 8 Hz), 131.2, 130.1, 128.8, 128.6, 128.5, 128.2 (d, *J* = 3 Hz), 127.8, 127.7, 127.2, 123.3, 122.0, 119.4, 115.5 (d, *J* = 21 Hz), 115.3, 81.8, 73.1, 70.7, 69.4, 69.1, 42.4, 41.9, 40.9, 38.8, 28.2, 26.5(**Supplementary Figure 50**).

To 1,1-dimethylethyl (3*R*,5*R*)-7-[5-(2-benzyloxyethyl)-2-(4-fluorophenyl)-3-phenyl-4-phenylcarbamoylpyrrol-1-yl]-3,5-dihydroxyheptanoate (68 mg, 0.096 mmol) and 20% $Pd(OH)_2/C$ (8.4 mg) was added ethanol (2 mL), then the atmosphere was evacuated and filled with H_2 (x 3) and stirred vigorously under a balloon H_2 for 5 h. The mixture was filtered through celite and purified by silica gel chromatography (50-100% EtOAc/hexanes; R_f 0.48 (EtOAc)) to yield a colourless resin, which was dissolved in methanol (0.3 mL) and 1M NaOH (0.1 mL) was added. The solution was stirred at r.t. for 1 h, then a solution of calcium acetate hydrate (8 mg, 0.04 mmol, 0.6 equiv) in water (0.5 mL) was added dropwise. After stirring for an additional 30 min, the precipitate was filtered, washed with water (5 x 1 mL) and dried to yield the title compound as a cream solid (22 mg, 39%). ^{1}H NMR (600 MHz, DMSO-d_6): δ 9.71 (s, 1H), 7.49-7.47 (m, 2H), 7.27-7.22 (m, 4H), 7.19-7.16 (m, 2H), 7.13-7.10 (m, 2H), 7.07-7.04 (m, 3H), 7.00-6.97 (m, 1H), 6.18 (bs, 1H), 5.44 (bt, J = 4.5 Hz, 1H), 4.73 (bd, *J* = 3.5 Hz, 1H), 4.00-3.95 (m, 1H), 3.86-3.80 (m, 1H), 3.73-3.71 (m, 2H), 3.53-3.49 (m, 1H), 3.05 (t, *J* = 6.5 Hz, 2H), 2.04 (dd, *J* = 15.1, 4.4 Hz, 1H), 1.92 (dd, *J* = 15.1, 7.8 Hz, 1H), 1.58-1.52 (m, 1H), 1.50-1.43 (m, 1H), 1.40-1.35 (m, 1H), 1.24-1.20 (m, 1H) (**Supplementary Figure 51**); ^{13}C NMR (151 MHz, DMSO-d_6): δ 177.6, 164.2, 161.6 (d, *J* = 245 Hz), 139.5, 134.3, 133.3 (d, *J* = 8 Hz), 130.7, 129.7, 129.0, 128.5, 128.5 (d, *J* = 3 Hz), 127.6, 125.6, 122.9, 121.4, 119.1, 118.1, 115.3 (d, *J* = 21 Hz), 66.3, 66.1, 60.9, 44.0, 43.9, 40.9, 38.4, 28.3; FTIR (ATR): ʋ 3393, 1641, 1594, 1558, 1532 cm^{-1}; HR-MS (ESI+): *m/z* calculated for $C_{32}H_{34}N_2O_6F$ $[M-0.5Ca+2H]^+$: 561.2401, found: 561.2390 (**Supplementary Figure 52**).

Synthesis of (β*R*,δ*R*)-2-(4-fluorophenyl)-β,δ-dihydroxy-5-(2-methylpropyl)-3-phenyl-4-[(phenylamino)carbonyl]-1*H*-pyrrole-1-heptanoic acid hemicalcium salt (5)

Prepared using General Procedure D (white powder, 206 mg, 46%). ^{1}H NMR (600 MHz, DMSO-d_6): δ 9.39 (s, 1H), 7.44-7.43 (m, 2H), 7.25-7.16 (m, 6H), 7.12-7.10 (m, 2H), 7.05-7.01 (m, 3H), 6.98-6.96 (m, 1H), 5.89 (bs, 1H), 4.74 (bs, 1H), 3.98-3.95 (m, 1H), 3.83-3.77 (m, 1H), 3.75-3.72 (m, 1H), 3.50-3.46 (m, 1H), 2.78-2.77 (m, 2H), 2.06 (dd, *J* = 15.2, 3.4 Hz, 1H), 1.93 (dd, *J* = 15.2, 8.0 Hz, 1H), 1.90-1.83 (m, 1H), 1.52-1.46 (m, 1H), 1.43-1.34 (m, 2H), 1.21-1.16 (m, 1H), 0.92-0.90 (m, 6H) (**Supplementary Figure 53**); ^{13}C NMR (151 MHz, DMSO-d_6): δ 178.2, 165.0, 161.6 (d, *J* = 245 Hz), 139.5, 134.8, 133.3 (d, *J* = 8 Hz), 132.8, 129.5, 128.6 (d, *J* = 3 Hz), 128.5, 127.7, 125.5, 122.8, 120.9, 119.3, 117.8, 115.4 (d, *J* = 21 Hz), 66.2, 66.0, 43.9, 43.7, 40.9, 38.4, 33.3, 29.2, 22.5, 22.5; FTIR (ATR): ʋ 3404, 1728, 1662, 1595, 1528 cm^{-1}; HR-MS (ESI-): *m/z* calculated for $C_{34}H_{36}N_2O_5F$ $[M-0.5Ca]^-$: 571.2608, found: 571.2611 (**Supplementary Figure 54**).

Synthesis of (β*R*,δ*R*)-5-butyl-2-(4-fluorophenyl)-β,δ-dihydroxy-3-phenyl-4-[(phenylamino)carbonyl]-1*H*-pyrrole-1-heptanoic acid hemicalcium salt (6)
Prepared using General Procedure D (white powder, 372 mg, 84%). ^{1}H NMR (500 MHz, DMSO-d_6): δ 9.29 (s, 1H), 7.44-7.42 (m, 2H), 7.26-7.16 (m, 6H), 7.14-7.11 (m, 2H), 7.07-7.04 (m, 3H), 6.98-6.95 (m, 1H), 5.95 (bs, 1H), 4.75 (bs, 1H), 3.98-3.92 (m, 1H), 3.82-3.73 (m, 2H), 3.54-3.49 (m, 1H), 2.87-2.84 (m, 2H), 2.09-2.05 (m, 1H), 1.93 (dd, *J* = 15.2, 8.1 Hz, 1H), 1.61-1.52 (m, 3H), 1.49-1.43 (m, 1H), 1.42-1.31 (m, 3H), 1.23-1.18 (m, 1H), 0.86 (t, *J* = 7.3 Hz, 3H) (**Supplementary Figure 55**); ^{13}C NMR (126 MHz, DMSO-d_6): δ 178.1, 164.7, 161.6 (d, *J* = 245 Hz), 139.5, 134.8, 134.0, 133.3 (d, *J* = 8 Hz), 129.6, 128.5 (d, *J* = 3 Hz), 128.5, 128.3, 127.7, 125.6, 122.8, 120.8, 119.2, 117.1, 115.4 (d, *J* = 21 Hz), 66.3, 66.1, 43.9, 43.7, 40.8, 38.6, 32.2, 24.2, 22.1, 13.7; FTIR (ATR): ʋ 3403, 1662, 1594, 1558, 1531 cm^{-1}; HR-MS (ESI+): *m/z* calculated for $C_{34}H_{38}N_2O_5F$ [M-0.5Ca+2H]$^{+}$: 573.2765, found: 573.2761 (**Supplementary Figure 56**).

Synthesis of (β*R*,δ*R*)-5-cyclobutyl-2-(4-fluorophenyl)-β,δ-dihydroxy-5-cyclobutyl-3-phenyl-4-[(phenylamino)carbonyl]-1*H*-pyrrole-1-heptanoic acid hemicalcium salt (7)
Prepared using General Procedure D (off-white powder, 127 mg, 29%). ^{1}H NMR (500 MHz, DMSO-d_6): δ 10.06 (s, 1H), 7.59-7.57 (m, 2H), 7.25-7.16 (m, 6H), 7.10-7.05 (m, 4H), 7.02-6.98 (m, 2H), 5.93 (bs, 1H), 4.72 (bs, 1H), 3.94-3.87 (m, 1H), 3.80-3.69 (m, 3H), 3.53-3.49 (m, 1H), 2.42-2.27 (m, 4H), 2.09-2.05 (m, 1H), 1.96-1.85 (m, 2H), 1.71-1.66 (m, 1H), 1.57-1.50 (m, 1H), 1.48-1.36 (m, 2H), 1.24-1.19 (m, 1H) (**Supplementary Figure 57**); ^{13}C NMR (126 MHz, DMSO-d_6): δ 178.2, 165.9, 161.6 (d, *J* = 245 Hz), 139.5, 134.9, 133.3 (d, *J* = 8 Hz), 133.3, 129.2, 128.6 (d, *J* = 3 Hz), 128.5, 127.9, 127.6, 125.3, 123.0, 120.5, 119.3, 118.3, 115.4 (d, *J* = 21 Hz), 66.3, 66.2, 44.0, 43.8, 41.1, 38.5, 32.5, 28.9, 28.8, 18.5; FTIR (ATR): ʋ 3407, 1661, 1594, 1559, 1508 cm^{-1}; HR-MS (ESI-): *m/z* calculated for $C_{34}H_{34}N_2O_5F$ [M-0.5Ca]$^{-}$: 569.2452, found: 569.2448 (**Supplementary Figure 58**).

Synthesis of (β*R*,δ*R*)-5-cyclopentyl-2-(4-fluorophenyl)-β,δ-dihydroxy-3-phenyl-4-[(phenylamino)carbonyl]-1*H*-pyrrole-1-heptanoic acid hemicalcium salt (8)
Prepared using General Procedure D (white powder, 159 mg, 36%). ^{1}H NMR (600 MHz, DMSO-d_6): δ 9.76 (s, 1H), 7.49-7.47 (m, 2H), 7.25-7.16 (m, 6H), 7.09-7.05 (m, 4H), 7.00-6.96 (m, 2H), 5.95 (bs, 1H), 4.73 (bs, 1H), 3.98-3.94 (m, 1H), 3.81-3.74 (m, 2H), 3.55-3.51 (m, 1H), 3.27-3.22 (m, 1H), 2.09-1.92 (m, 6H), 1.71-1.65 (m, 2H), 1.63-1.49 (m, 4H), 1.42-1.37 (m, 1H), 1.26-1.21 (m, 1H) (**Supplementary Figure 59**); ^{13}C NMR (151 MHz, DMSO-d_6): δ 178.2, 166.0, 161.6 (d, *J* = 245 Hz), 139.4, 134.9, 133.9, 133.4 (d, *J* = 8 Hz), 129.2, 128.8 (d, *J* = 3 Hz), 128.4, 127.6, 127.6, 125.4, 123.0, 120.8, 119.4, 117.6, 115.4 (d, *J* = 21 Hz), 66.3, 66.2, 43.9, 43.7, 41.0, 36.6, 32.5, 25.5, 25.5; FTIR (ATR): ʋ 3395, 1652, 1594, 1558, 1508 cm^{-1}; HR-MS (ESI-): *m/z* calculated for $C_{35}H_{36}N_2O_5F$ [M-0.5Ca]$^{-}$: 583.2608, found: 583.2594 (**Supplementary Figure 60**).

Synthesis of (β*R*,δ*R*)-5-cyclohexyl-2-(4-fluorophenyl)-β,δ-dihydroxy-3-phenyl-4-[(phenylamino)carbonyl]-1*H*-pyrrole-1-heptanoic acid hemicalcium salt (9)
Prepared using General Procedure D (white powder, 57 mg, 23%). ^{1}H NMR (500 MHz, DMSO-d_6): δ 9.82 (s, 1H), 7.51-7.50 (m, 2H), 7.25-7.16 (m, 6H), 7.07-7.05 (m, 4H), 7.00-6.97 (m, 2H), 6.51 (bs, 1H), 4.80 (bs, 1H), 3.95-3.89 (m, 1H), 3.79-3.72 (m, 2H), 3.57-3.53 (m, 1H), 2.85-2.80 (m, 1H), 2.07-2.03 (m, 1H), 1.91-1.80 (m, 5H), 1.76-1.71 (m, 2H), 1.65-1.60 (m, 2H), 1.57-1.49 (m, 1H), 1.43-1.29 (m, 3H), 1.25-1.20 (m, 1H), 1.12-1.04 (m, 1H) (**Supplementary Figure 61**); ^{13}C NMR (126 MHz, DMSO-d_6): δ 177.2, 166.3, 161.6 (d, *J* = 245 Hz), 139.5, 135.1, 135.0, 133.4 (d, *J* = 8 Hz), 129.1, 128.8 (d, *J* = 3 Hz), 128.4, 127.6, 127.2, 125.3, 122.9, 120.5, 119.5, 117.8, 115.4 (d, *J* = 21 Hz), 66.4, 66.3, 43.8, 40.7, 36.3,

32.1, 26.7, 25.6; FTIR (ATR): υ 3395, 1652, 1594, 1558, 1508 cm^{-1}; HR-MS (ESI-): *m/z* calculated for $C_{36}H_{38}N_2O_5F$ $[M\text{-}0.5Ca]^-$: 597.2765, found: 597.2770 (**Supplementary Figure 62**).

Supplementary Table 1. HMGR sequences used for sequence conservation analysis.

HMGR in species	Group	% identity to HMGR	Accession code
Arabidopsis thaliana	Eudicot	100	P14891.1
Arabidopsis suecica	Eudicot	98	KAG7659775.1
Arabidopsis lyrata subsp lyrata	Eudicot	98	XP_002887642.2
Arabidopsis arenosa	Eudicot	98	CAE5964346.1
Capsella rubella	Eudicot	95	XP_006302046.1
Camelina sativa	Eudicot	94	XP_010428712.1
Eutrema salsugineum	Eudicot	92	XP_006390188.1
Brassica rapa	Eudicot	89	XP_009128179.1
Raphanus sativus	Eudicot	87	XP_018445978.1
Tarenaya hassleriana	Eudicot	84	XP_010537243.1
Eucalyptus grandis	Eudicot	80	KCW68146.1
Populus trichocarpa	Eudicot	78	XP_002301898.2
Ricinus communis	Eudicot	78	XP_002510732.1
Juglans regia	Eudicot	78	XP_018843042.2
Lupinus angustifolius	Eudicot	78	XP_019461234.1
Theobroma cacao	Eudicot	78	EOY15882.1
Daucus carota	Eudicot	78	XP_017253170.1
Jatropha curcas	Eudicot	77	XP_012073564.1
Cannabis sativa	Eudicot	77	XP_030495961.1
Vitis vinifera	Eudicot	77	XP_002275827.1
Citrus sinensis	Eudicot	76	XP_006473861.1
Glycine max	Eudicot	75	XP_003519474.1
Solanum tuberosum	Eudicot	75	XP_006342182.1
Rosa chinensis	Eudicot	75	XP_024163983.1
Medicago truncatula	Eudicot	75	XP_003617066.1
Gossypium barbadense	Eudicot	75	KAB2042521.1
Chenopodium quinoa	Eudicot	73	XP_021714065.1
Brachypodium distachyon	Monocot	73	XP_003572378.1
Spinacia oleracea	Eudicot	72	XP_021846881.1
Trifolium subterraneum	Eudicot	72	GAU28089.1
Ipomoea triloba	Eudicot	71	XP_031109081.1
Malus domestica	Eudicot	71	XP_008348952.1
Solanum lycopersicum	Eudicot	71	XP_010317674.1
Sorghum bicolor	Monocot	71	XP_002445887.1
Triticum turgidum	Monocot	70	VAI86078.1
Triticum aestivum	Monocot	70	XP_044433217.1
Zea mays	Monocot	70	NP_001130818.1
Oryza sativa	Monocot	70	AAD38873.1
Setaria viridis	Monocot	69	XP_034581600.1
Cocos nucifera	Monocot	68	KAG1358991.1
Pisum sativum	Eudicot	68	AAL37041.1
Digitaria exilis	Monocot	68	KAF8780957.1

Supplementary Figures

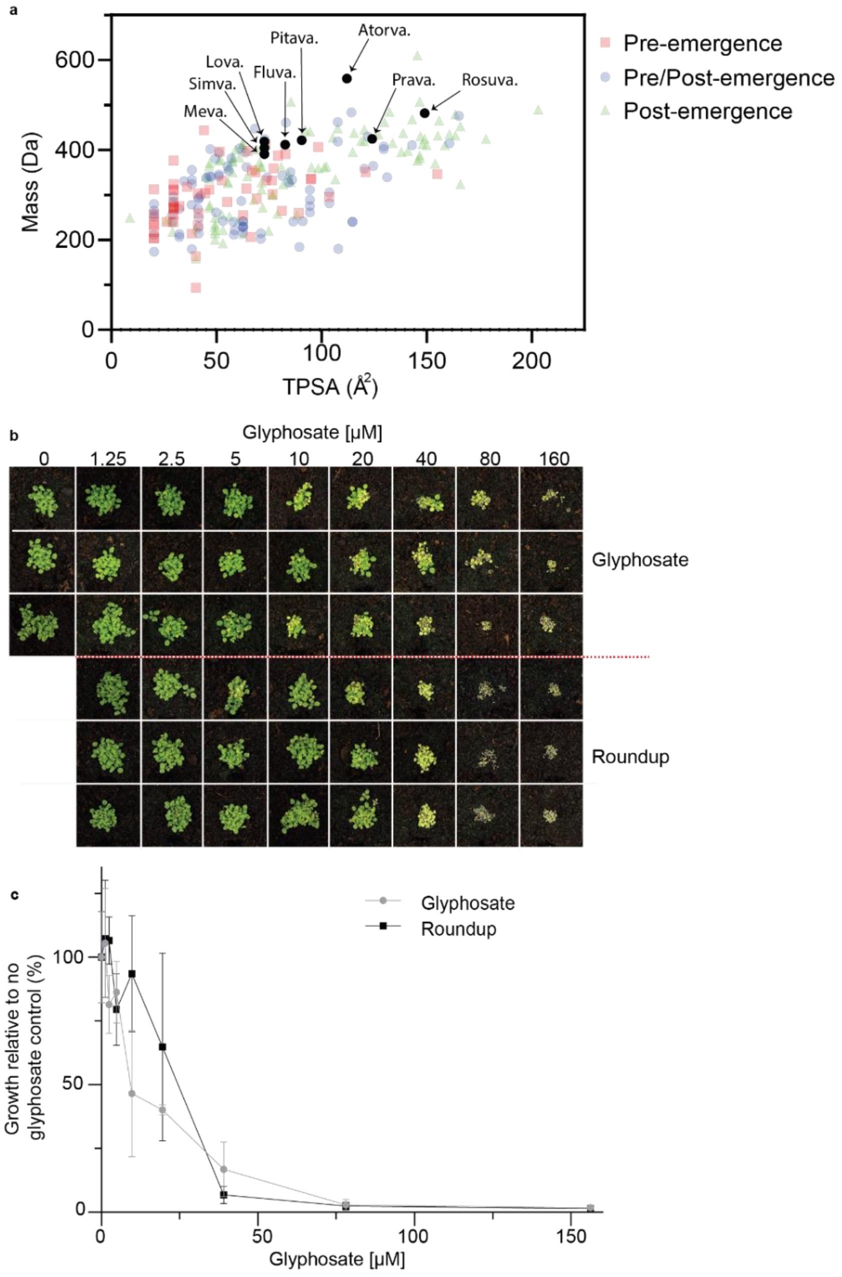

Supplementary Figure 1. Statins have similar physicochemical properties to post-emergence herbicides and an activity akin to glyphosate. Through analysis of 360 commercial herbicides[8], we were able to classify 55 as pre-emergence herbicides, 86 as both pre/post emergence herbicides and 103 as post-emergence herbicides. The physicochemical properties of these 244 herbicides were plotted and compared to the physicochemical properties of the commercially available statins (**a**). Pre-emergence herbicides tend to have a smaller mass and smaller topological polar surface area (TPSA) than post-emergence herbicides. (**b, c**) Post-emergence dose range of glyphosate formulated and diluted in 0.02% Brushwet or as Roundup® (360 g/L glyphosate) diluted in water, applied on *A. thaliana*. Images taken 12 days post-emergence (**b**) and quantified using ImageJ software (**c**) $n = 3$ replicates with the mean ± standard deviation (s.d.). Source data are provided as a Source Data file.

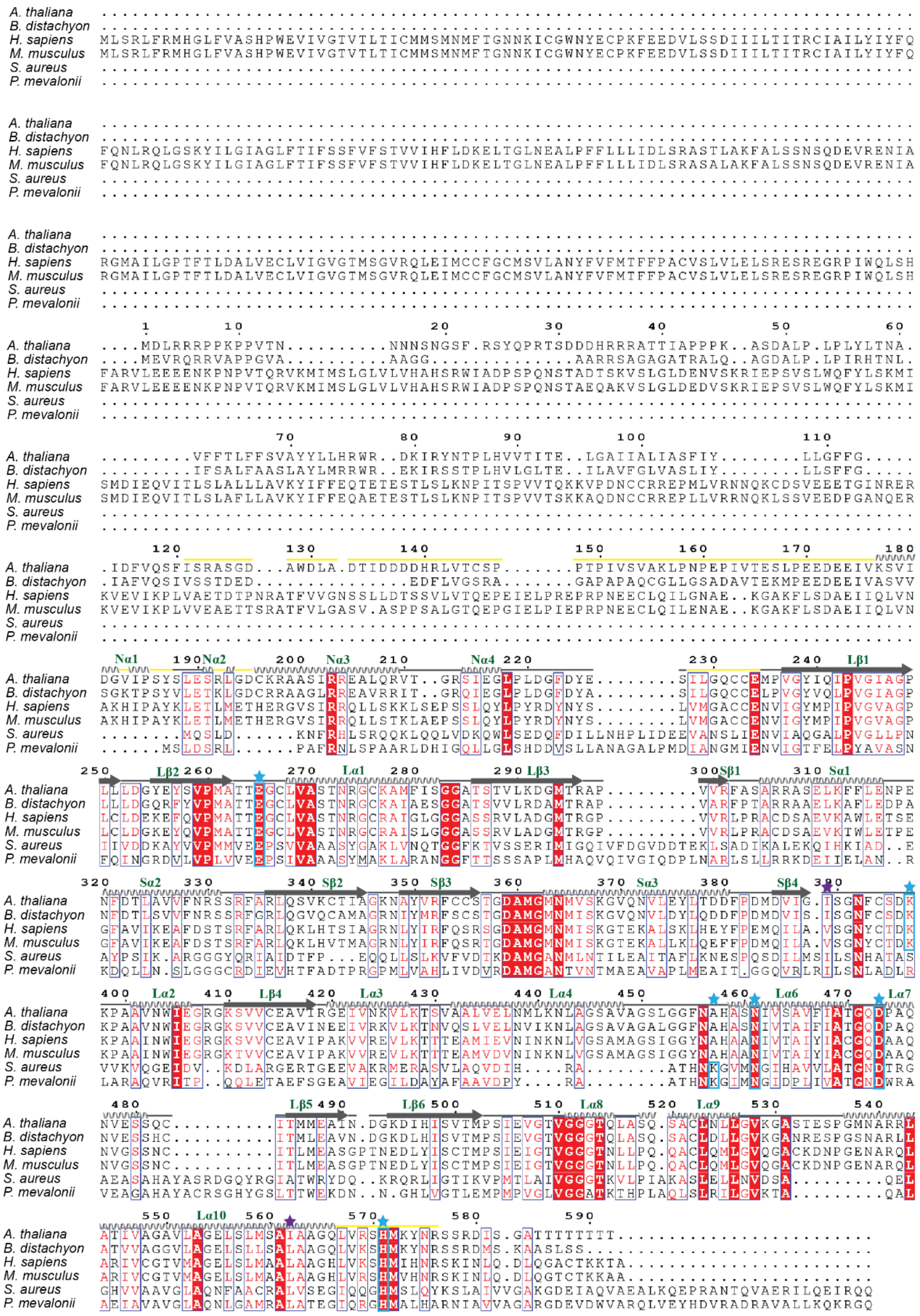

Supplementary Figure 2. Sequence alignment of AtHMG1 with class I and II HMGRs. Secondary structure elements of AtHMG1 extracellular core domain with labels (green), based on topology designation from human HMGR[9], are shown above sequence. Solid grey arrows indicate β-strands, grey helices indicate α-helices, grey lines indicate loop regions, yellow lines indicate regions lacking electron density in the *apo* structure. Conserved regions (red box), highly similar residues (red text)[10]. Residues implicated in catalysis from previous studies are highlighted with a cyan star and box. *A. thaliana* active site residues that are divergent from mammalian class I HMGRs are highlighted with a purple star. Sequences: *A. thaliana* P14891, *B. distachyon* I1I8I9, *H. sapiens* P04035, *M. musculus* Q01237, *S. aureus* CAC6866932.1, *P. mevalonii* P13702,

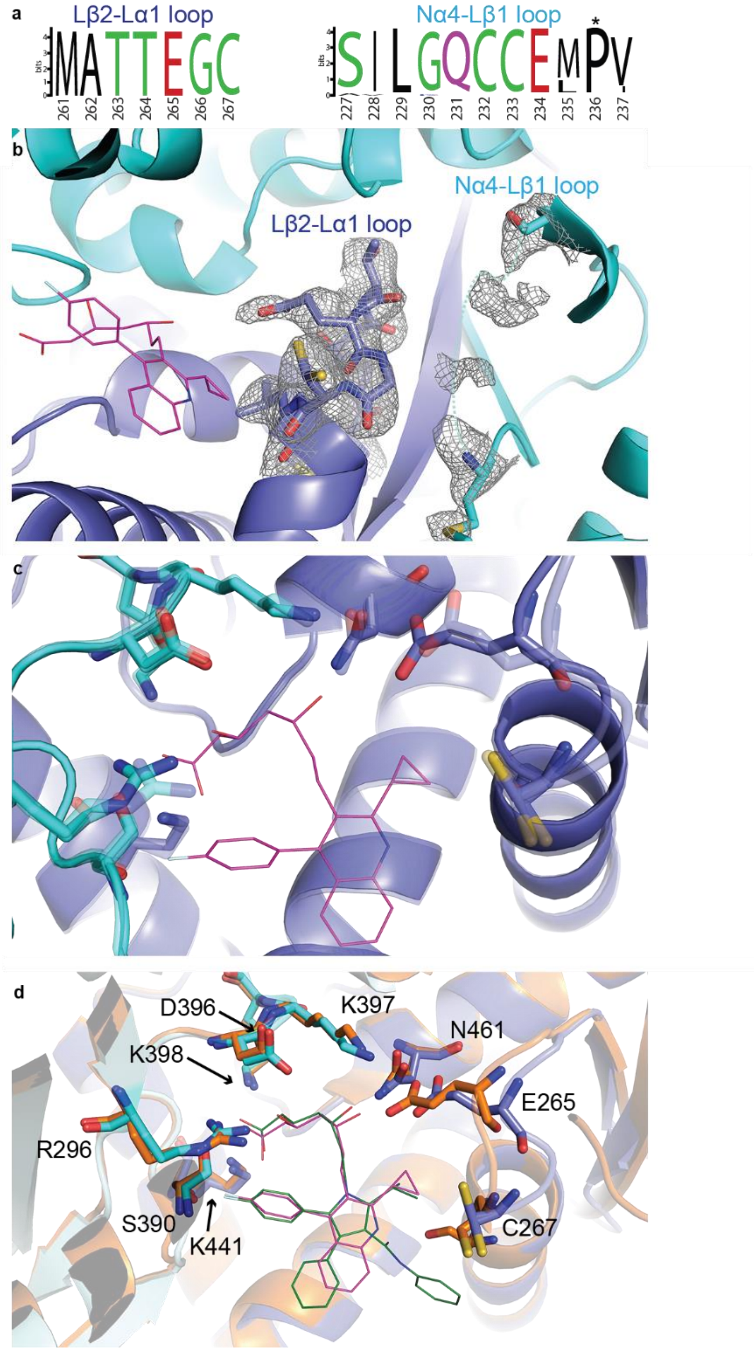

Supplementary Figure 3. A unique architecture of AtHMG1 could be targeted for the rational design of plant specific inhibitors. (**a**) Relative abundance of residues in the Lβ2-Lα1 and Nα4-Lβ1 loops from 40 plant species (**Supplementary Table 1**), illustrated using the WebLogo server[11]. Pro^{236} (highlighted with an asterisk) is conserved in diverse plant species. (**b**) Simulated annealing omit electron density maps (2 Fobs - Fcalc) contoured at 1σ level illustrate defined density for the apo AtHMG1 Lβ2-Lα1 loop and poorly defined density for the adjacent Nα4-Lβ1 loop. Pitavastatin (magenta line) is superimposed for reference. (**c**) Overlay of Apo (transparent, blue cartoon) and pitavastatin bound (blue cartoon) AtHMG1 active site delineating residues reveals a highly similar overall architecture and a slight shift of Glu^{265} towards the bound inhibitor. (**d**) HsHMGCR1 (PDB 1HWK, orange cartoon) superimposed onto AtHMG1 pitavastatin complex shows the isopropyl group on the central pyrrole ring of atorvastatin (green line) could be modified to target the unique architecture of AtHMG1 Lβ2-Lα1 loop region.

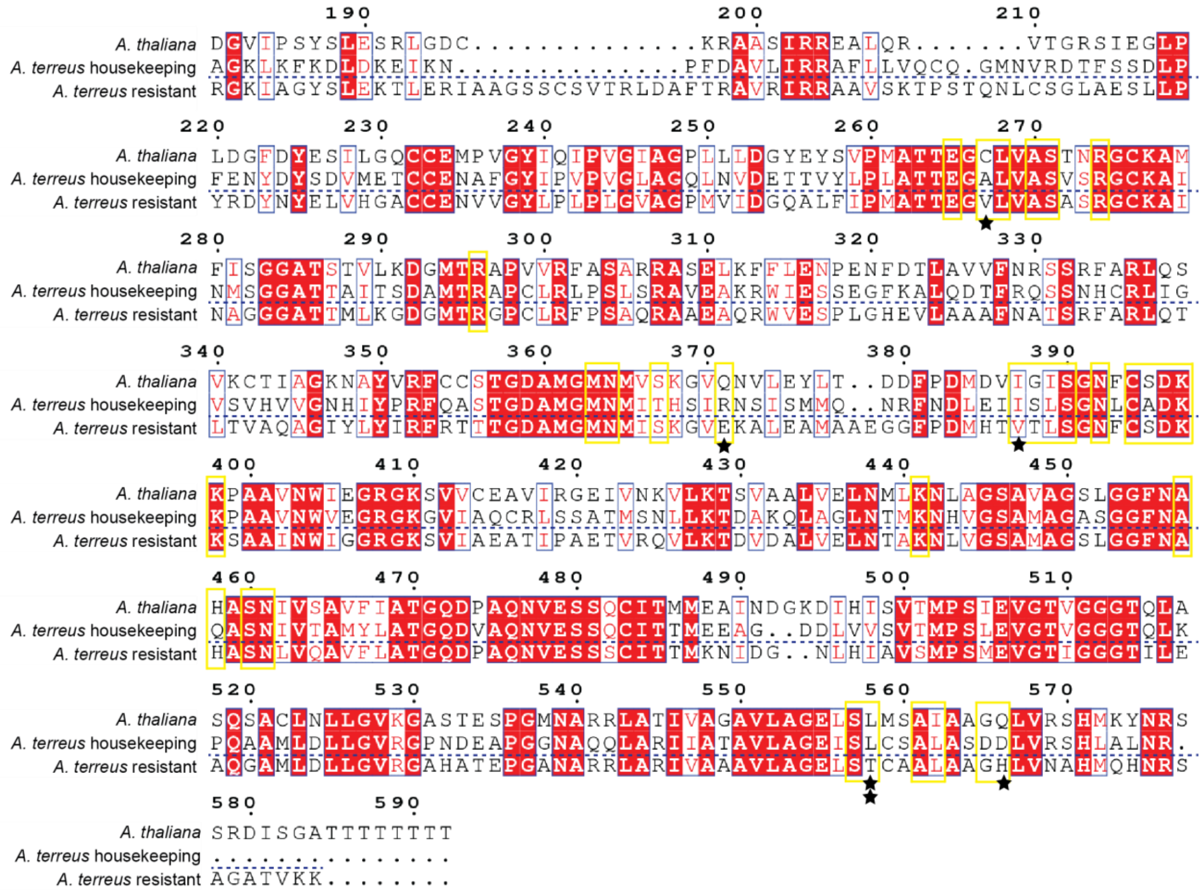

Supplementary Figure 4. Sequence alignment of AtHMG1 with *Aspergillus terreus* HMGR. Comparison of the sequences of AtHMG1 and *A. terreus* NIH2624 putative *HMGR* housekeeping gene (ATEG_02145) with *A. terreus* NIH2624 reported HMGR self-resistant gene[12-14](ATEG_09965) from the lovastatin biosynthetic gene cluster reveals several residues potentially conferring HMGR with statin resistance HMGR (black stars). AtHMG1 active site-delineating residues shown with yellow boxes. Sequence numbers shown for AtHMG1 residue L558 is highlighted with two stars. Conserved regions (red box), highly similar residues (red text)[10].

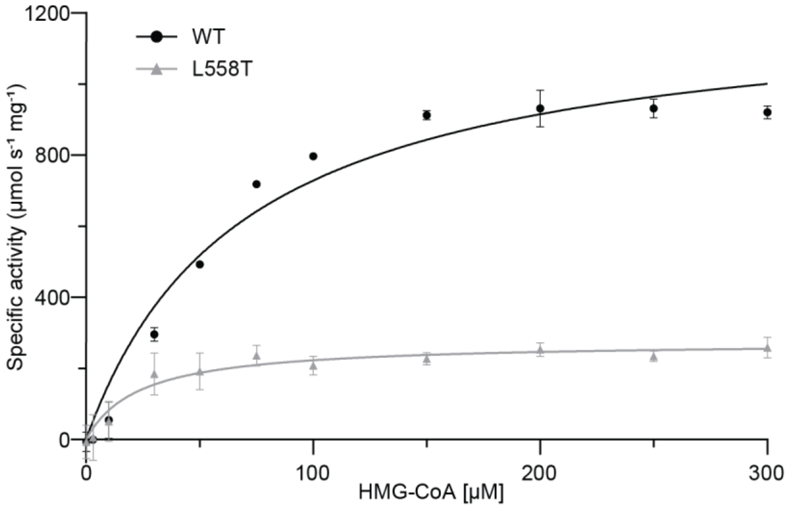

Supplementary Figure 5. Steady state kinetic data for AtHMG1 and L558T mutant. Substrate HMG-CoA saturation curve for WT (black circles) and L558T mutant (grey triangles) with Michaelis-Menten fit. *n* = 3 independent reactions with the mean ± s.d. Source data are provided as a Source Data file.

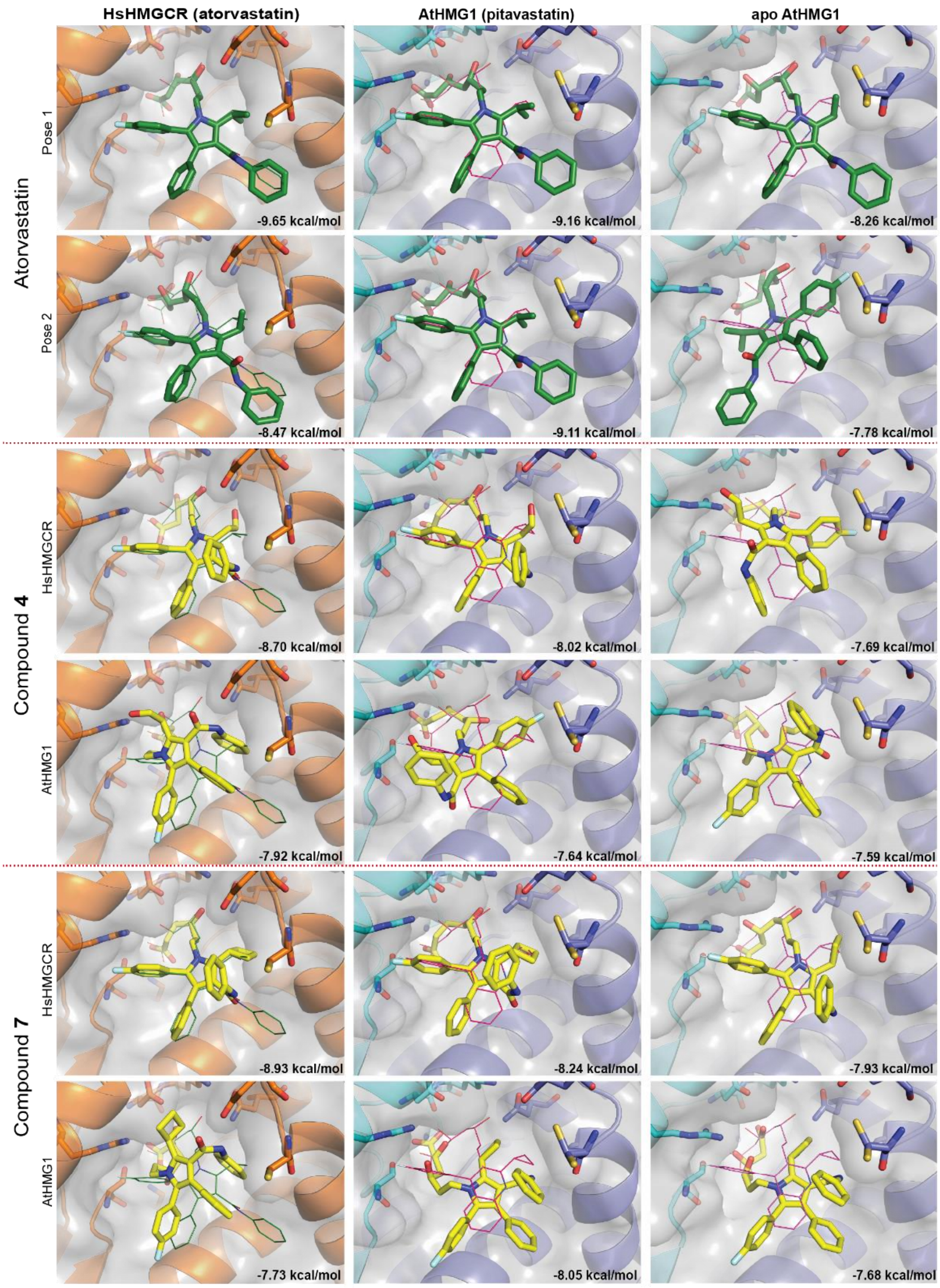

Supplementary Figure 6. Modelling of atorvastatin and AtHMG1-specific analogs binding to HsHMGCR and AtHMG1. Compounds were docked into HsHMGCR with atorvastatin ligand removed (1HWK, left column, orange cartoon), AtHMG1 with pitavastatin ligand removed (middle column, blue cartoon) and apo AtHMG1 (right column, blue cartoon) using GNINA software[15]. Active site-delineating residues are shown as sticks (orange and blue/cyan). Binding modes of atorvastatin (green line HsHMGCR) and pitavastatin (magenta line AtHMG1) from crystal structures are superimposed for reference. Top two binding poses by affinity are shown. Compounds **4** and **7** (yellow sticks) are predicted to bind HsHMGCR in a manner analogous to atorvastatin (green sticks). Modelling predicts more varied binding modes for AtHMG1 with lower affinity.

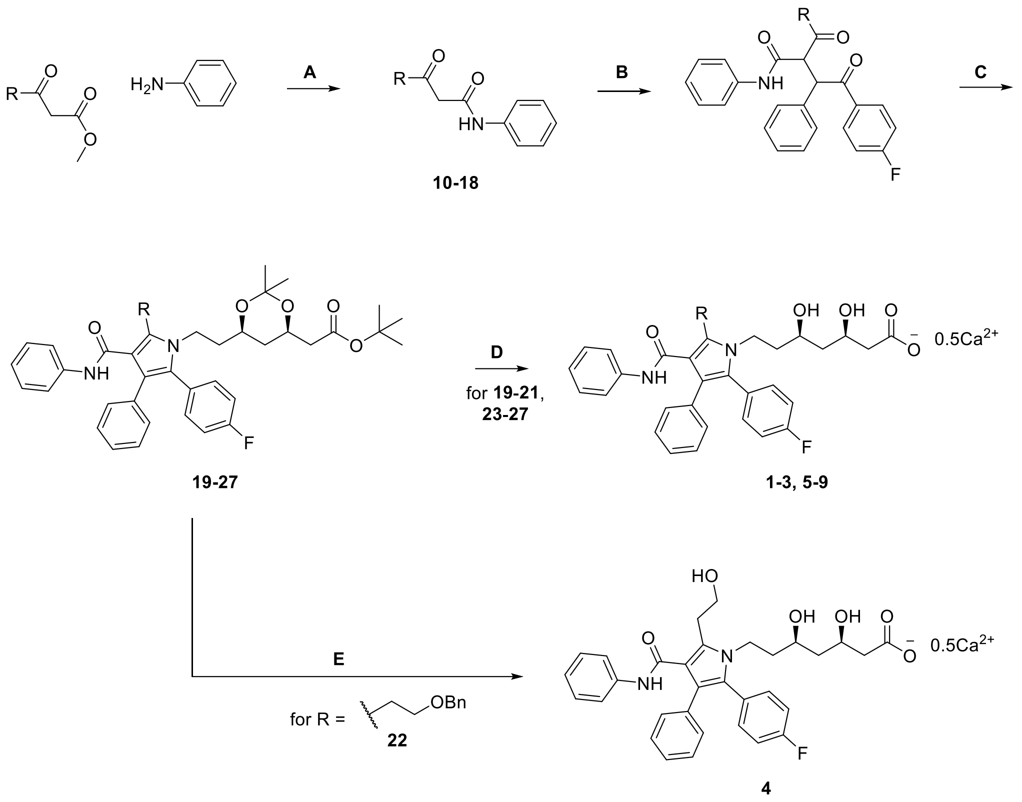

Supplementary Figure 7. Schematic of General Procedure A-E. A) TEA, toluene, Δ; B) 2-bromo-1-(3-fluorophenyl)-2-phenylethanone, K_2CO_3, acetone; C) (4*R*,6*R*)-*tert*-butyl-6-(2-aminoethyl)-2,2-dimethyl-1,3-dioxane-4-acetate, pivalic acid, 4:1:1 heptane/toluene/THF, Δ; D) i) HCl, MeOH; ii) NaOH, MeOH; iii) $Ca(OAc)_2.H_2O$; E) i) HCl, MeOH; ii) H_2, $Pd(OH)_2/C$, ethanol; iii) NaOH, MeOH; iv) $Ca(OAc)_2.H_2O$.

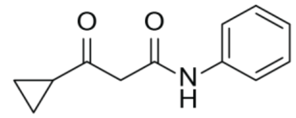

10

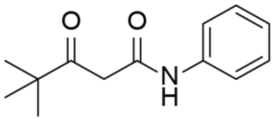

11

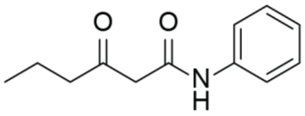

12

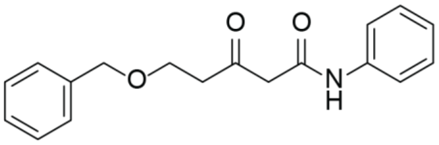

13

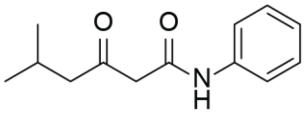

14

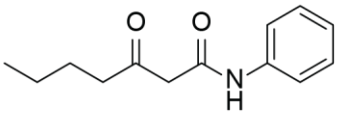

15

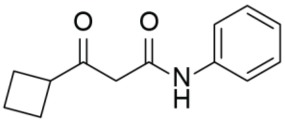

16

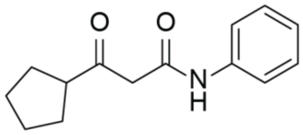

17

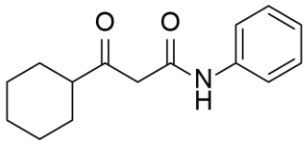

18

Supplementary Figure 8. Chemical structure of compounds 10-18.

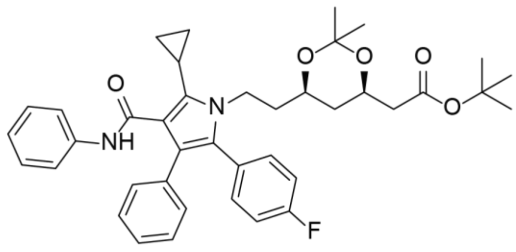

19

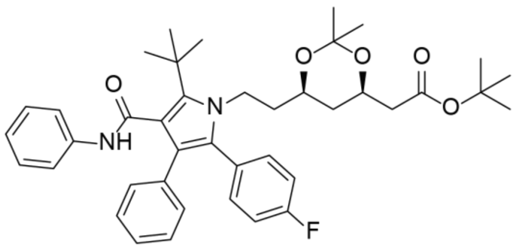

20

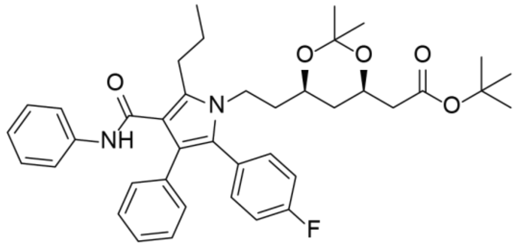

21

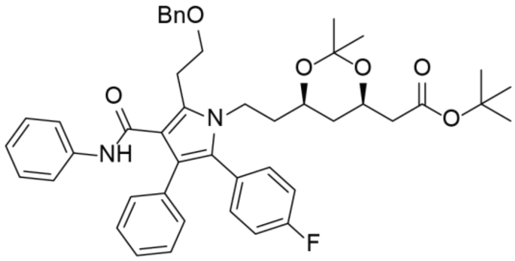

22

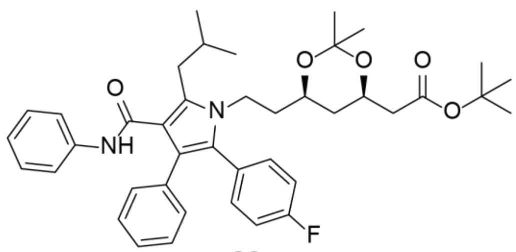

23

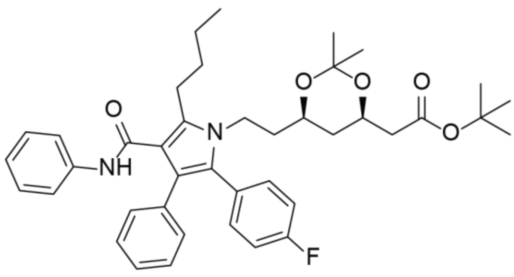

24

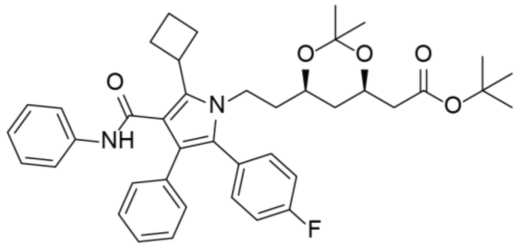

25

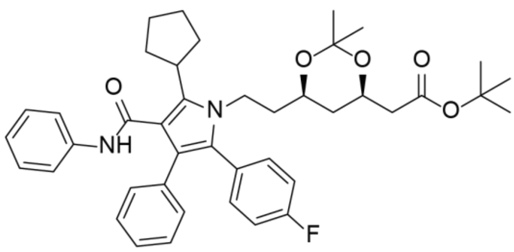

26

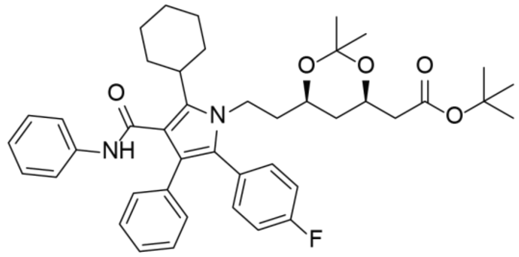

27

Supplementary Figure 9. Chemical structure of compounds 19-27.

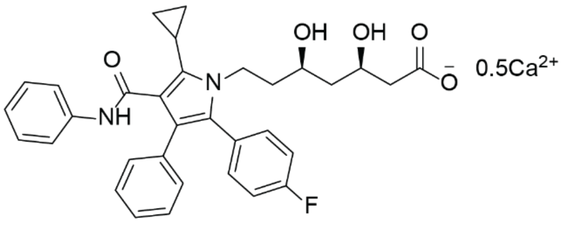

1

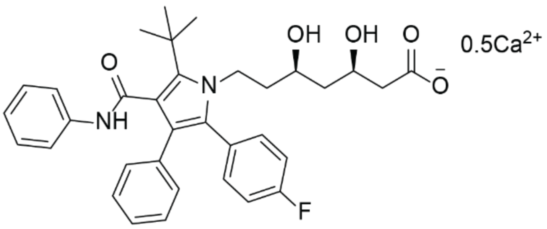

2

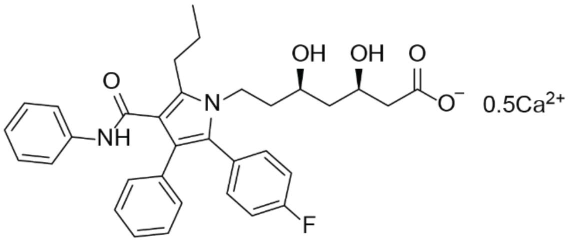

3

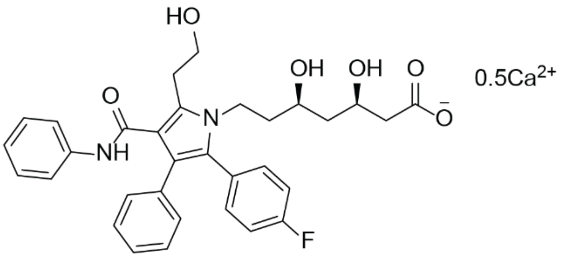

4

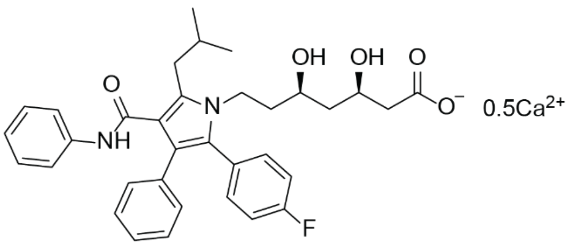

5

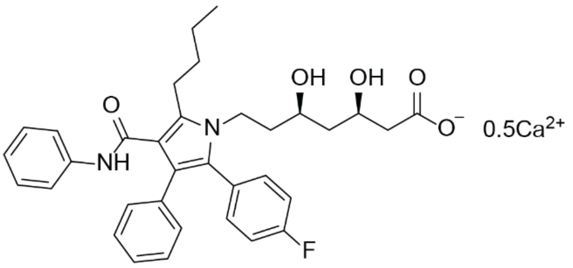

6

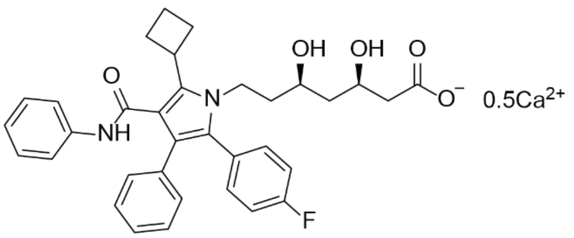

7

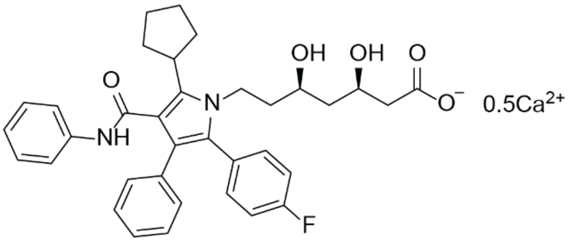

8

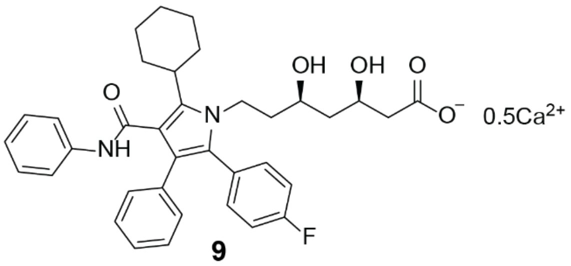

9

Supplementary Figure 10. Chemical structure of compounds 1-9.

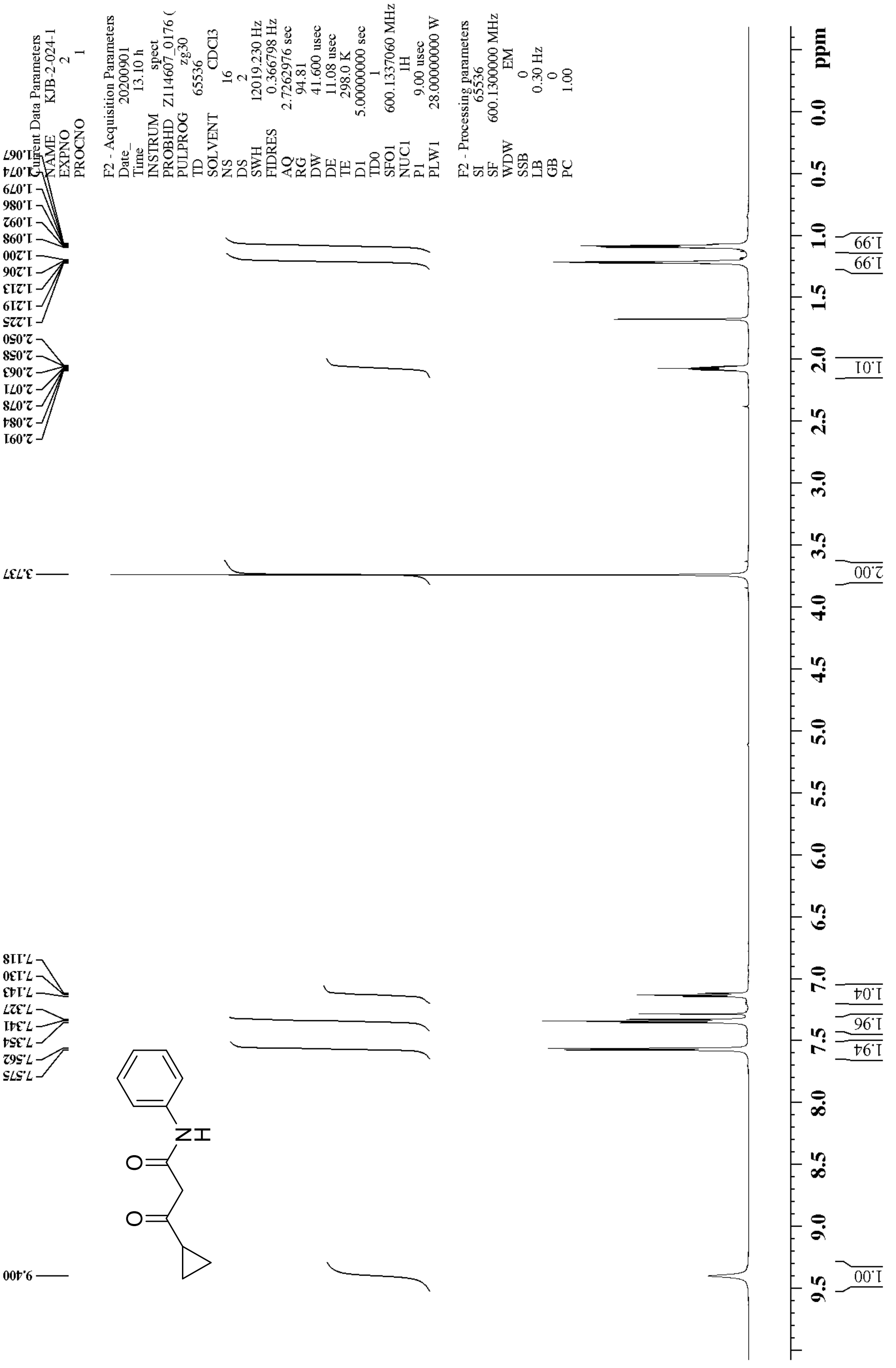

Supplementary Figure 11. ¹H NMR of β-oxo-N-phenylcyclopropanepropanamide (10).

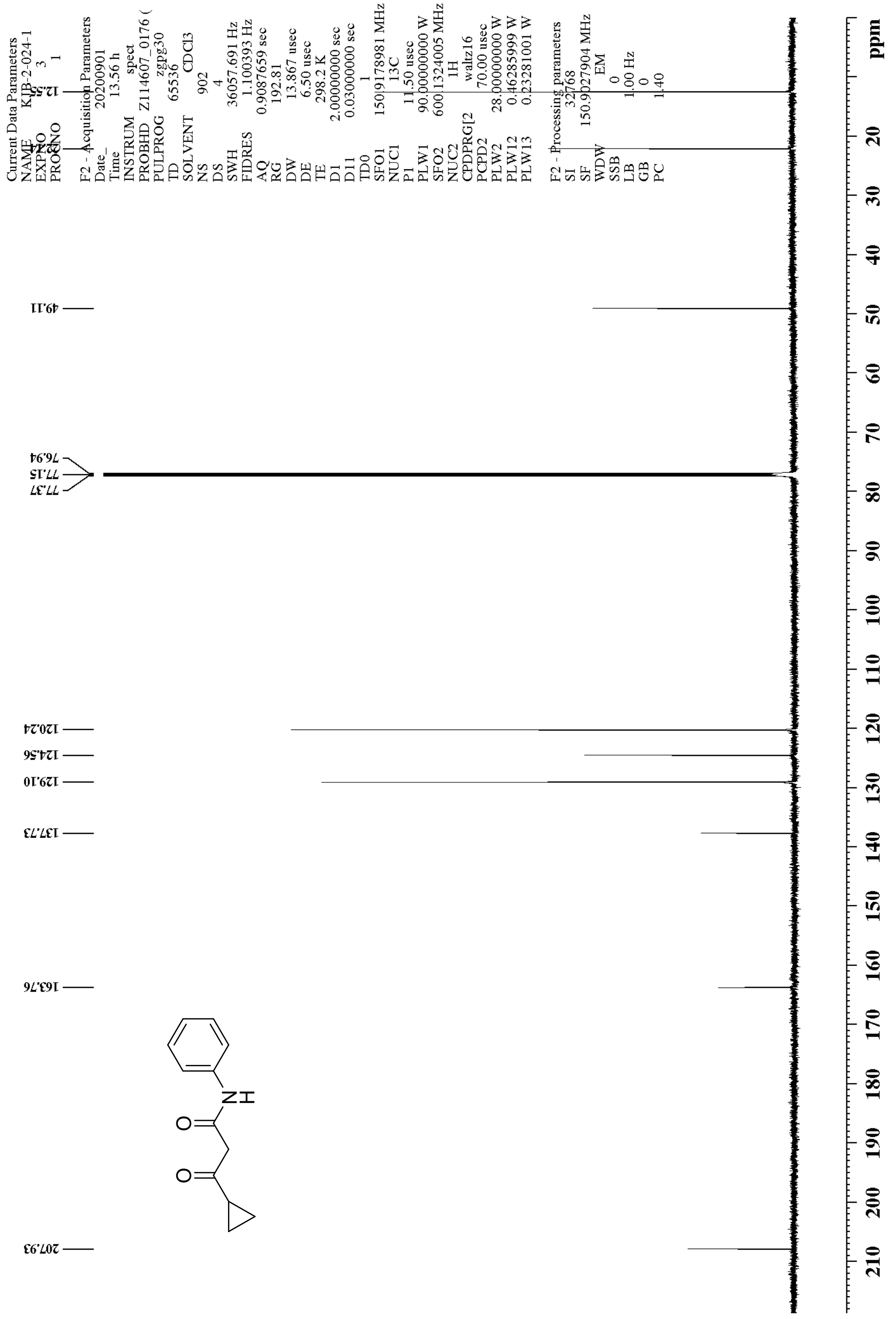

Supplementary Figure 12. ^{13}C NMR of β-oxo-*N*-phenylcyclopropanepropanamide (10).

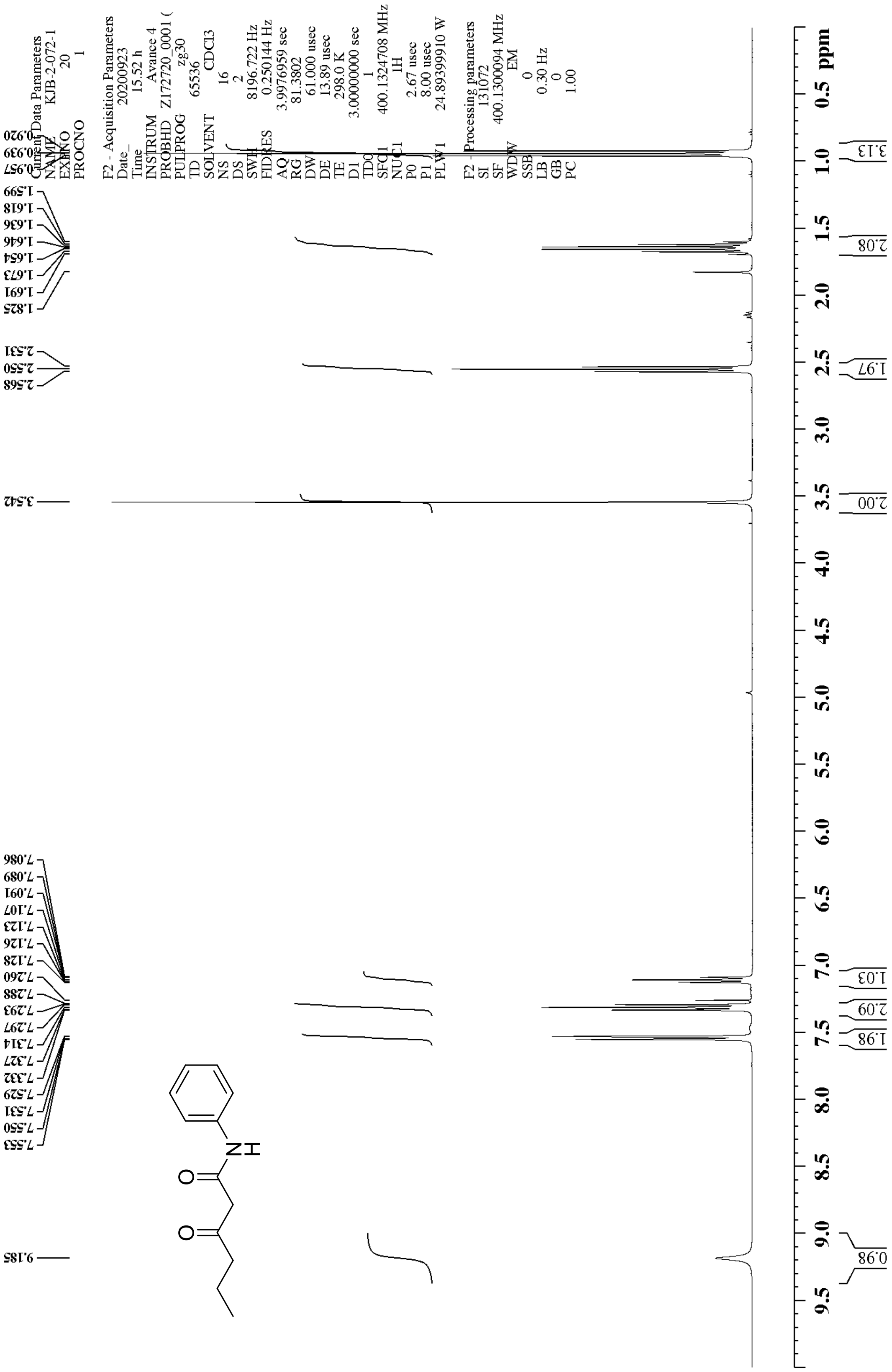

Supplementary Figure 13. ^{1}H NMR of 3-oxo-*N*-phenylhexanamide (12).

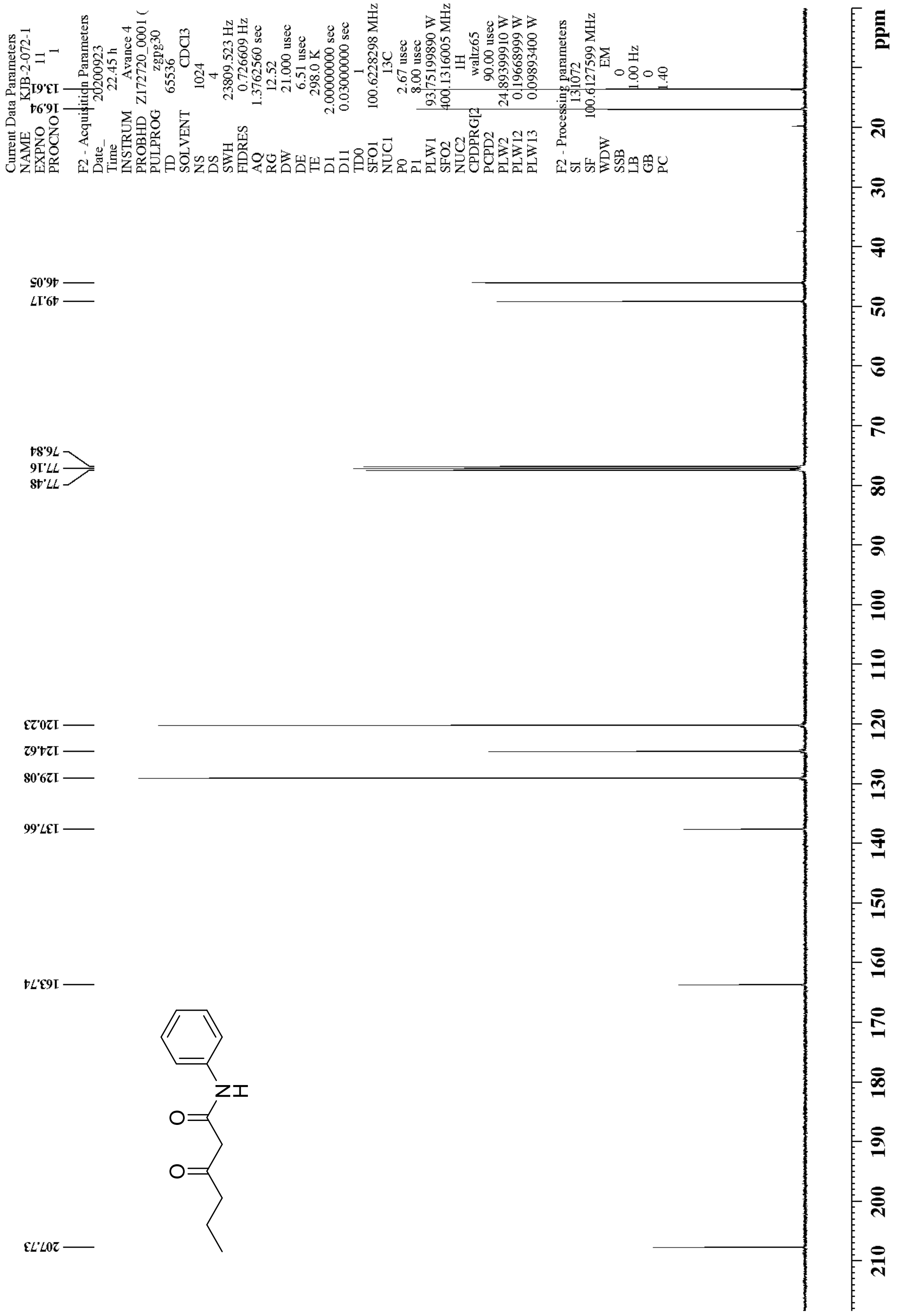

Supplementary Figure 14. ^{13}C **NMR of 3-oxo-*N*-phenylhexanamide (12).**

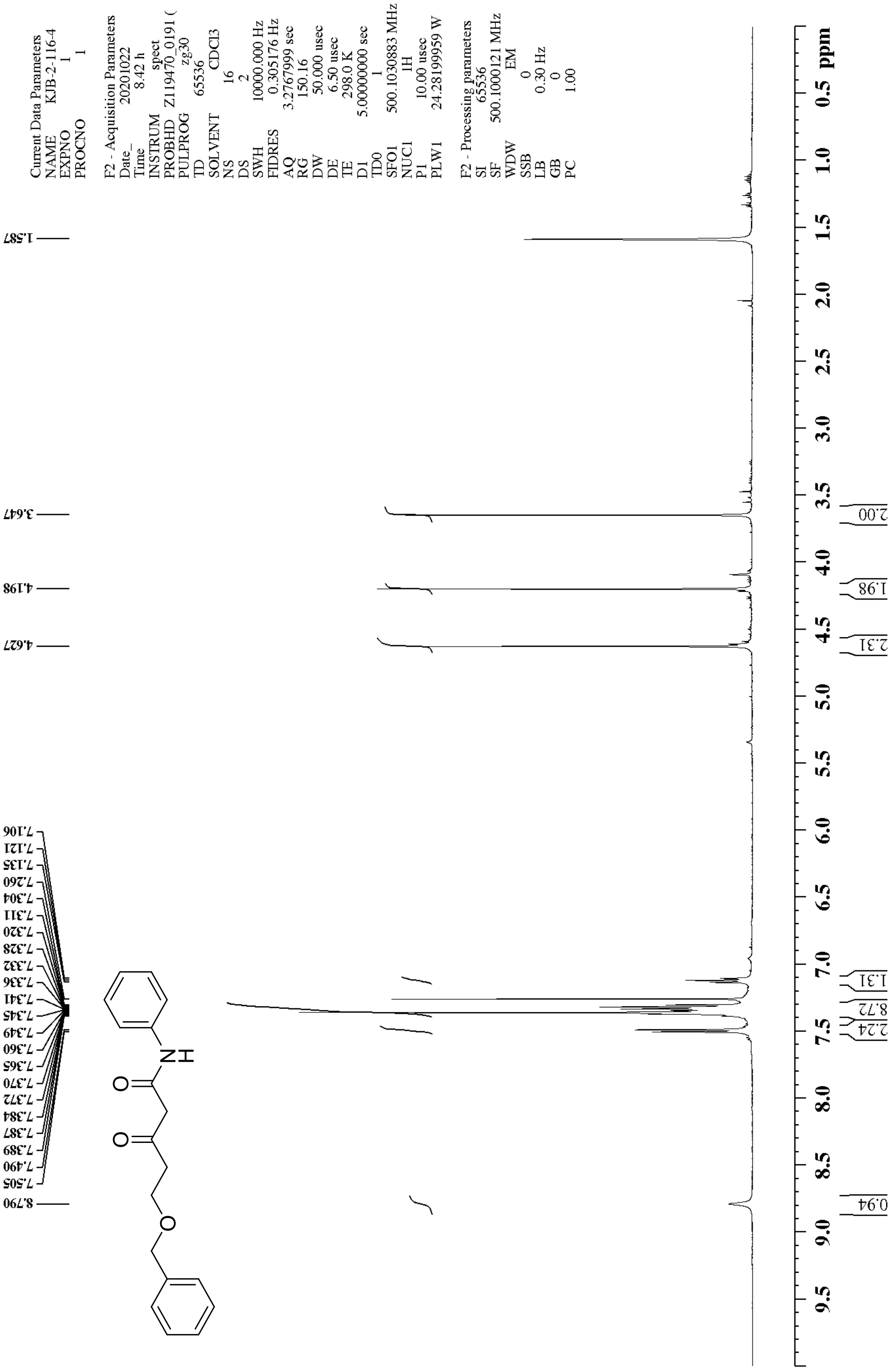

Supplementary Figure 15. ^{1}H NMR of 3-oxo-*N*-phenyl-5-(benzyloxy)pentanamide (13).

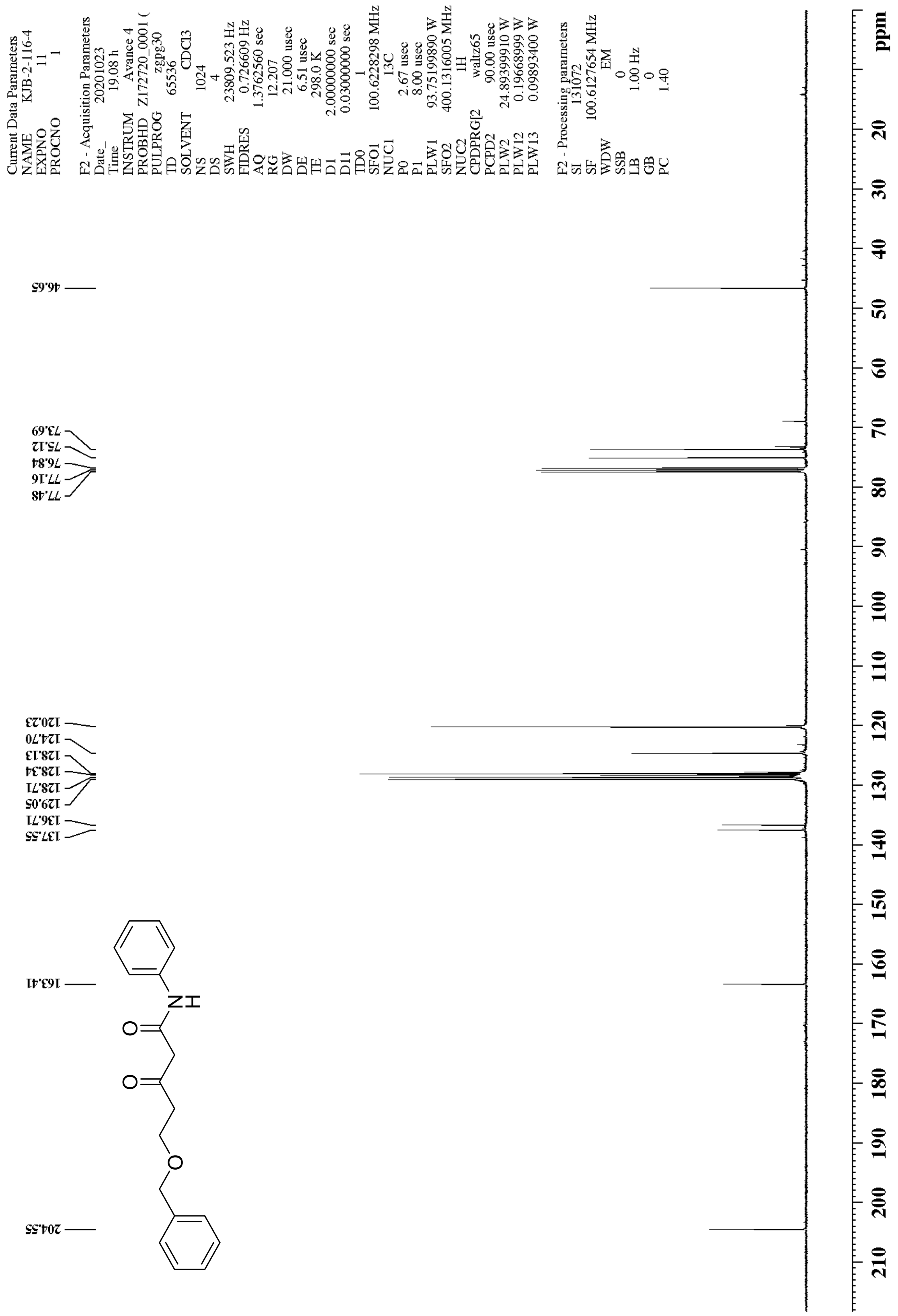

Supplementary Figure 16. ^{13}C NMR of 3-oxo-*N*-phenyl-5-(benzyloxy)pentanamide (13).

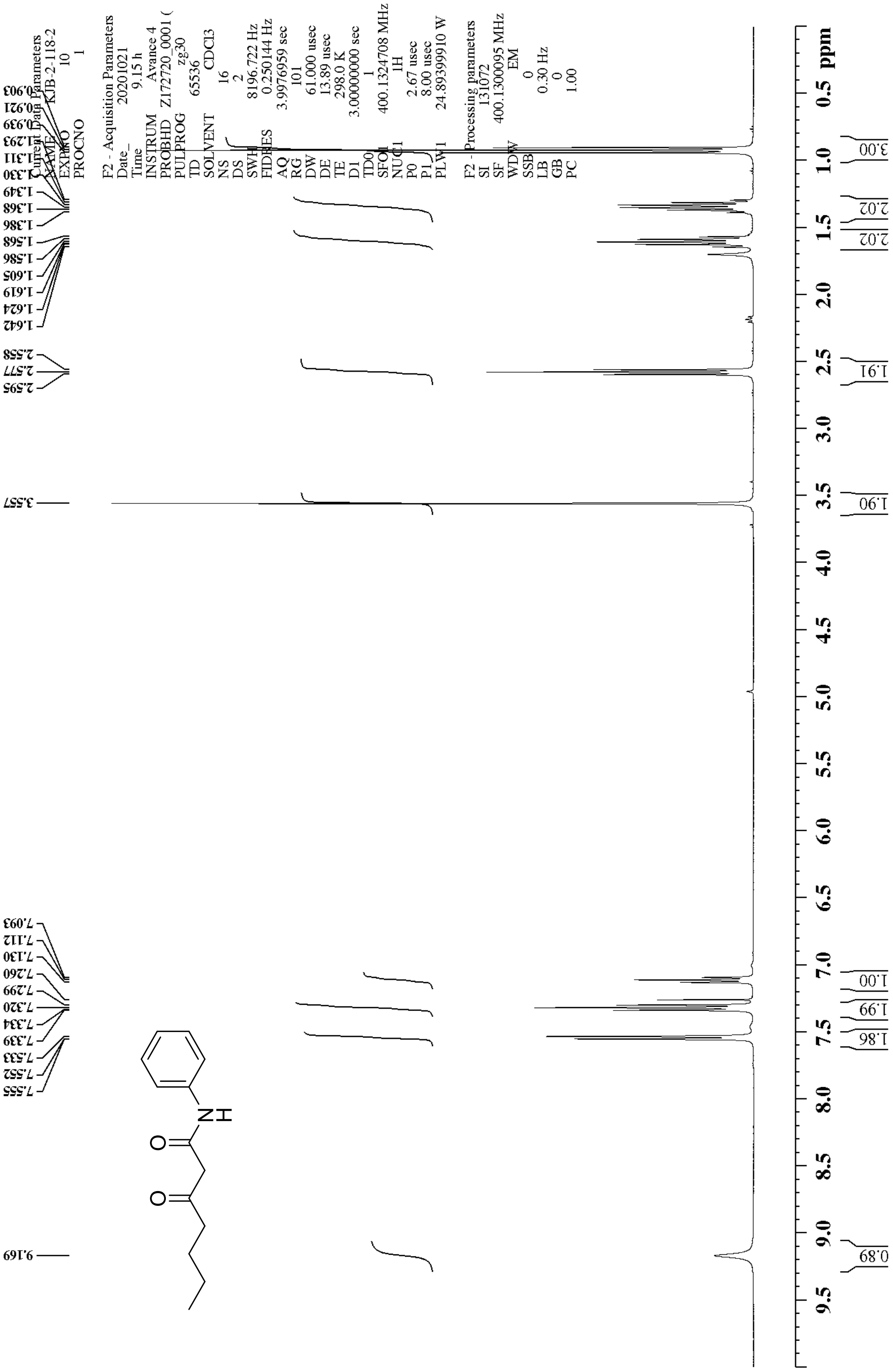

Supplementary Figure 17. ^{1}H NMR of 3-oxo-*N*-phenylheptanamide (15).

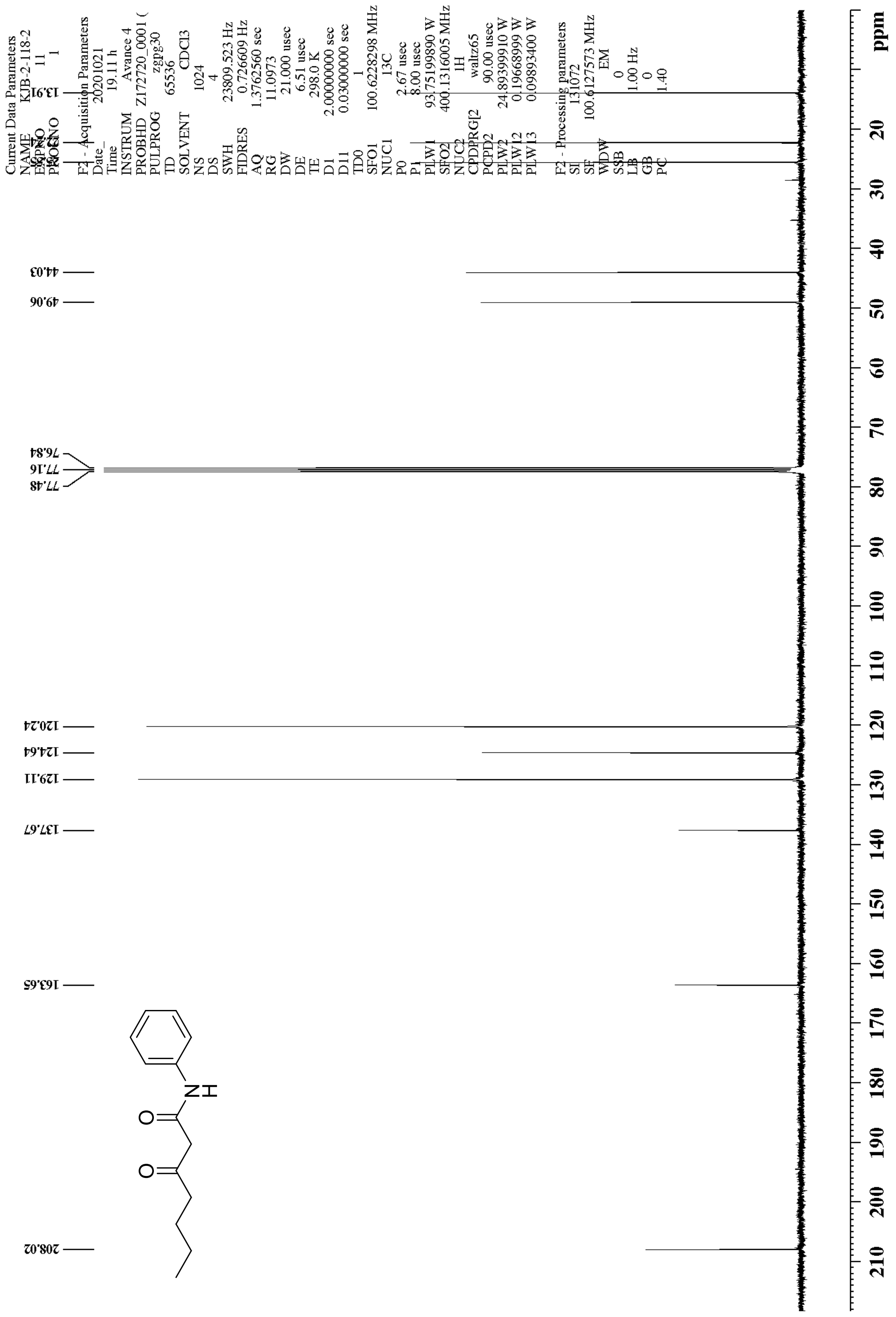

Supplementary Figure 18. ^{13}**C NMR of 3-oxo-*N*-phenylheptanamide (15).**

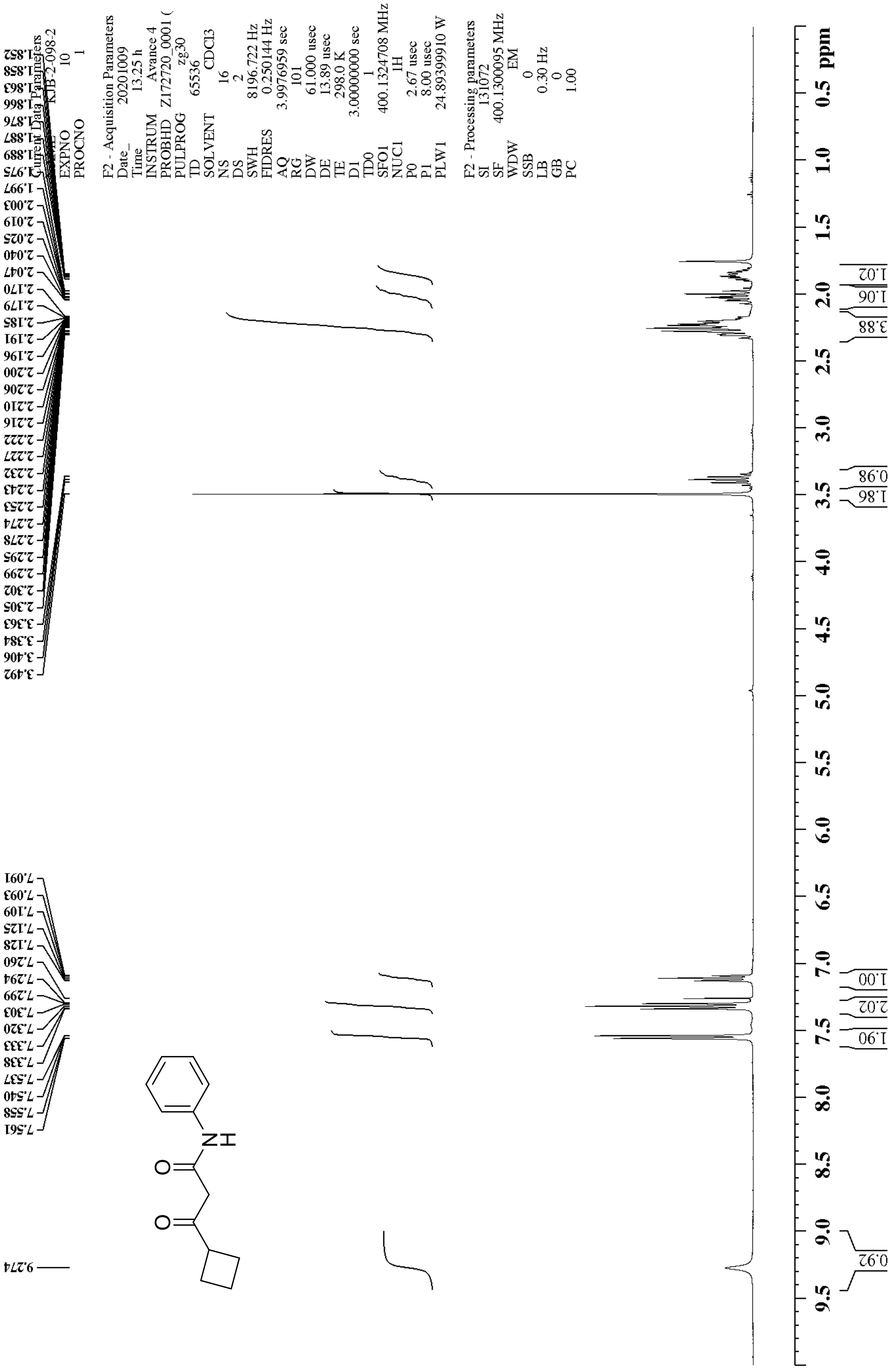

Supplementary Figure 19. ^{1}H NMR of β-oxo-*N*-phenylcyclobutanepropanamide (16).

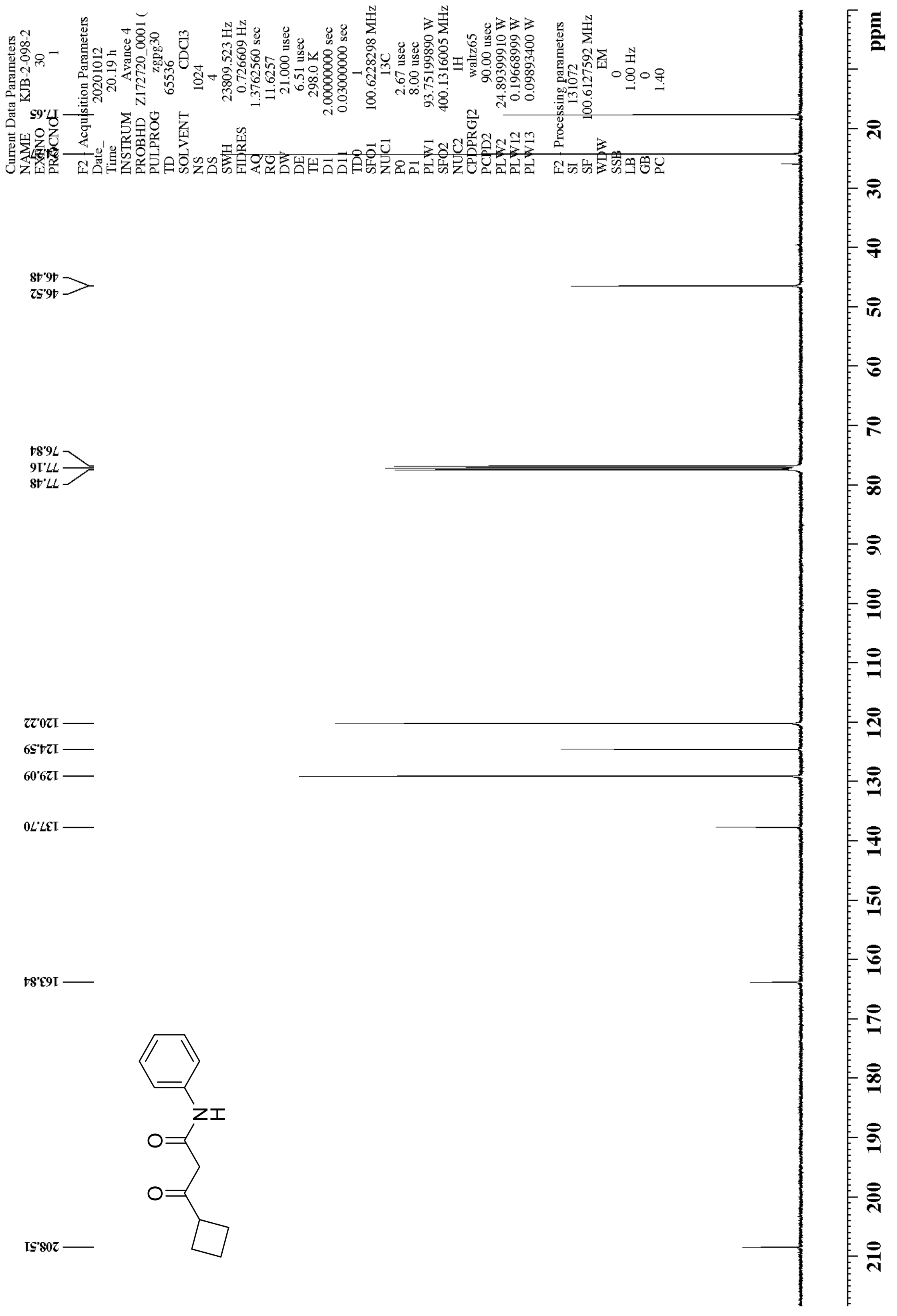

Supplementary Figure 20. ^{13}C NMR of β-oxo-*N*-phenylcyclobutanepropanamide (16).

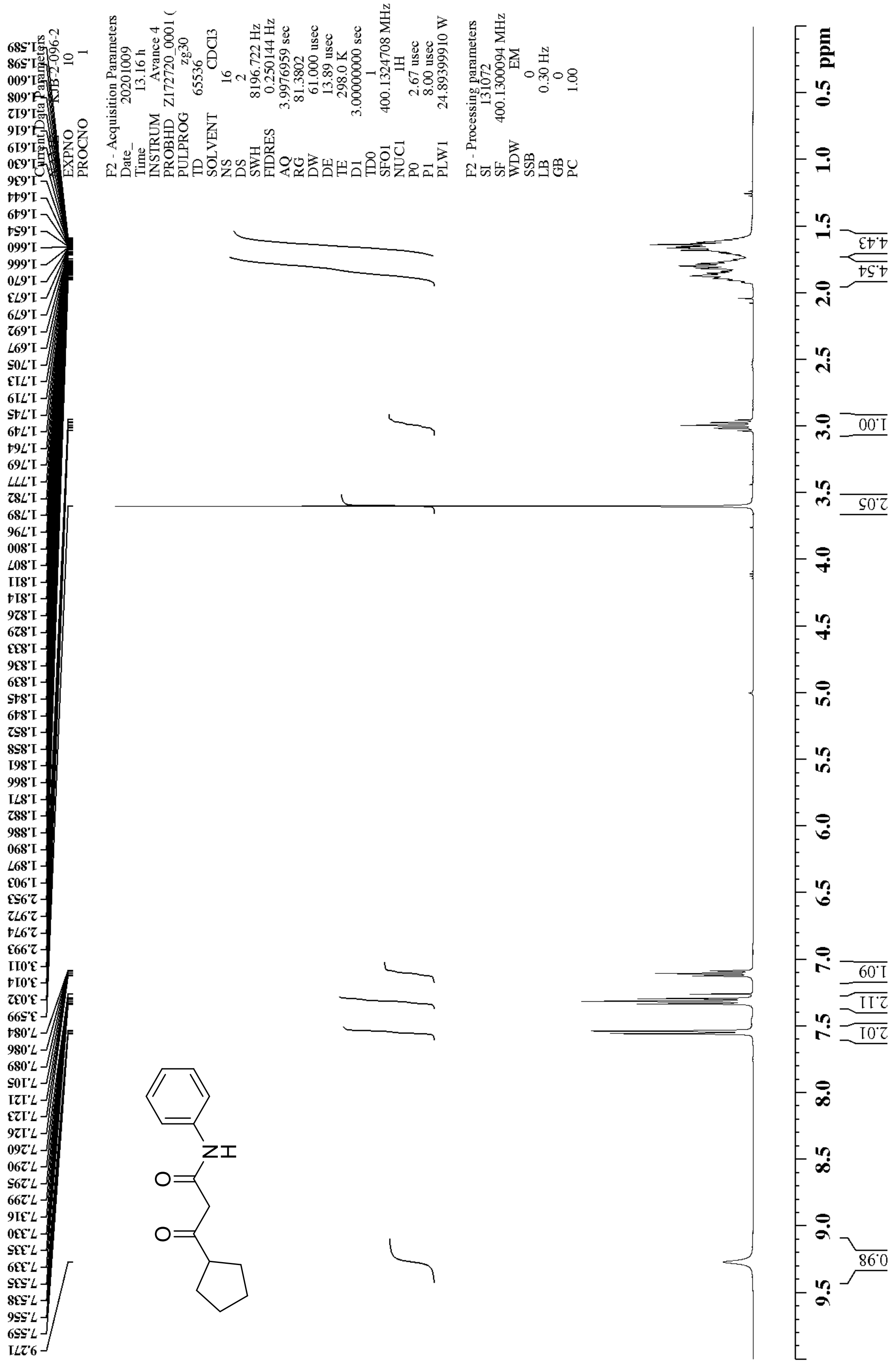

Supplementary Figure 21. ^{1}H NMR of β-oxo-*N*-phenylcyclopentanepropanamide (17).

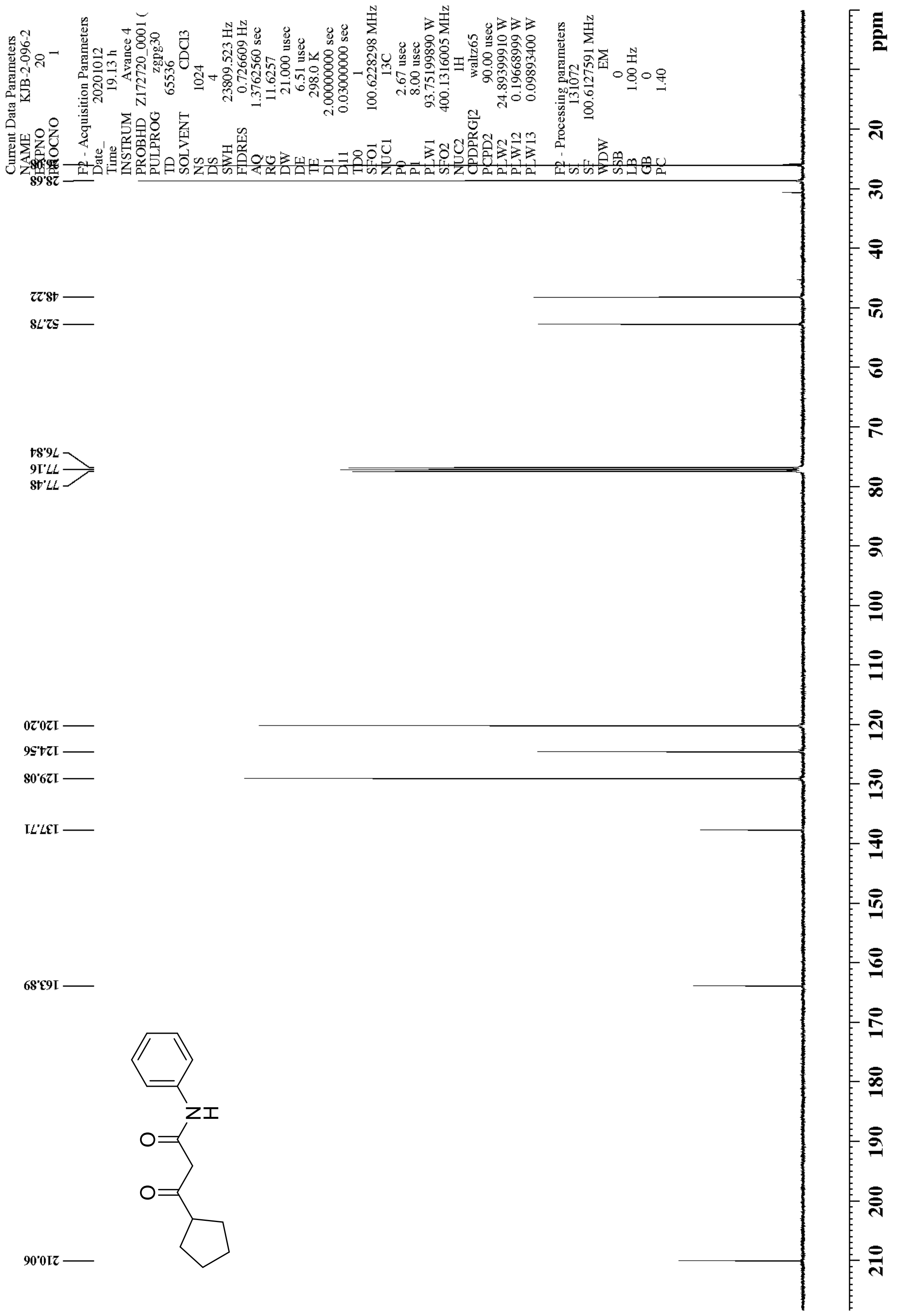

Supplementary Figure 22. **^{13}C NMR of β-oxo-*N*-phenylcyclopentanepropanamide (17).**

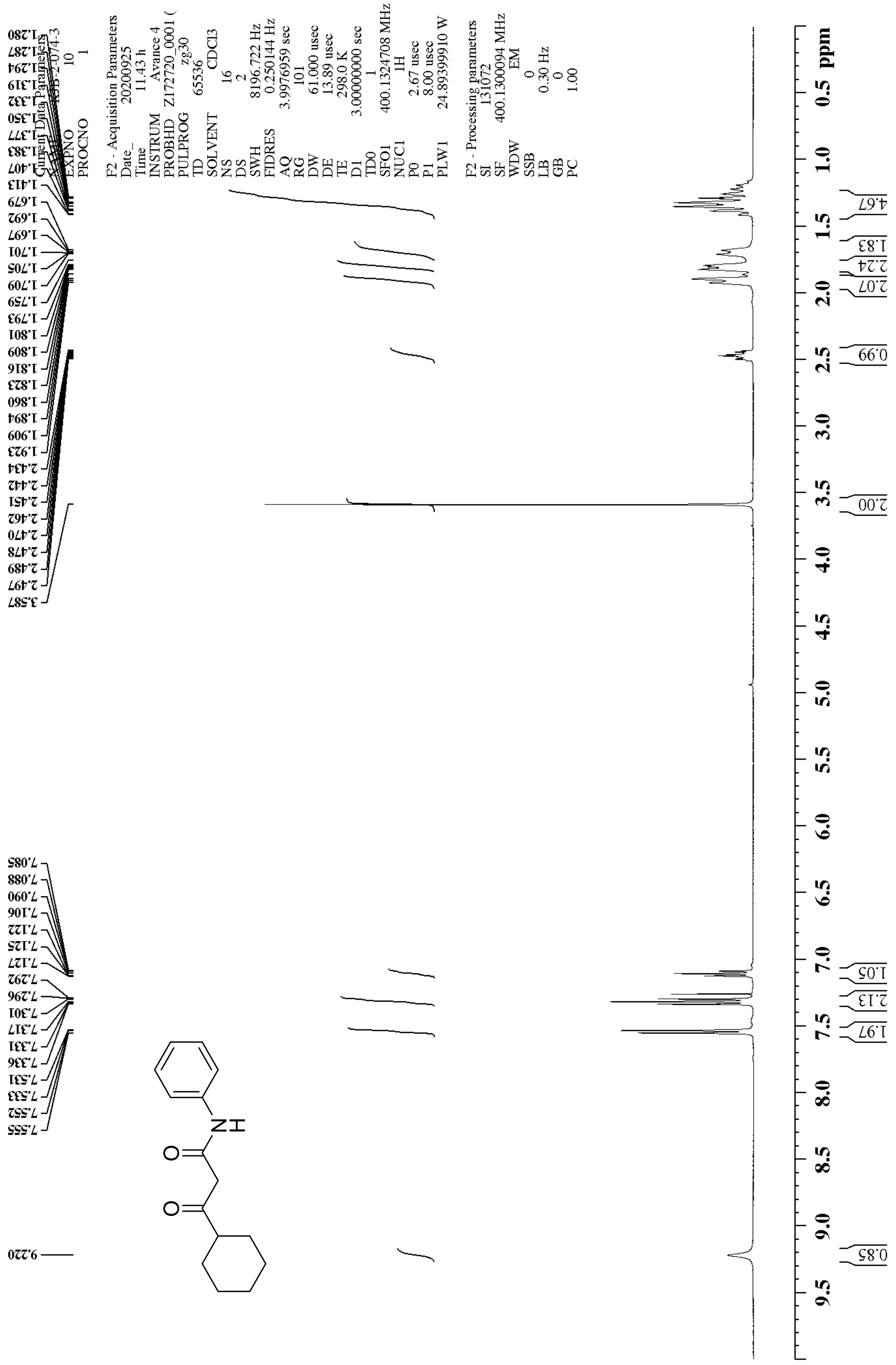

Supplementary Figure 23. ^{1}H NMR of β-oxo-*N*-phenylcyclohexanepropanamide (18).

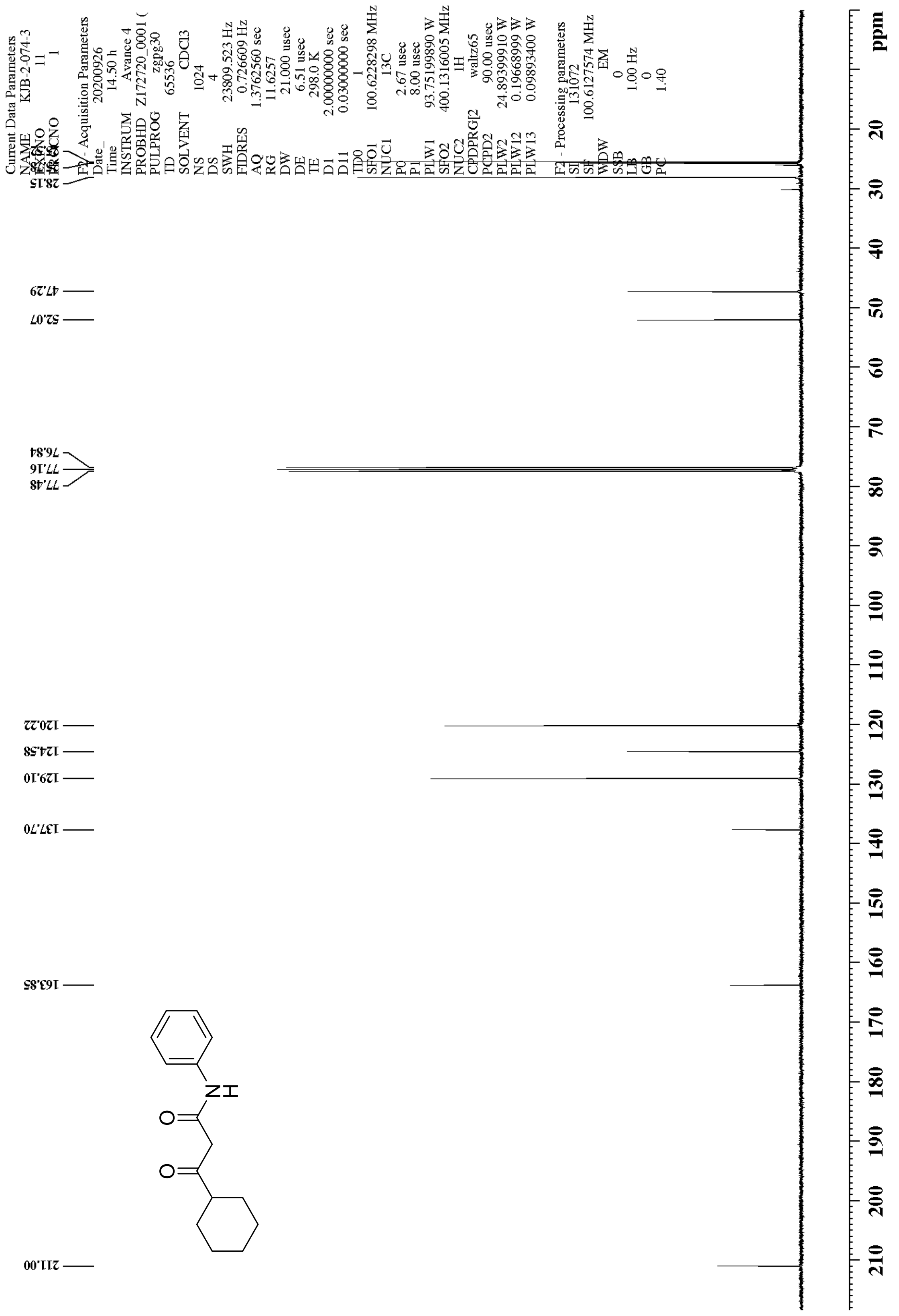

Supplementary Figure 24. ^{13}**C NMR of β-oxo-*N*-phenylcyclohexanepropanamide (18).**

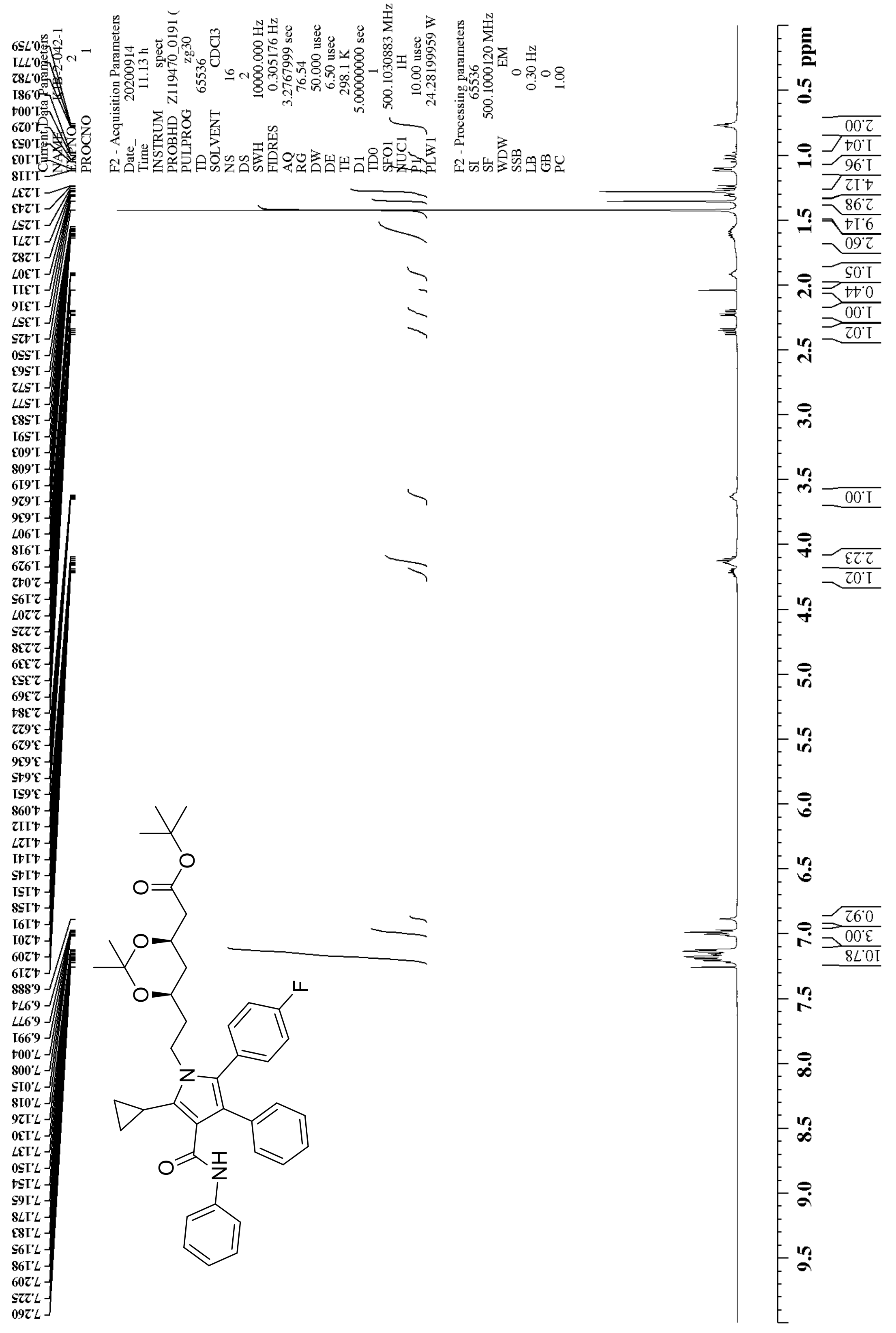

Supplementary Figure 25. ^{1}H NMR of 1,1-dimethylethyl (4*R*,6*R*)-6-[2-[5-cyclopropyl-2-(4-fluorophenyl)-3-phenyl-4-[(phenylamino)carbonyl]-1*H*-pyrrol-1-yl]ethyl]-2,2-dimethyl-1,3-dioxane-4-acetate (19).

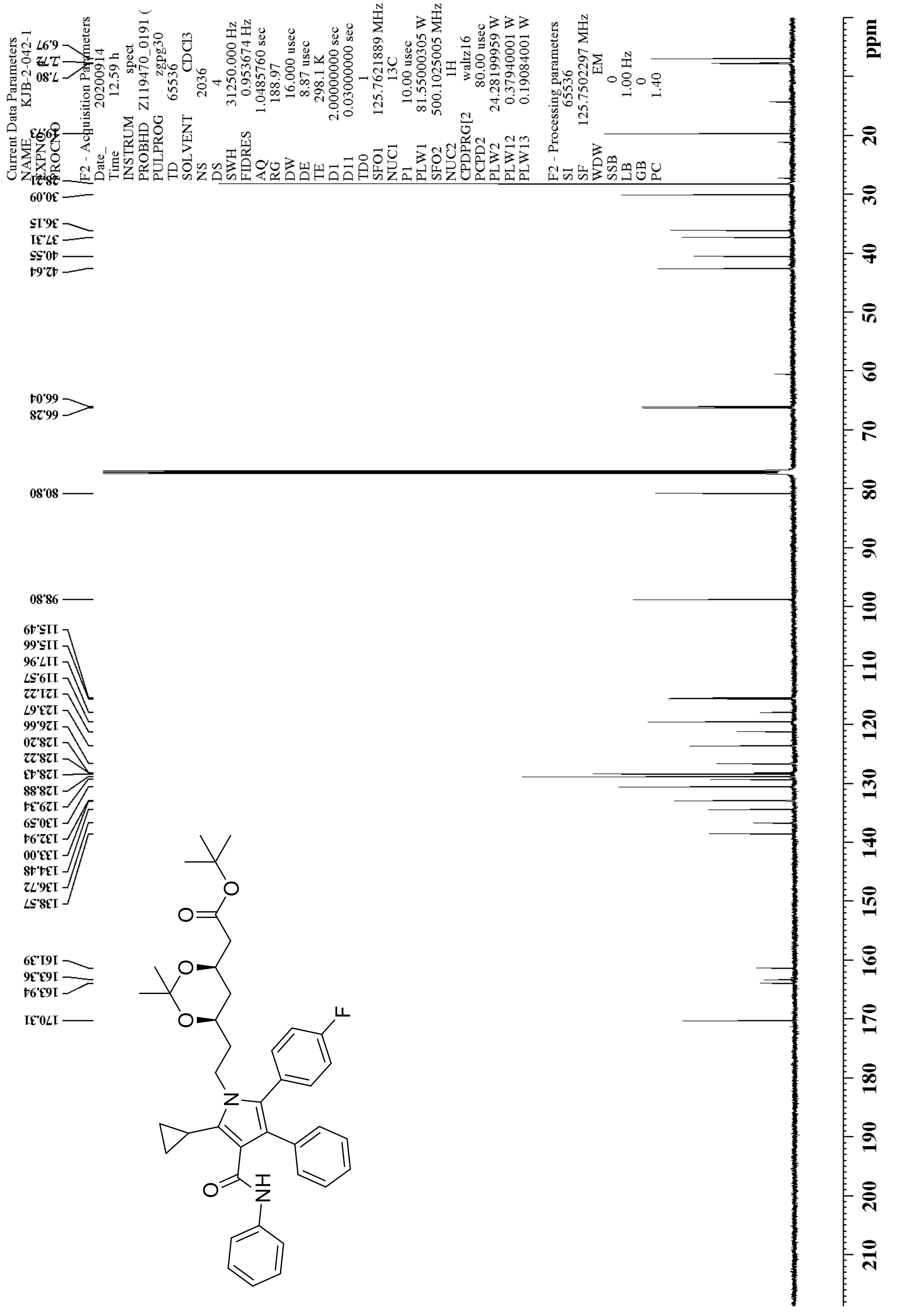

Supplementary Figure 26. ^{13}C **NMR of 1,1-dimethylethyl (4*R*,6*R*)-6-[2-[5-cyclopropyl-2-(4-fluorophenyl)-3-phenyl-4-[(phenylamino)carbonyl]-1*H*-pyrrol-1-yl]ethyl]-2,2-dimethyl-1,3-dioxane-4-acetate (19).**

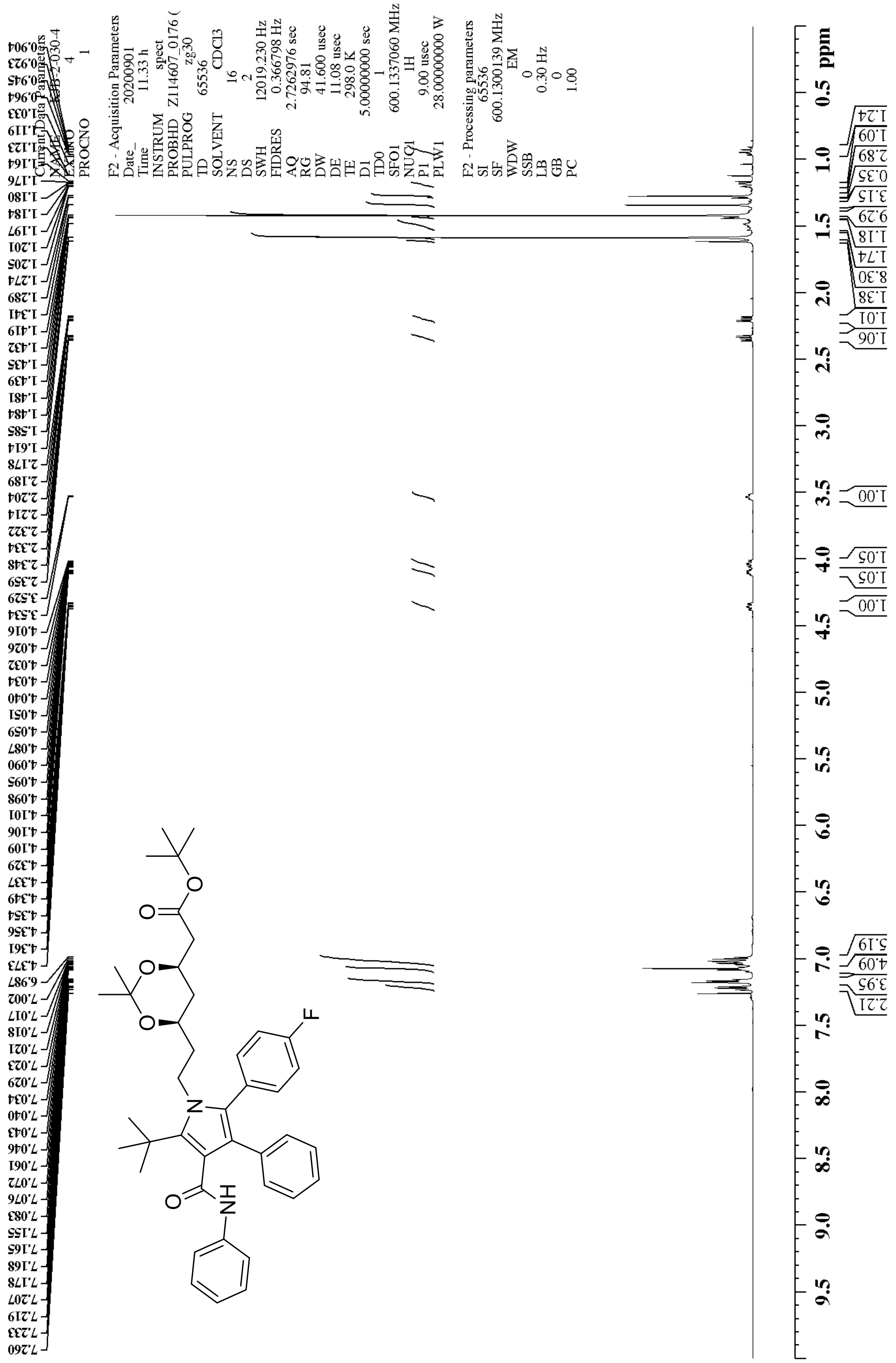

Supplementary Figure 27. ^{1}H NMR of 1,1-dimethylethyl (4*R*,6*R*)-6-[2-[2-(4-fluorophenyl)-5-(1,1-dimethylethyl)-3-phenyl-4-[(phenylamino)carbonyl]-1*H*-pyrrol-1-yl]ethyl]-2,2-dimethyl-1,3-dioxane-4-acetate (20).

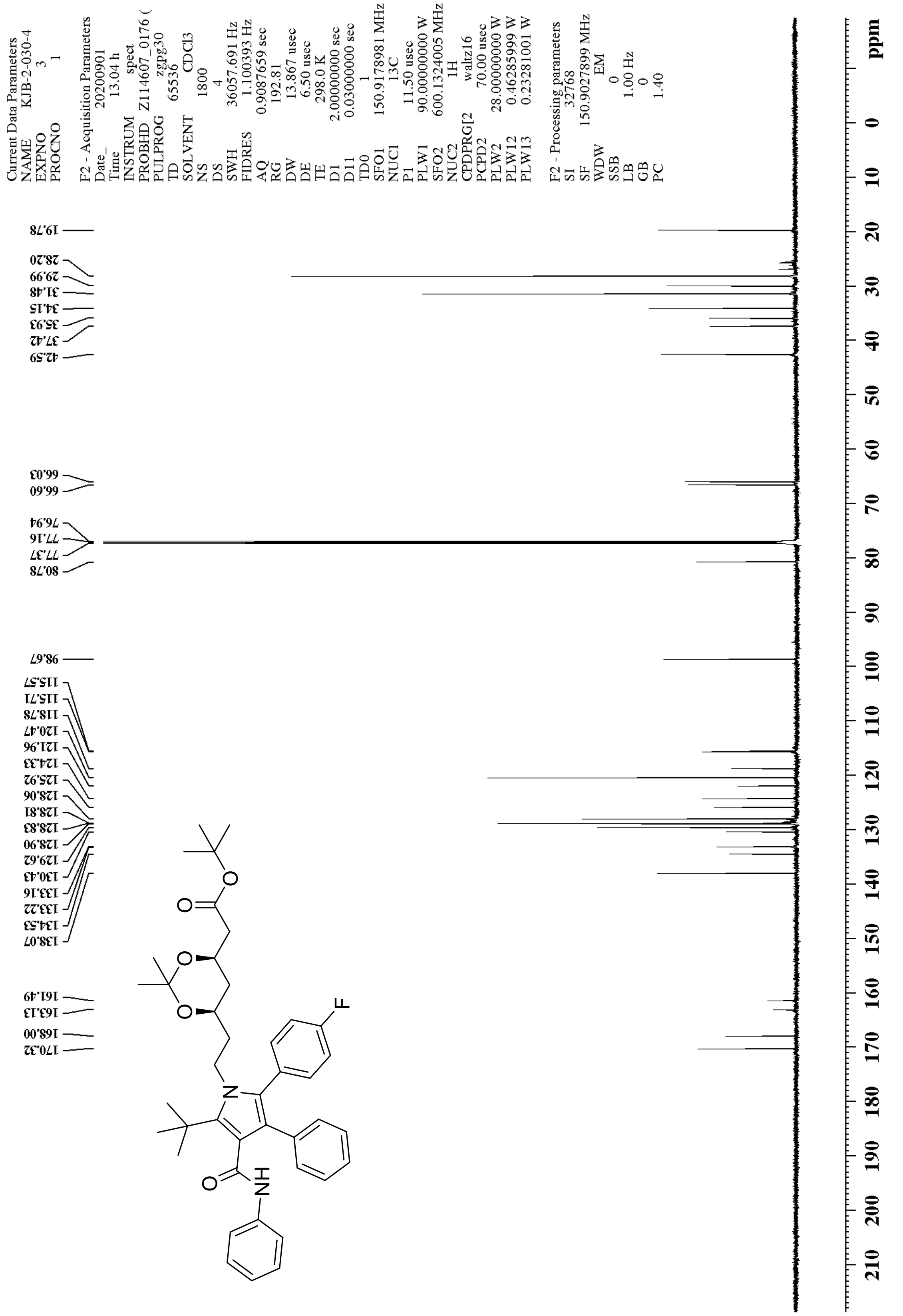

Supplementary Figure 28. ^{13}**C NMR of 1,1-dimethylethyl (4*R*,6*R*)-6-[2-[2-(4-fluorophenyl)-5-(1,1-dimethylethyl)-3-phenyl-4-[(phenylamino)carbonyl]-1*H*-pyrrol-1-yl]ethyl]-2,2-dimethyl-1,3-dioxane-4-acetate (20).**

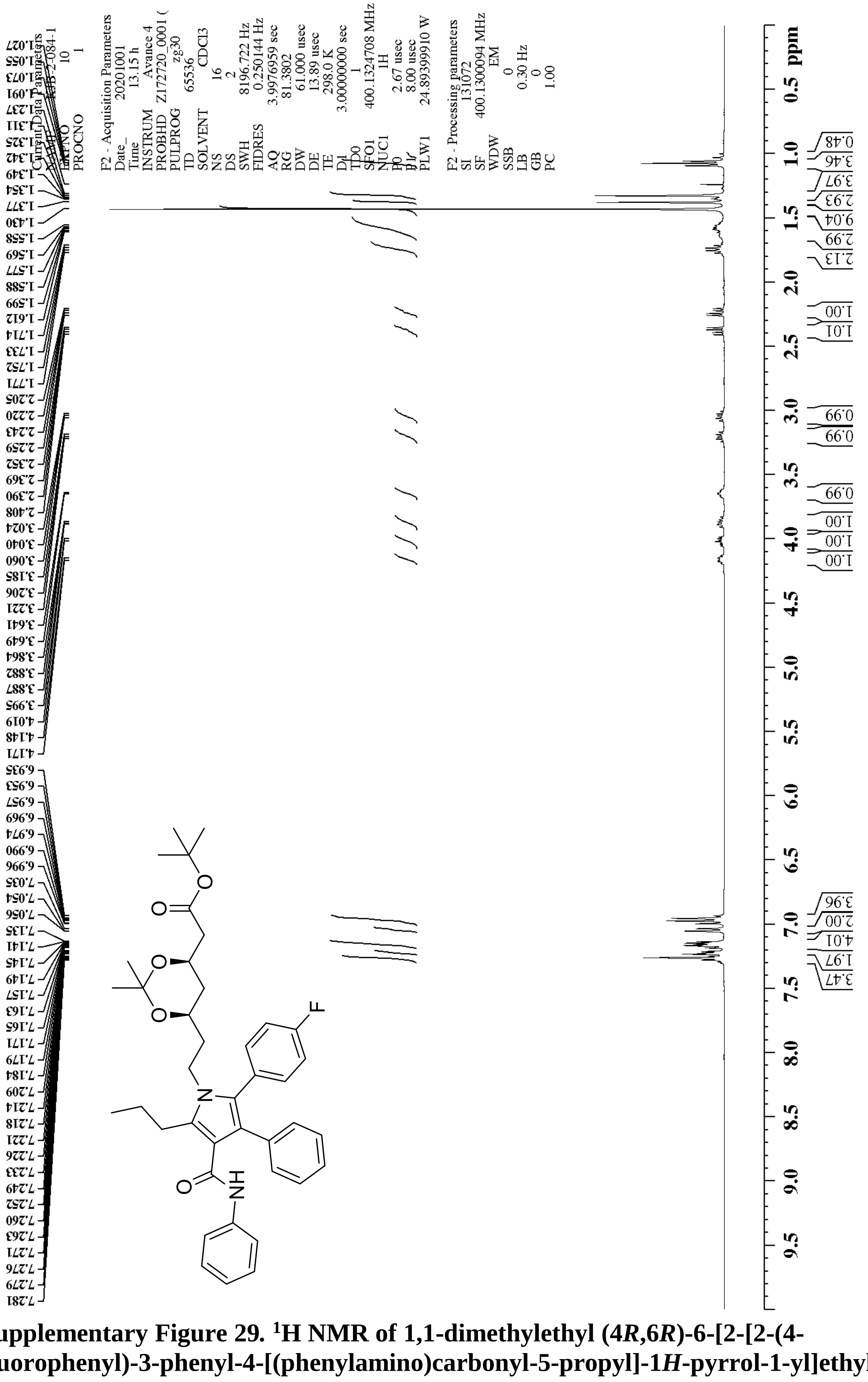

Supplementary Figure 29. ^{1}H NMR of 1,1-dimethylethyl (4*R*,6*R*)-6-[2-[2-(4-fluorophenyl)-3-phenyl-4-[(phenylamino)carbonyl-5-propyl]-1*H*-pyrrol-1-yl]ethyl]-2,2-dimethyl-1,3-dioxane-4-acetate (21).

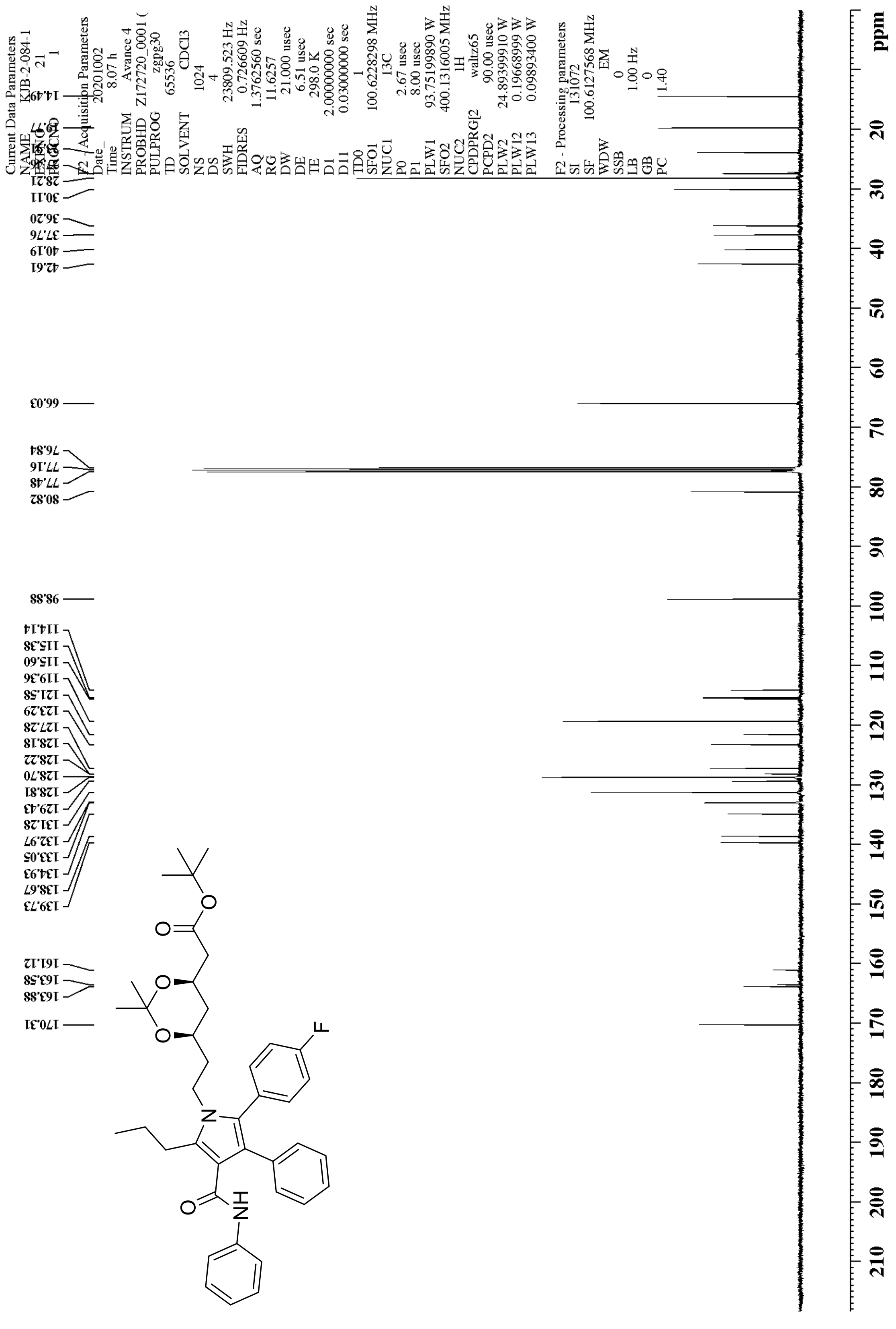

Supplementary Figure 30. **^{13}C NMR of 1,1-dimethylethyl (4*R*,6*R*)-6-[2-[2-(4-fluorophenyl)-3-phenyl-4-[(phenylamino)carbonyl-5-propyl]-1*H*-pyrrol-1-yl]ethyl]-2,2-dimethyl-1,3-dioxane-4-acetate (21).**

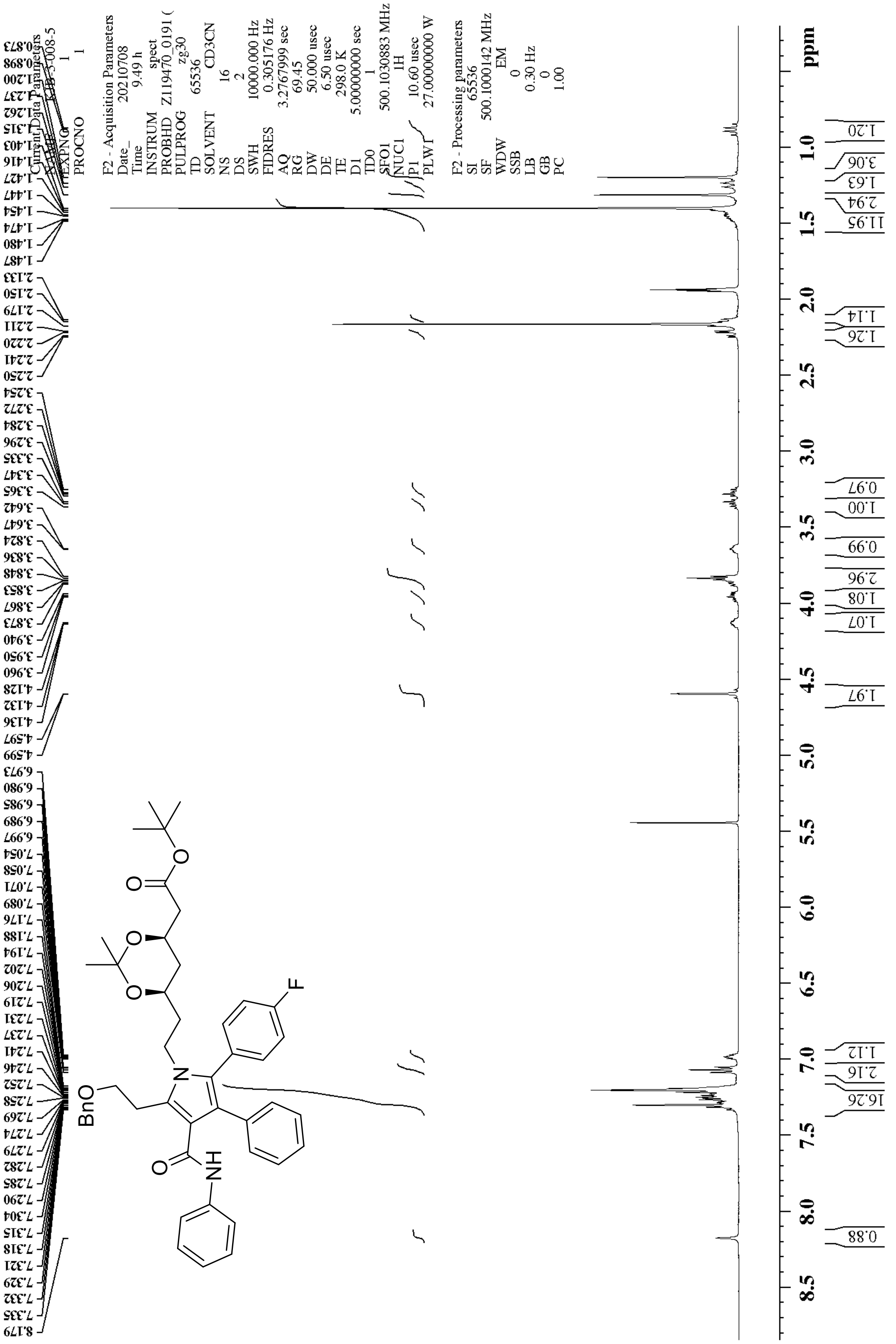

Supplementary Figure 31. ^{1}H NMR of 1,1-dimethylethyl (4*R*,6*R*)-6-[5-(2-benzyloxyethyl)-2-[2-(4-fluorophenyl)-3-phenyl-4-[(phenylamino)carbonyl]-1*H*-pyrrol-1-yl]ethyl]-2,2-dimethyl-1,3-dioxane-4-acetate (22).

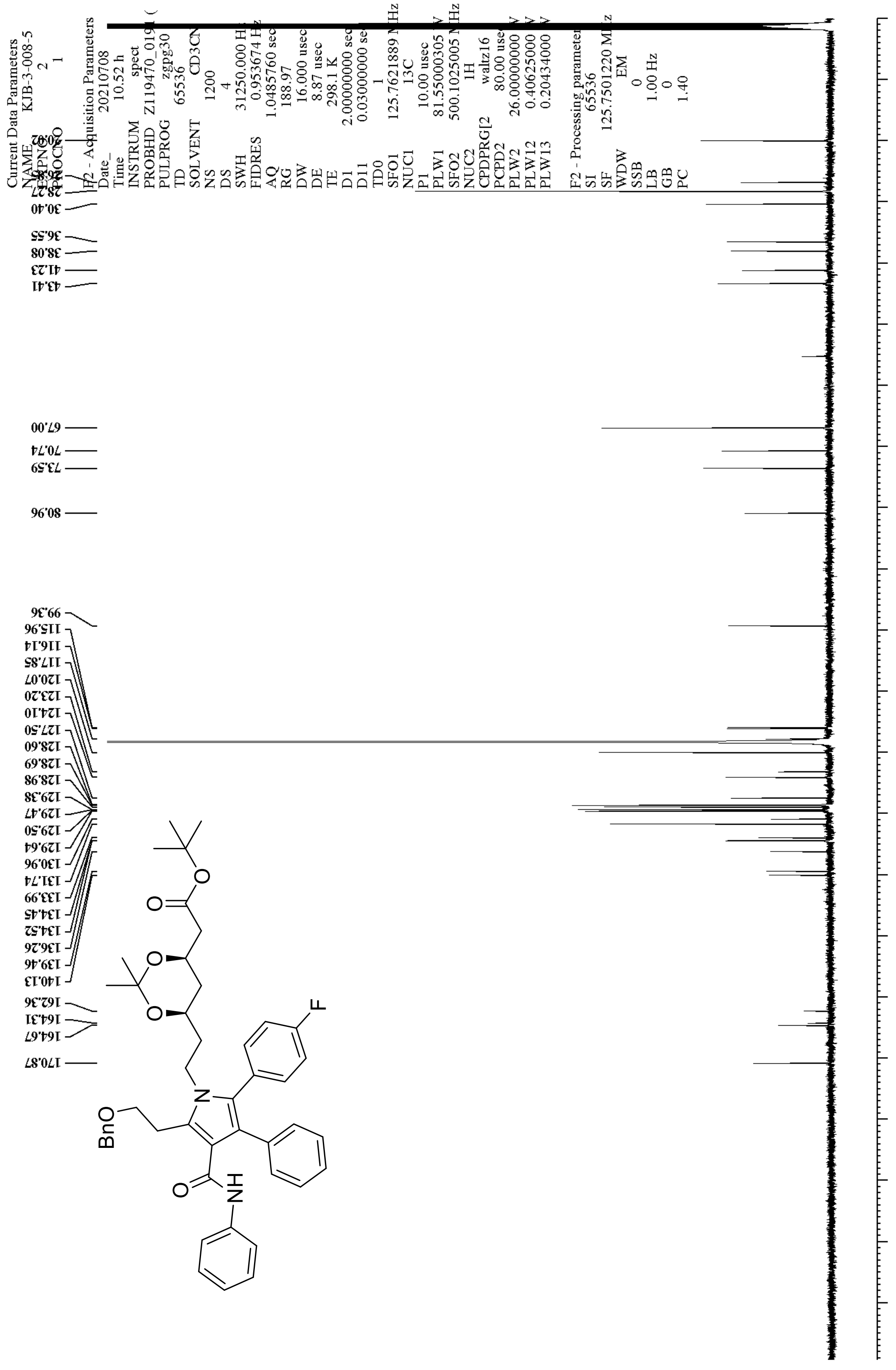

Supplementary Figure 32. ^{13}**C NMR of 1,1-dimethylethyl (4*R*,6*R*)-6-[5-(2-benzyloxyethyl)-2-[2-(4-fluorophenyl)-3-phenyl-4-[(phenylamino)carbonyl]-1*H*-pyrrol-1-yl]ethyl]-2,2-dimethyl-1,3-dioxane-4-acetate (22).**

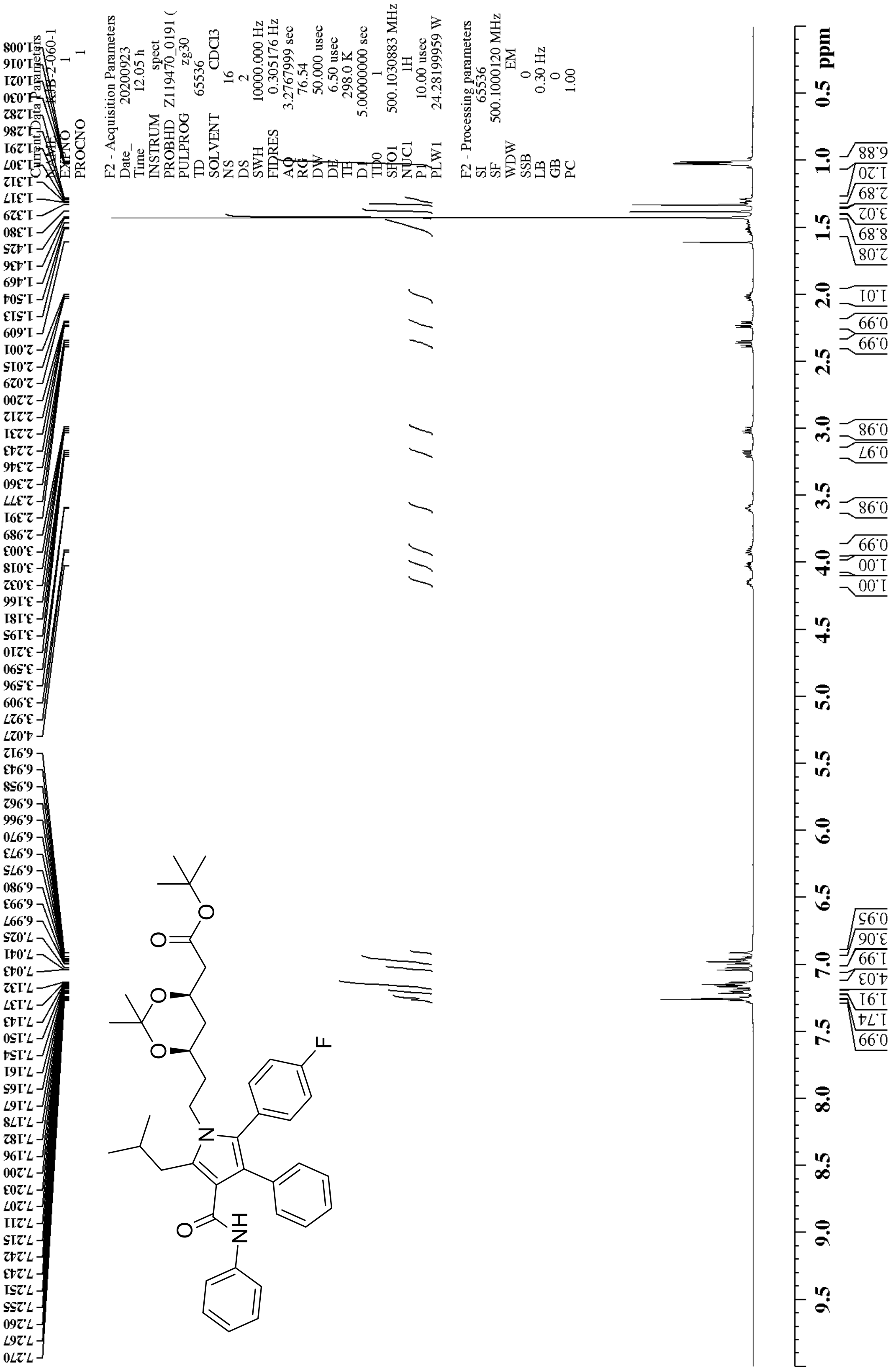

Supplementary Figure 33. ^{1}H NMR of 1,1-dimethylethyl (4*R*,6*R*)-6-[2-[2-(4-fluorophenyl)-5-(2-methylpropyl)-3-phenyl-4-[(phenylamino)carbonyl]-1*H*-pyrrol-1-yl]ethyl]-2,2-dimethyl-1,3-dioxane-4-acetate (23).

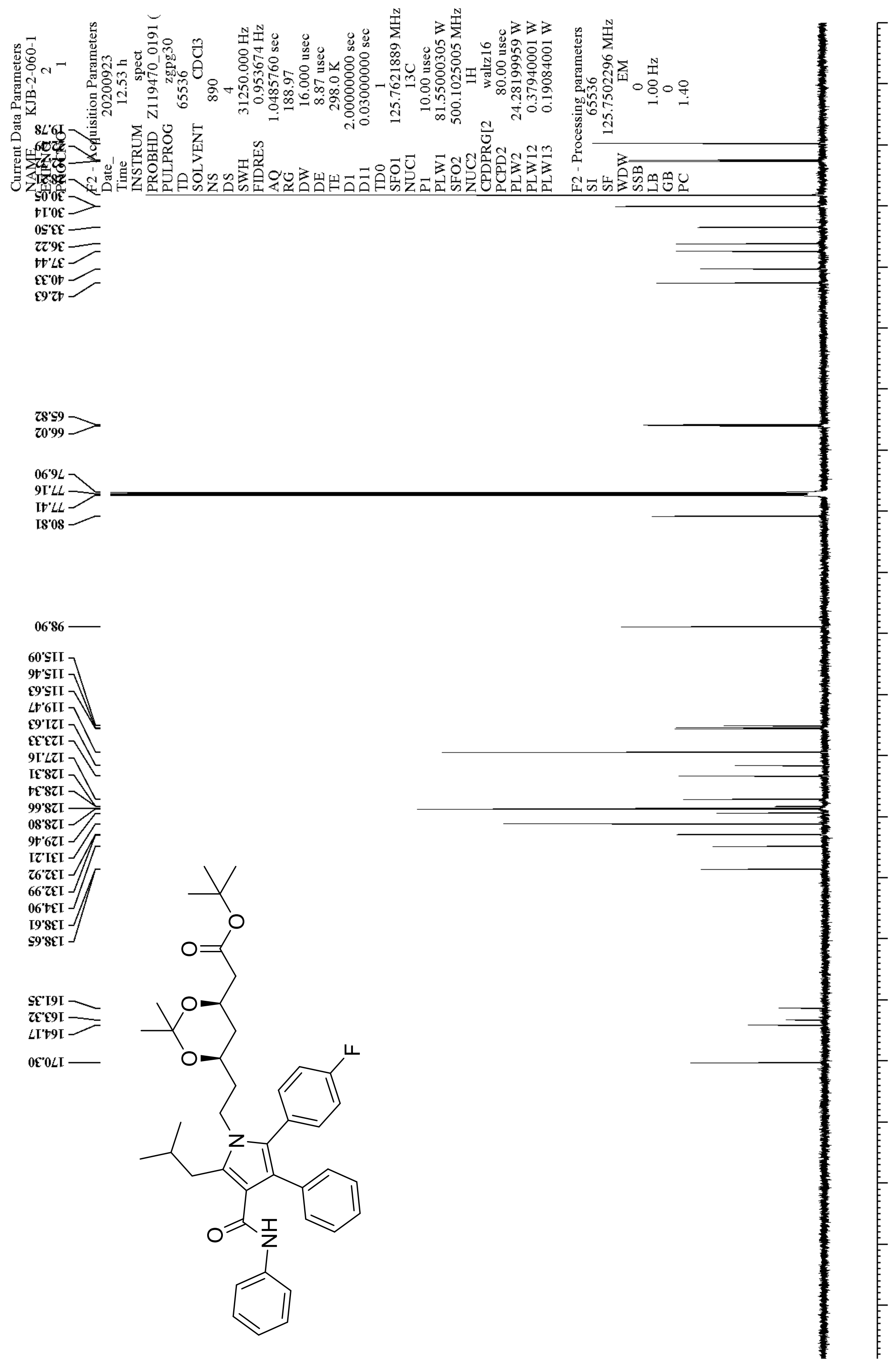

Supplementary Figure 34. ^{13}**C NMR of 1,1-dimethylethyl (4*R*,6*R*)-6-[2-[2-(4-fluorophenyl)-5-(2-methylpropyl)-3-phenyl-4-[(phenylamino)carbonyl]-1*H*-pyrrol-1-yl]ethyl]-2,2-dimethyl-1,3-dioxane-4-acetate (23).**

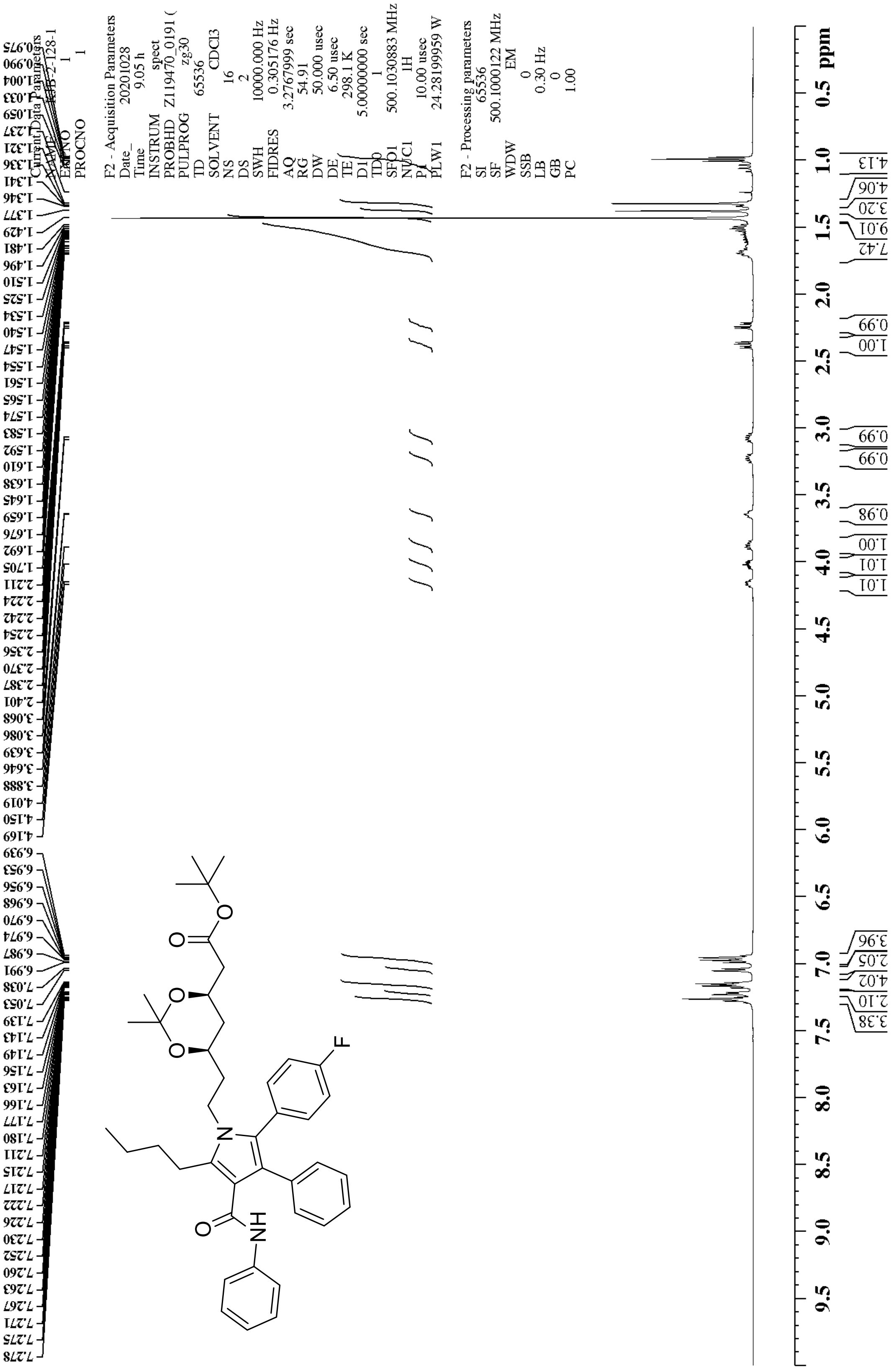

Supplementary Figure 35. ^{1}H NMR of 1,1-dimethylethyl (4*R*,6*R*)-6-[2-[5-butyl-2-(4-fluorophenyl)-3-phenyl-4-[(phenylamino)carbonyl]-1*H*-pyrrol-1-yl]ethyl]-2,2-dimethyl-1,3-dioxane-4-acetate (24).

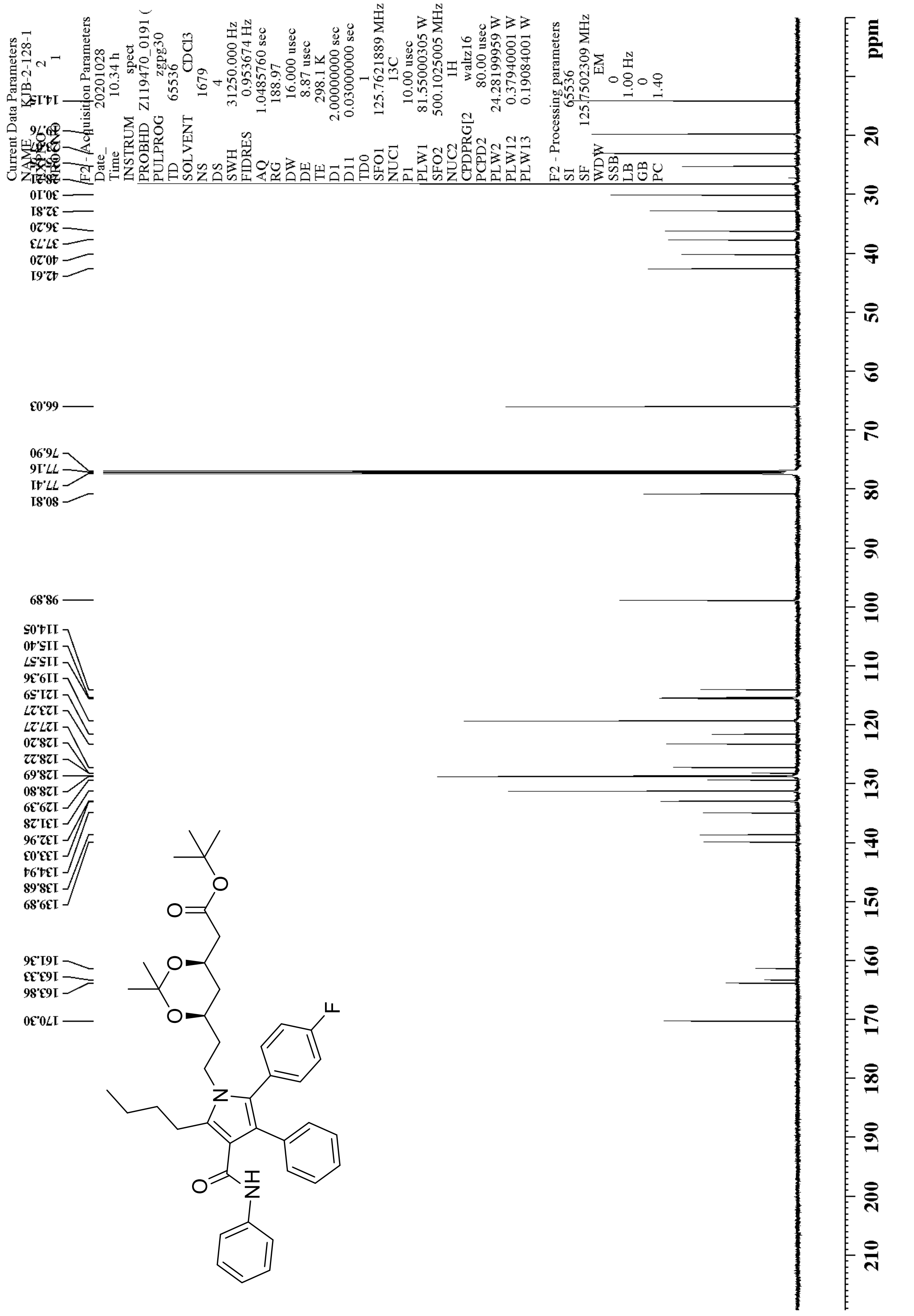

Supplementary Figure 36. ^{13}C NMR of 1,1-dimethylethyl (4*R*,6*R*)-6-[2-[5-butyl-2-(4-fluorophenyl)-3-phenyl-4-[(phenylamino)carbonyl]-1*H*-pyrrol-1-yl]ethyl]-2,2-dimethyl-1,3-dioxane-4-acetate (24).

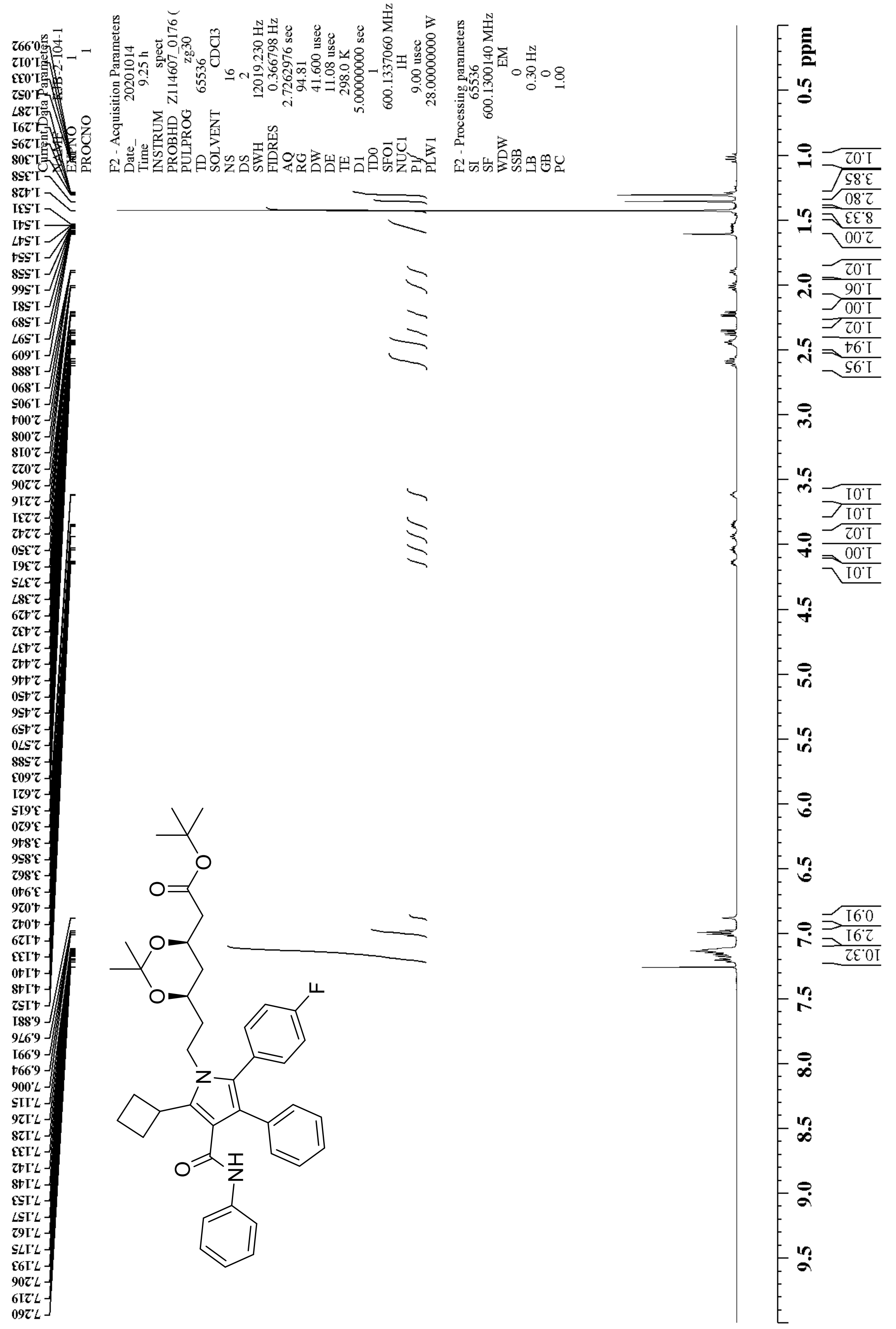

Supplementary Figure 37. ^{1}H NMR of 1,1-dimethylethyl (4*R*,6*R*)-6-[2-[5-cyclobutyl-2-(4-fluorophenyl)-3-phenyl-4-[(phenylamino)carbonyl]-1*H*-pyrrol-1-yl]ethyl]-2,2-dimethyl-1,3-dioxane-4-acetate (25).

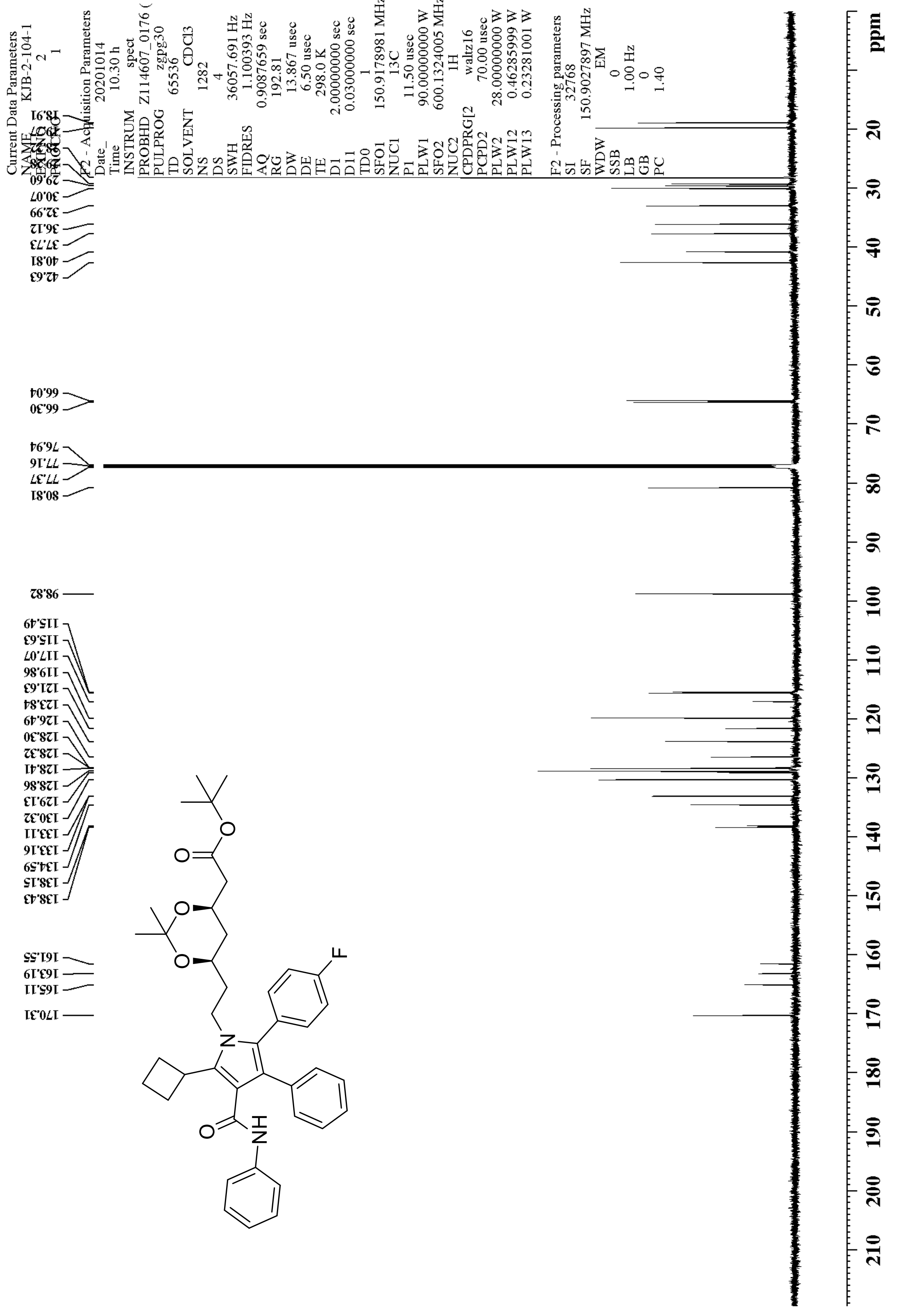

Supplementary Figure 38. ^{13}C **NMR of 1,1-dimethylethyl (4*R*,6*R*)-6-[2-[5-cyclobutyl-2-(4-fluorophenyl)-3-phenyl-4-[(phenylamino)carbonyl]-1*H*-pyrrol-1-yl]ethyl]-2,2-dimethyl-1,3-dioxane-4-acetate (25).**

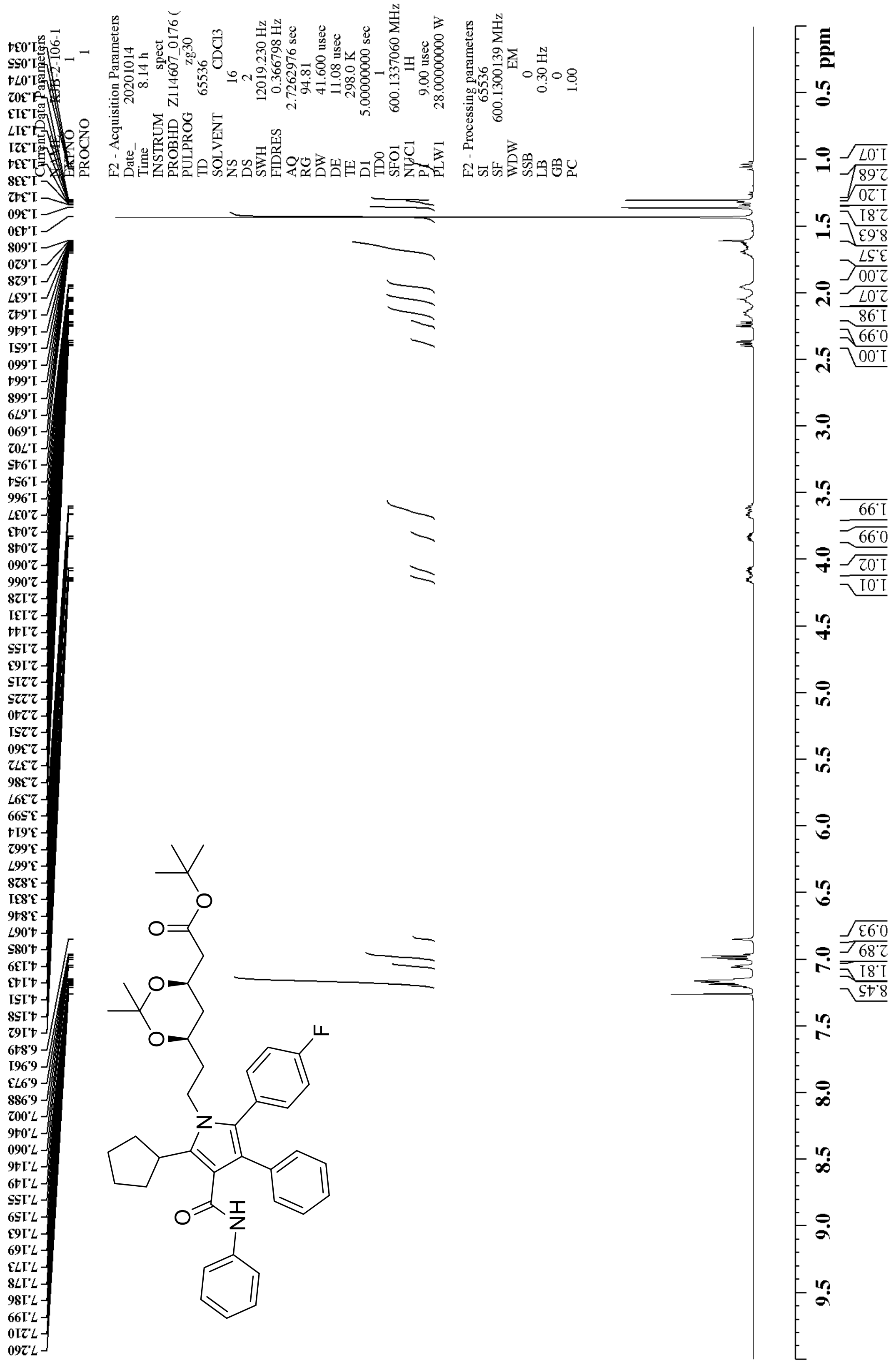

Supplementary Figure 39. ^{1}H NMR of 1,1-dimethylethyl (4*R*,6*R*)-6-[2-[5-cyclopentyl-2-(4-fluorophenyl)-3-phenyl-4-[(phenylamino)carbonyl]-1*H*-pyrrol-1-yl]ethyl]-2,2-dimethyl-1,3-dioxane-4-acetate (26).

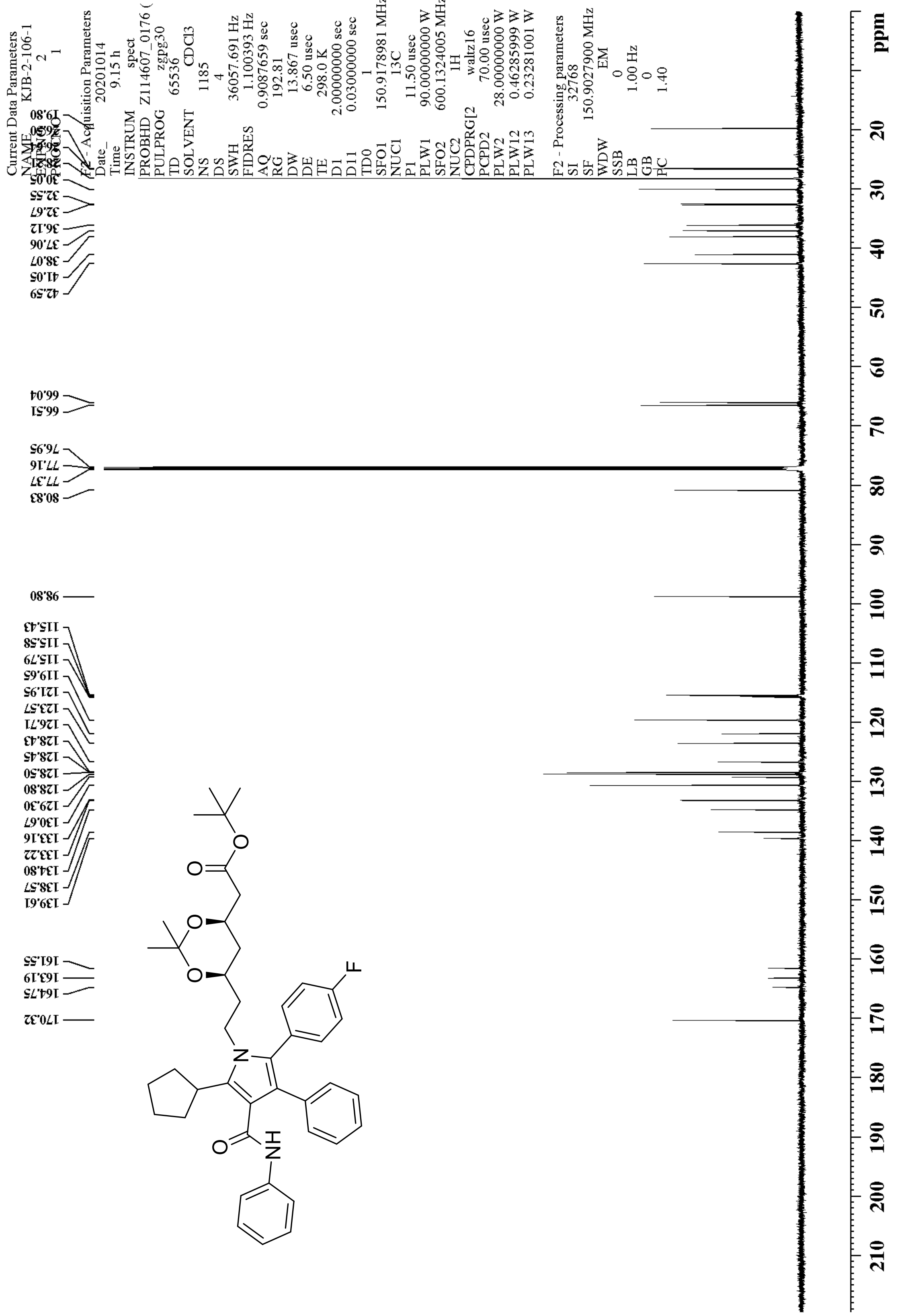

Supplementary Figure 40. ^{13}**C NMR of 1,1-dimethylethyl (4*R*,6*R*)-6-[2-[5-cyclopentyl-2-(4-fluorophenyl)-3-phenyl-4-[(phenylamino)carbonyl]-1*H*-pyrrol-1-yl]ethyl]-2,2-dimethyl-1,3-dioxane-4-acetate (26).**

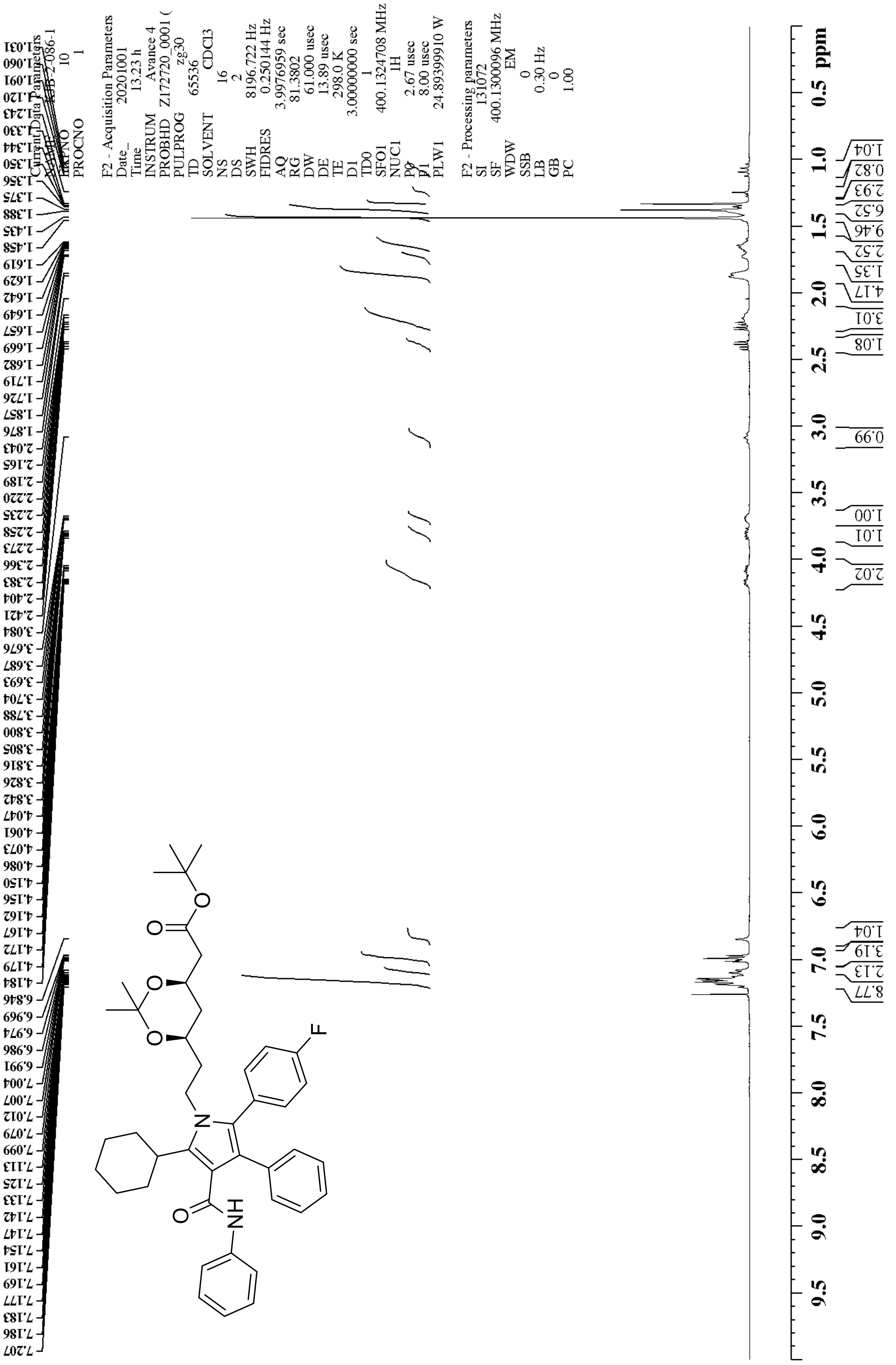

Supplementary Figure 41. **^{1}H NMR of 1,1-dimethylethyl (4*R*,6*R*)-6-[2-[5-cyclohexyl-2-(4-fluorophenyl)-3-phenyl-4-[(phenylamino)carbonyl]-1*H*-pyrrol-1-yl]ethyl]-2,2-dimethyl-1,3-dioxane-4-acetate (27).**

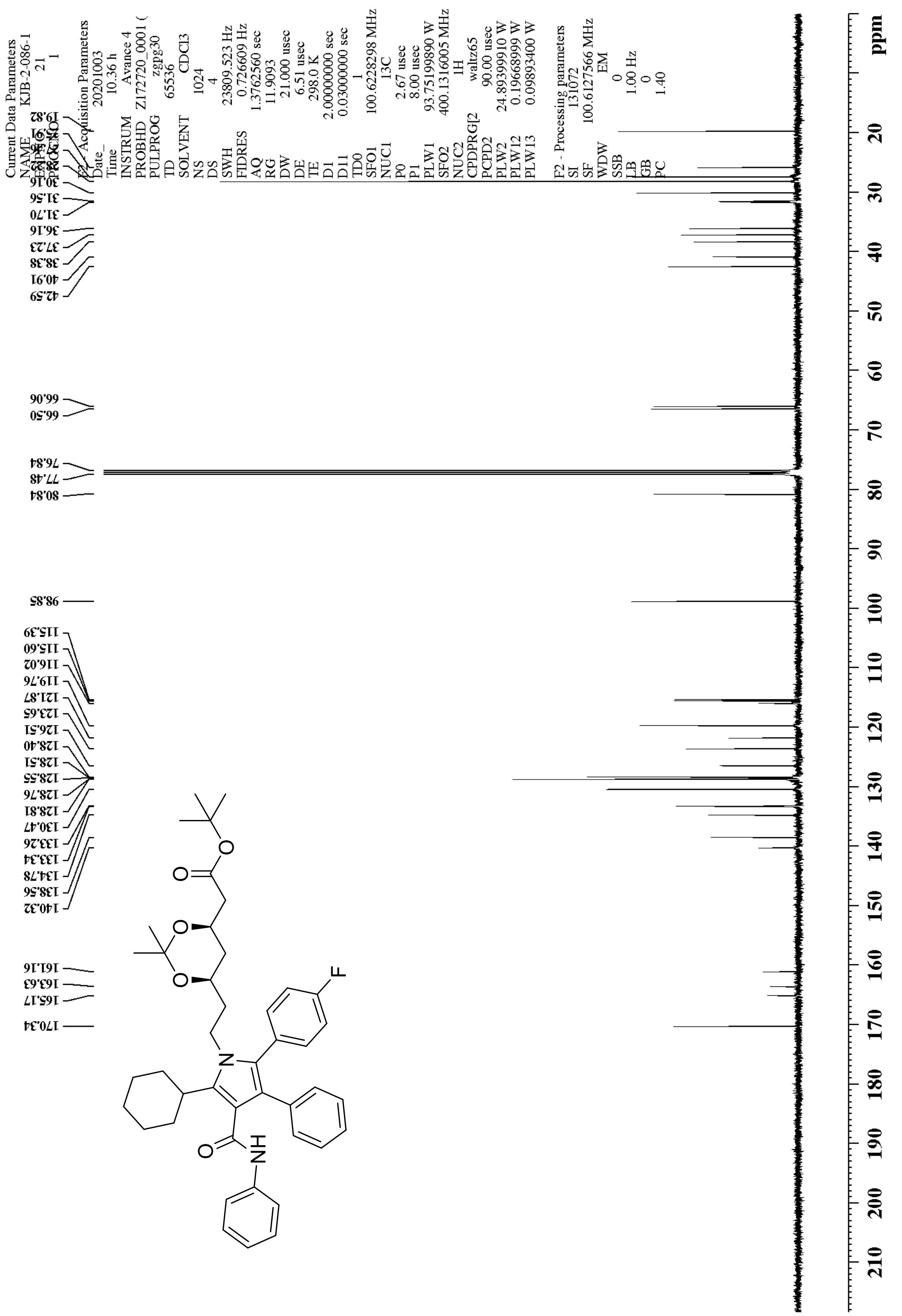

Supplementary Figure 42. ^{13}**C NMR of 1,1-dimethylethyl (4*R*,6*R*)-6-[2-[5-cyclohexyl-2-(4-fluorophenyl)-3-phenyl-4-[(phenylamino)carbonyl]-1*H*-pyrrol-1-yl]ethyl]-2,2-dimethyl-1,3-dioxane-4-acetate (27).**

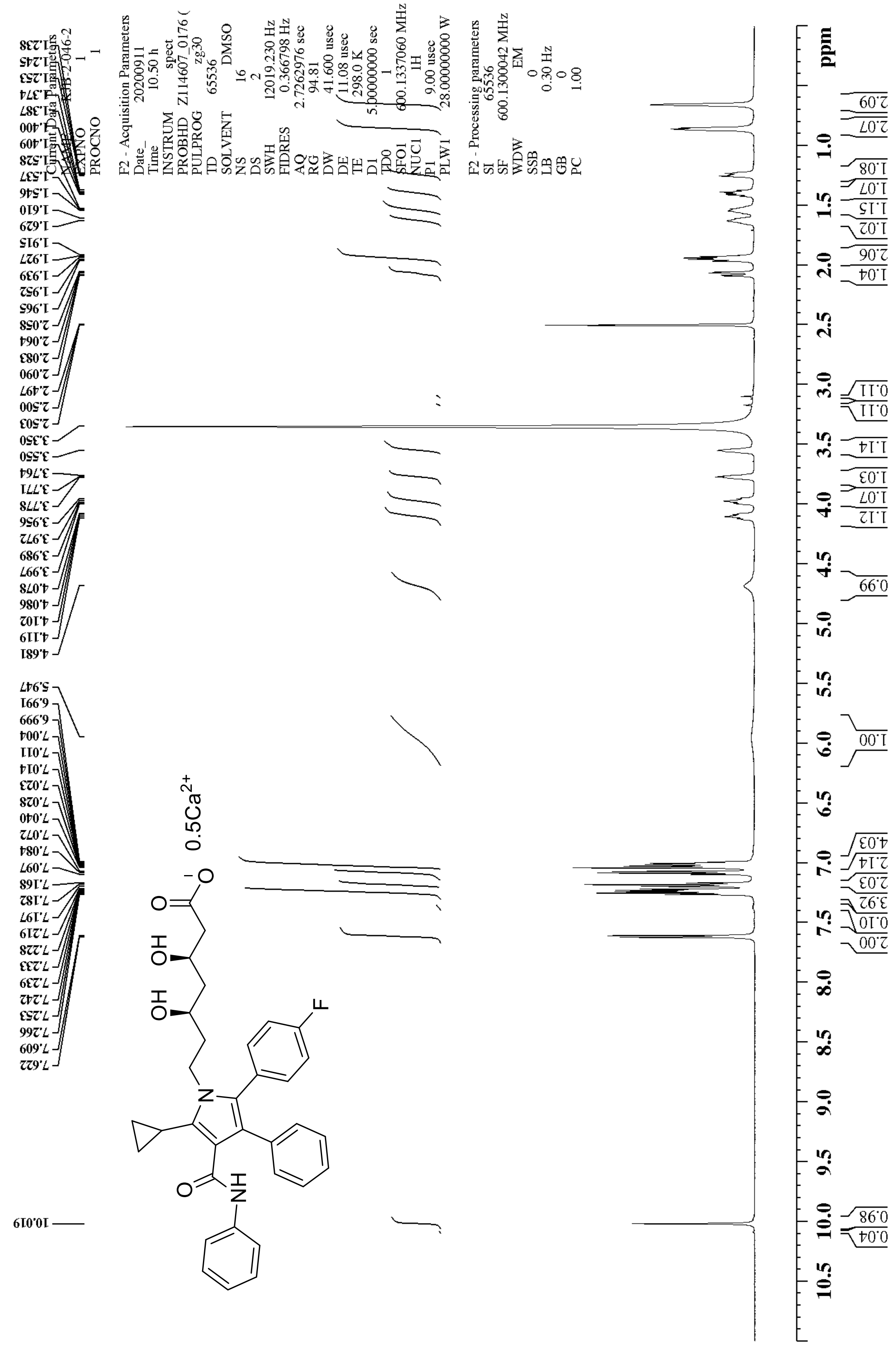

Supplementary Figure 43. ^{1}**H NMR of (β*R*,δ*R*)-5-cyclopropyl-2-(4-fluorophenyl)-β,δ-dihydroxy-3-phenyl-4-[(phenylamino)carbonyl]-1*H*-pyrrole-1-heptanoic acid hemicalcium salt (1).**

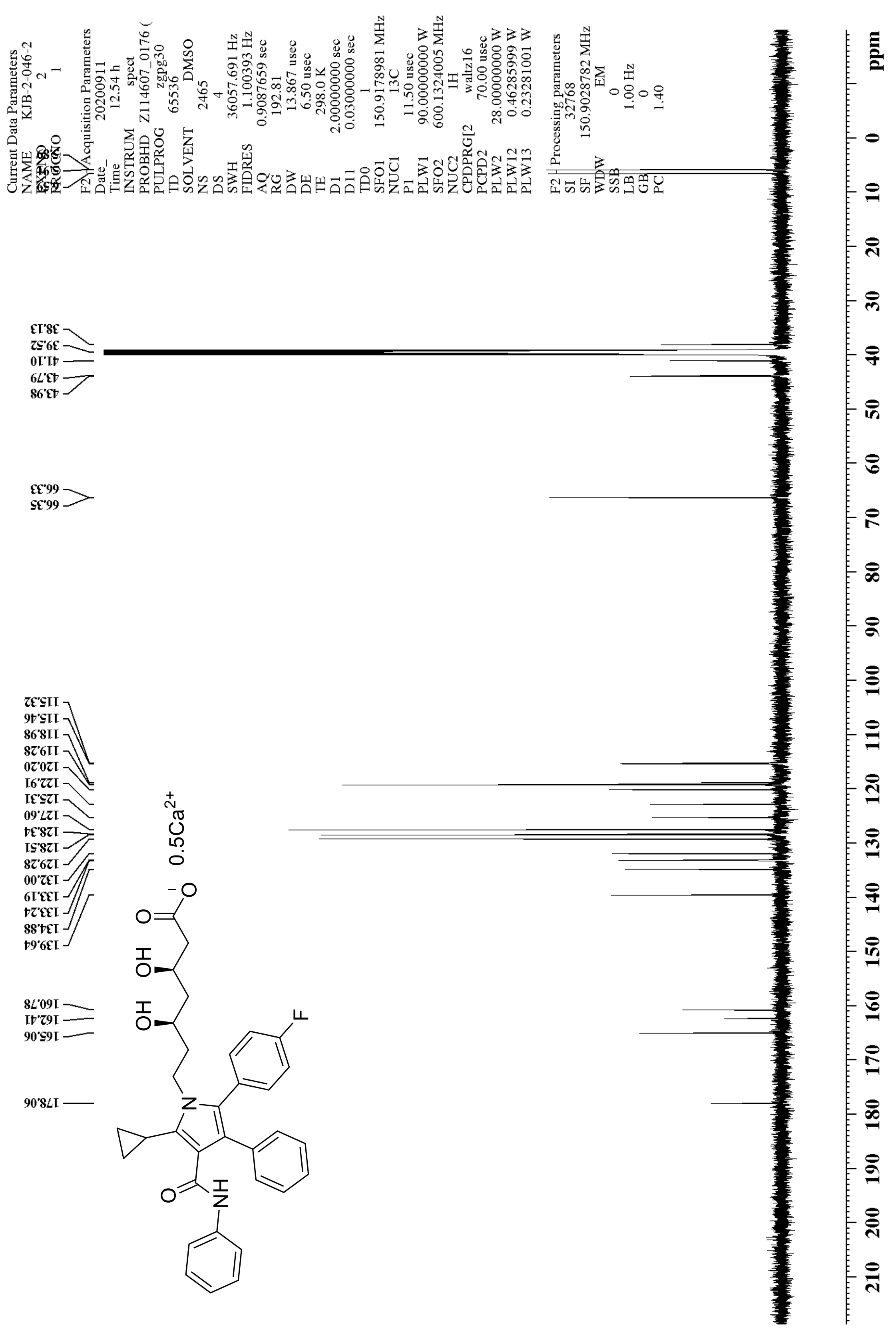

Supplementary Figure 44. ^{13}C **NMR of (β*R*,δ*R*)-5-cyclopropyl-2-(4-fluorophenyl)-β,δ-dihydroxy-3-phenyl-4-[(phenylamino)carbonyl]-1*H*-pyrrole-1-heptanoic acid hemicalcium salt (1).**

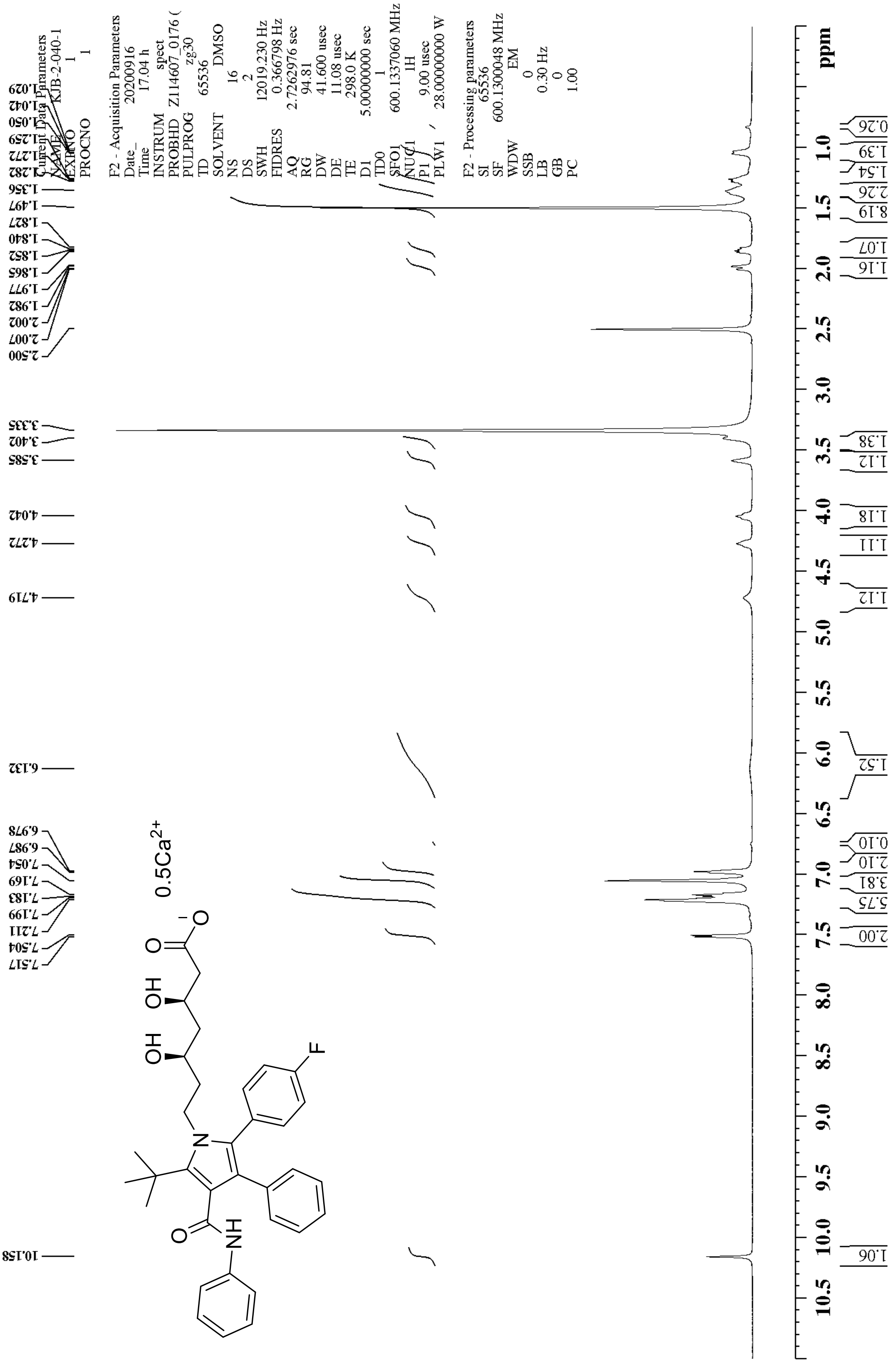

Supplementary Figure 45. **^{1}H NMR of (β*R*,δ*R*)-2-(4-fluorophenyl)-β,δ-dihydroxy-5-(1,1-dimethylethyl)-3-phenyl-4-[(phenylamino)carbonyl]-1*H*-pyrrole-1-heptanoic acid hemicalcium salt (2).**

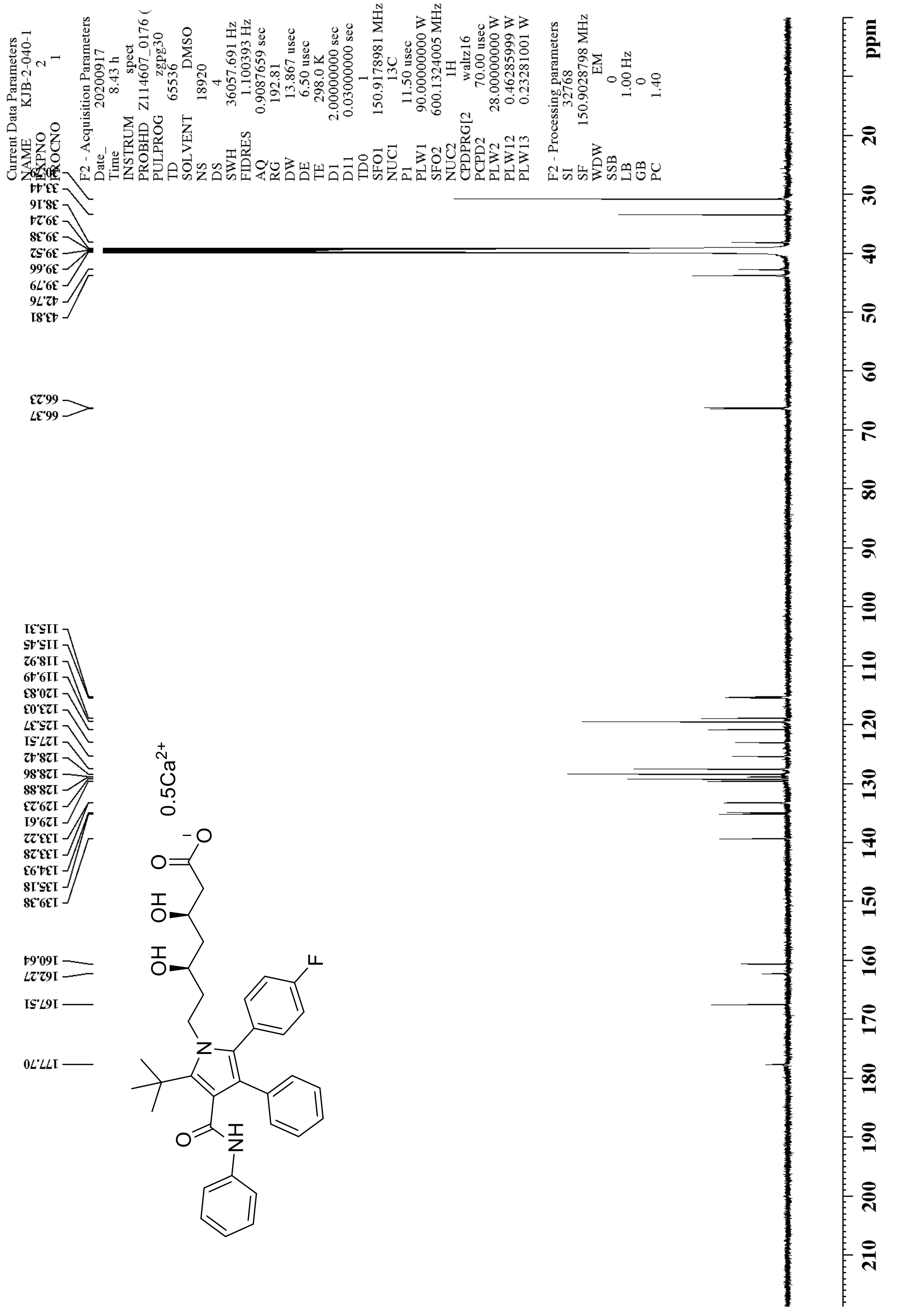

Supplementary Figure 46. ^{13}**C NMR of (β*R*,δ*R*)-2-(4-fluorophenyl)-β,δ-dihydroxy-5-(1,1-dimethylethyl)-3-phenyl-4-[(phenylamino)carbonyl]-1*H*-pyrrole-1-heptanoic acid hemicalcium salt (2).**

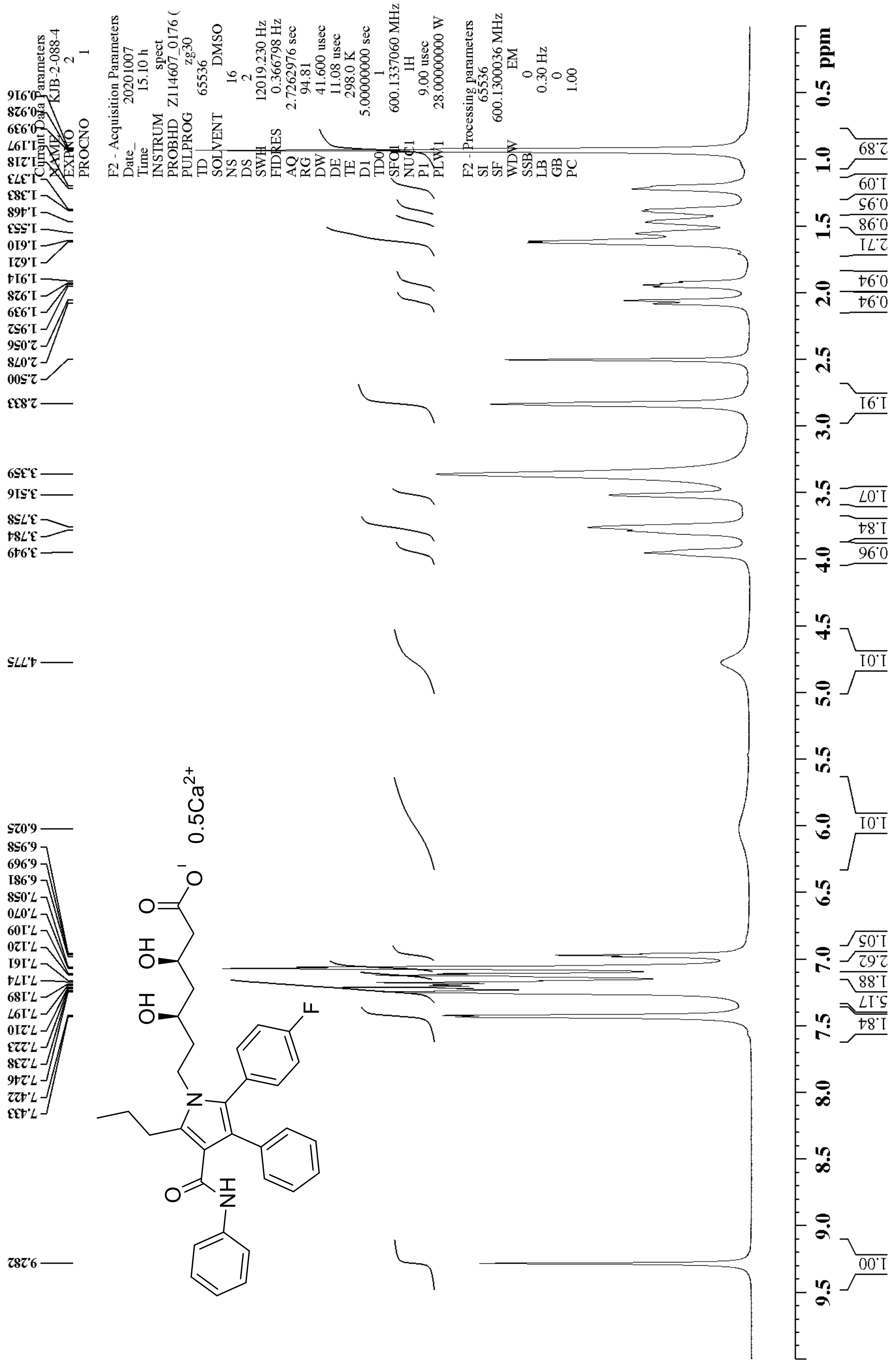

Supplementary Figure 47. ^{1}H NMR of (β*R*,δ*R*)-2-(4-fluorophenyl)-β,δ-dihydroxy-3-phenyl-4-[(phenylamino)carbonyl-5-propyl]-1*H*-pyrrole-1-heptanoic acid hemicalcium salt (3).

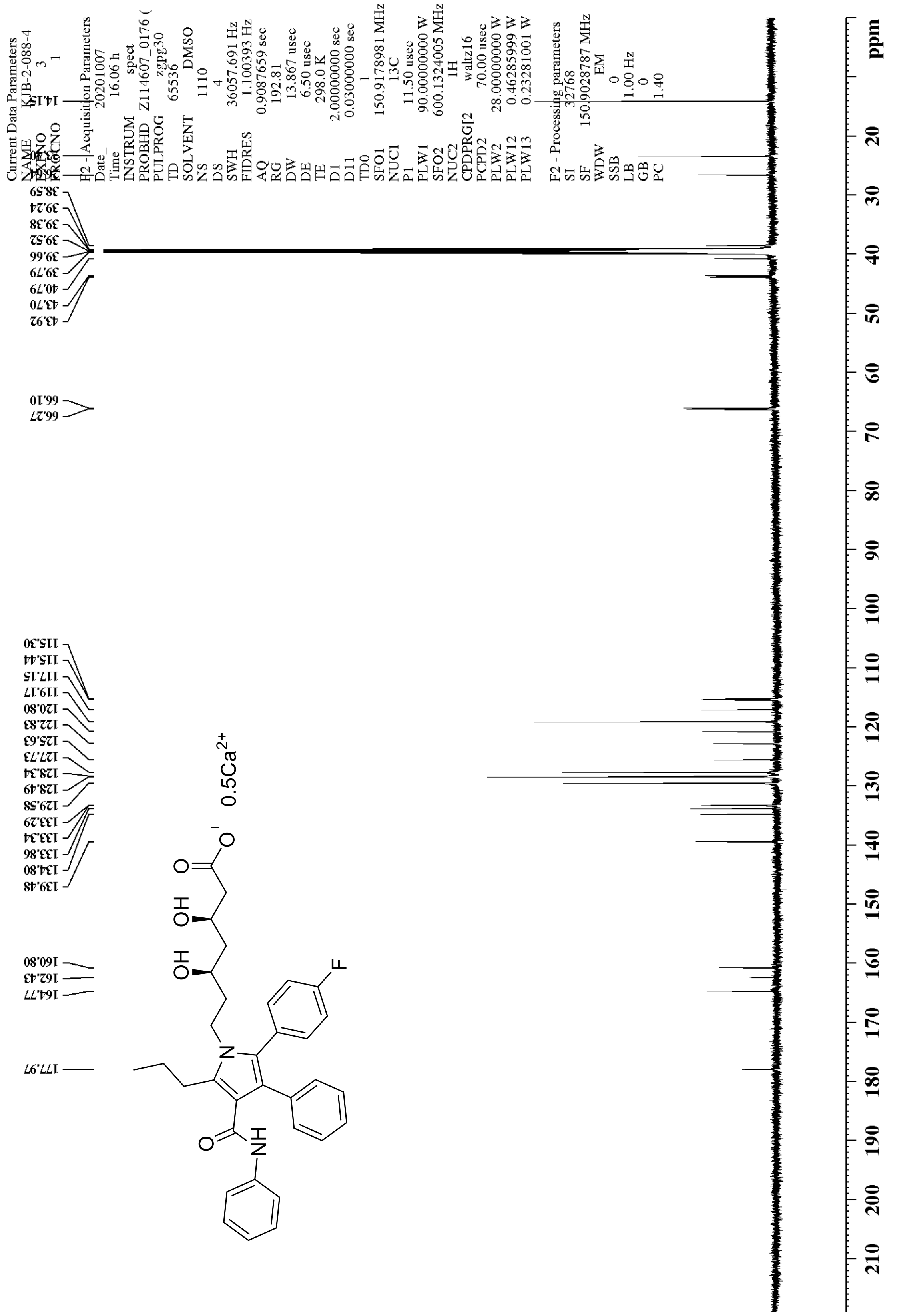

Supplementary Figure 48. ^{13}C NMR of (β*R*,δ*R*)-2-(4-fluorophenyl)-β,δ-dihydroxy-3-phenyl-4-[(phenylamino)carbonyl-5-propyl]-1*H*-pyrrole-1-heptanoic acid hemicalcium salt (3).

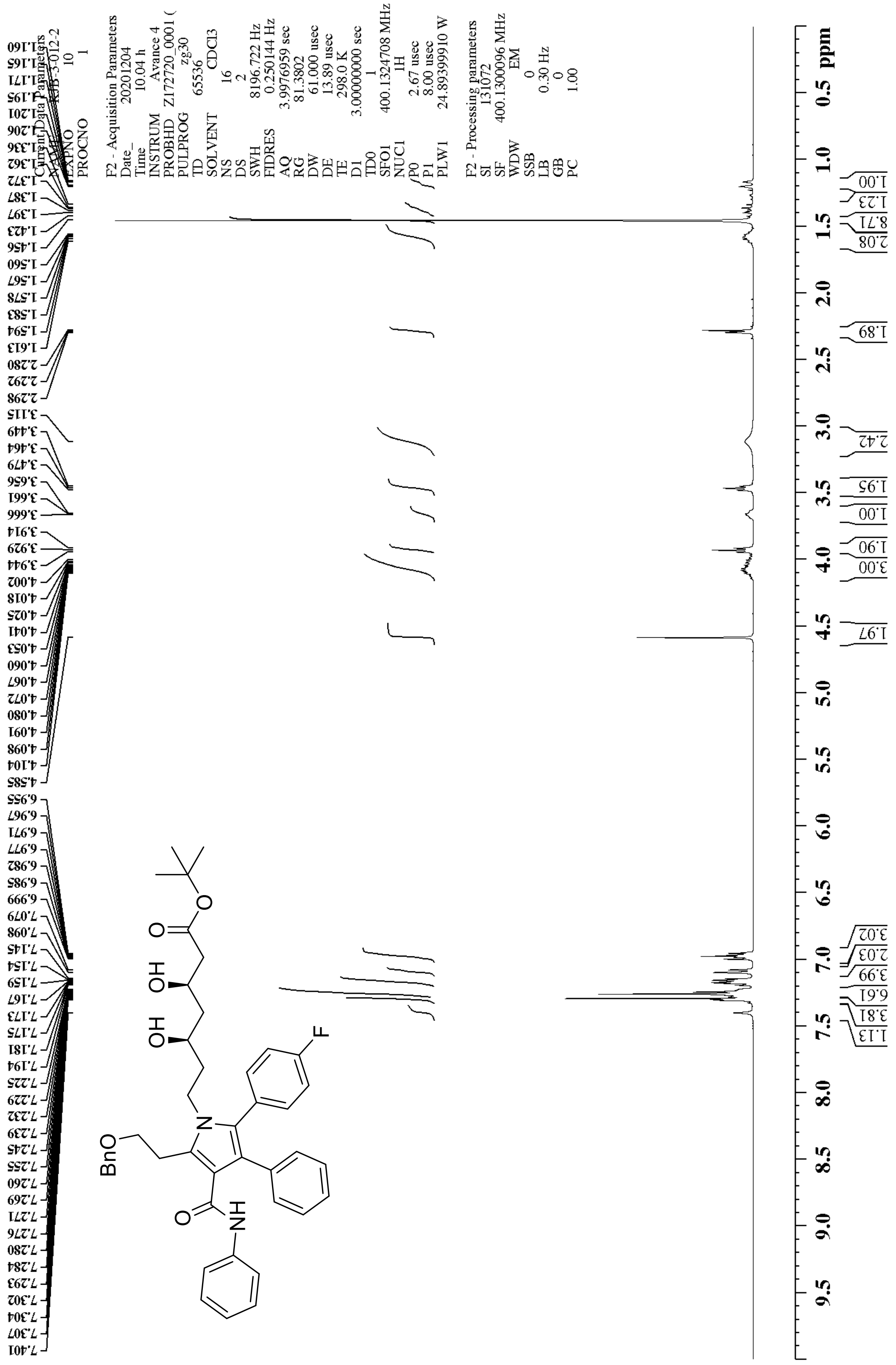

Supplementary Figure 49. ^{1}H NMR of 1,1-dimethylethyl (3*R*,5*R*)-7-[5-(2-benzyloxyethyl)-2-(4-fluorophenyl)-3-phenyl-4-phenylcarbamoylpyrrol-1-yl]-3,5-dihydroxyheptanoate.

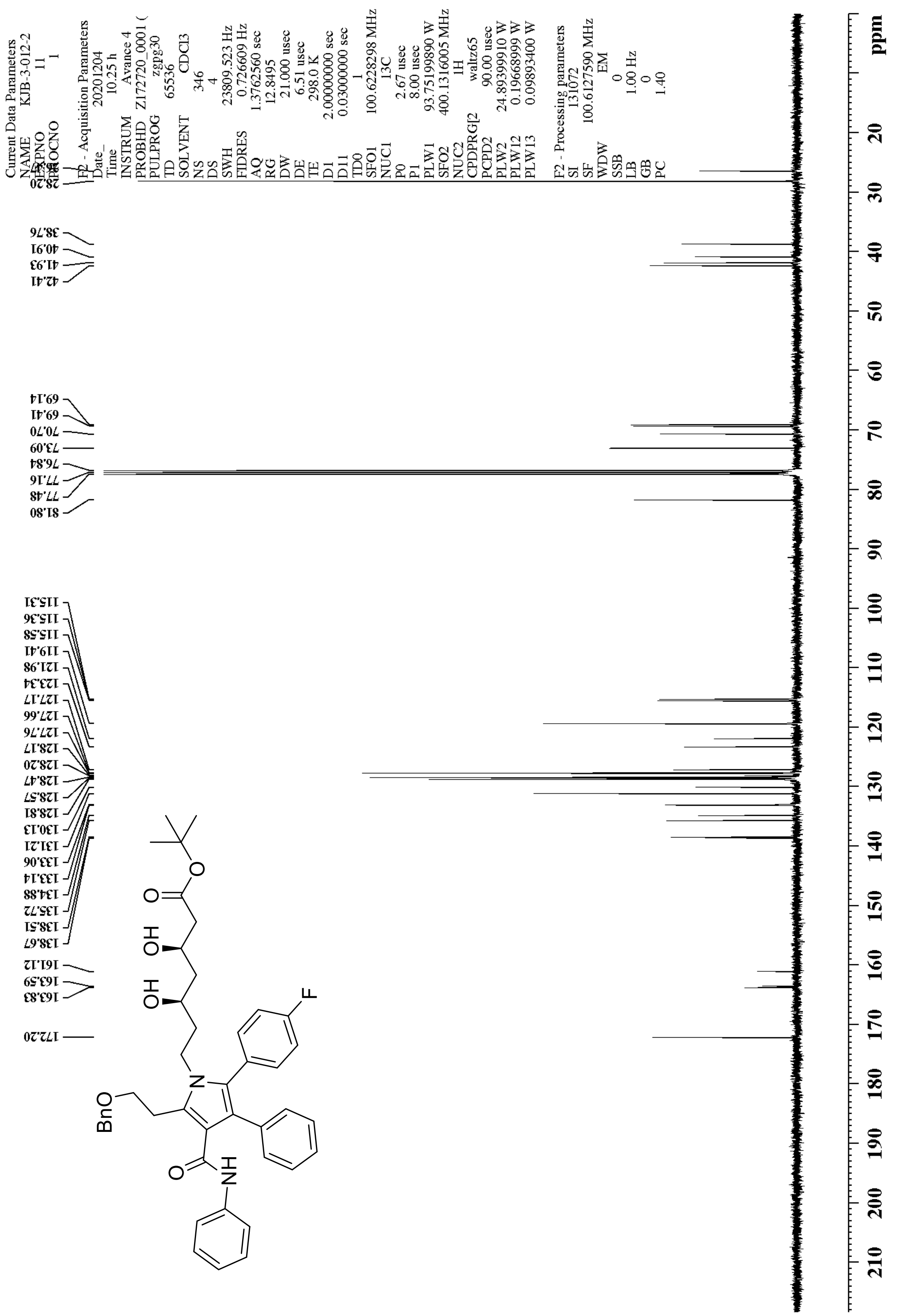

Supplementary Figure 50. ^{13}**C NMR of 1,1-dimethylethyl (3*R*,5*R*)-7-[5-(2-benzyloxyethyl)-2-(4-fluorophenyl)-3-phenyl-4-phenylcarbamoylpyrrol-1-yl]-3,5-dihydroxyheptanoate.**

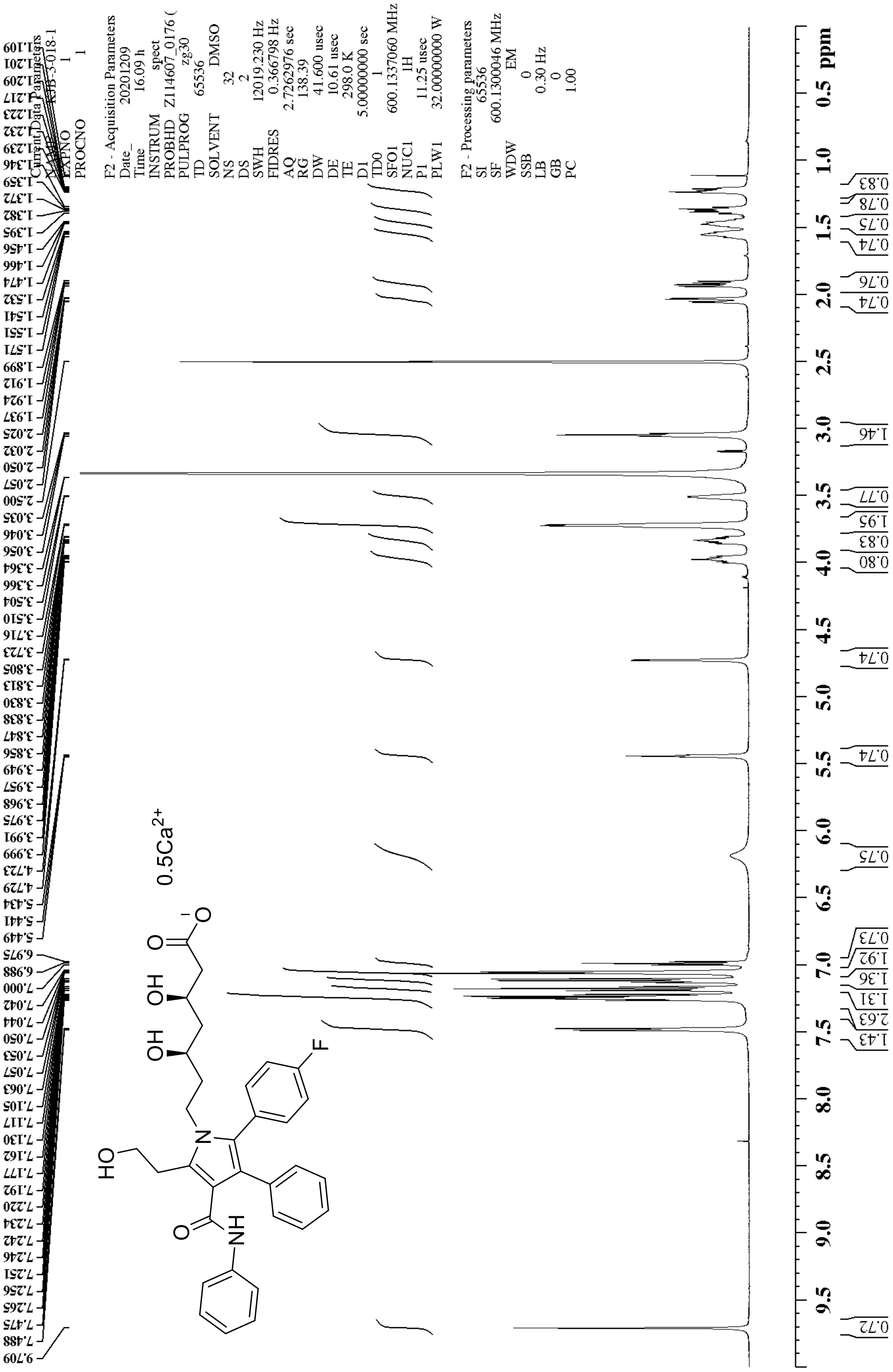

Supplementary Figure 51. ^{1}**H NMR of (β*R*,δ*R*)-2-(4-fluorophenyl)-β,δ-dihydroxy-5-(2-hydroxyethyl)-3-phenyl-4-[(phenylamino)carbonyl]-1*H*-pyrrole-1-heptanoic acid hemicalcium salt (4).**

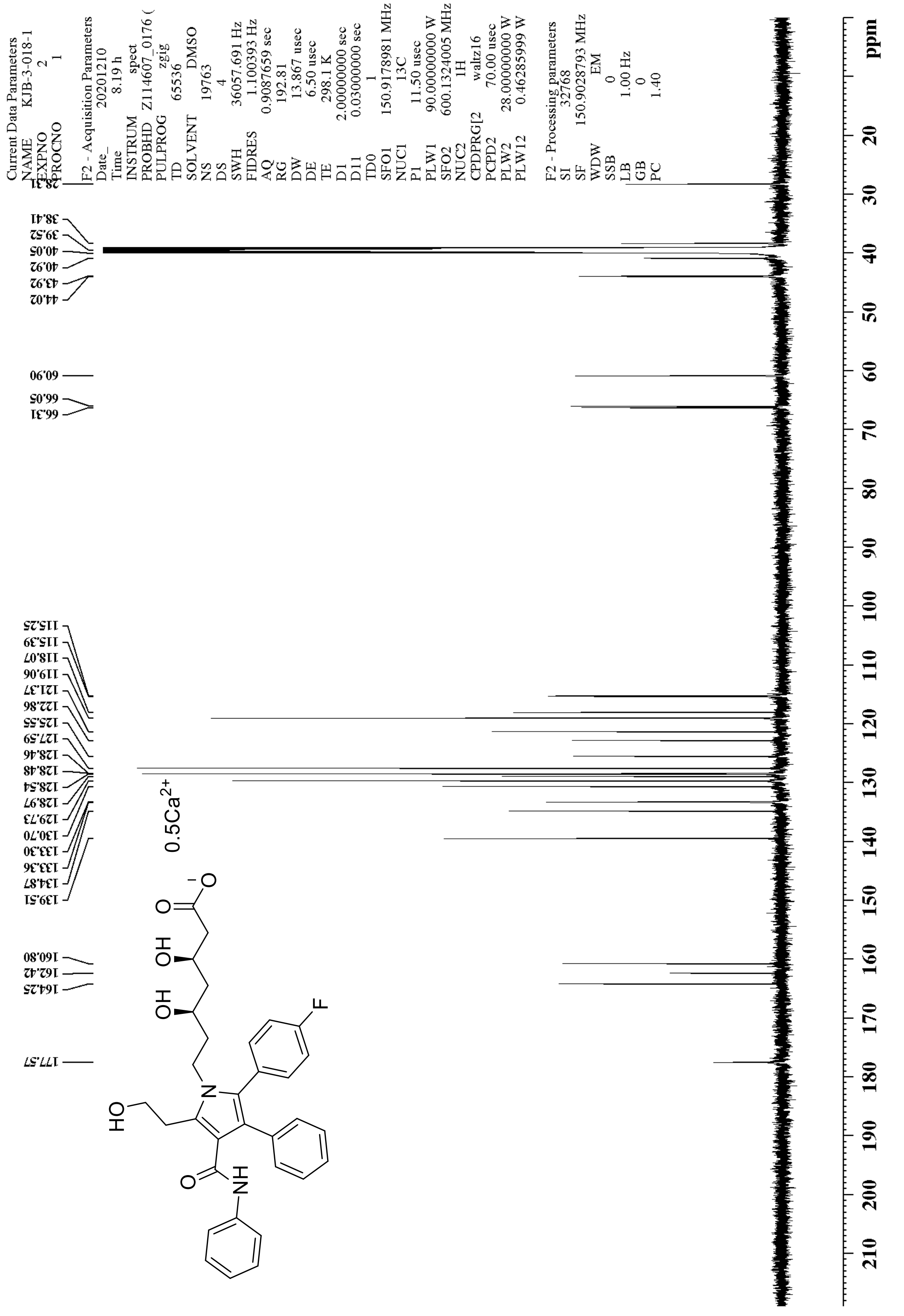

Supplementary Figure 52. ^{13}**C NMR of (β*R*,δ*R*)-2-(4-fluorophenyl)-β,δ-dihydroxy-5-(2-hydroxyethyl)-3-phenyl-4-[(phenylamino)carbonyl]-1*H*-pyrrole-1-heptanoic acid hemicalcium salt (4).**

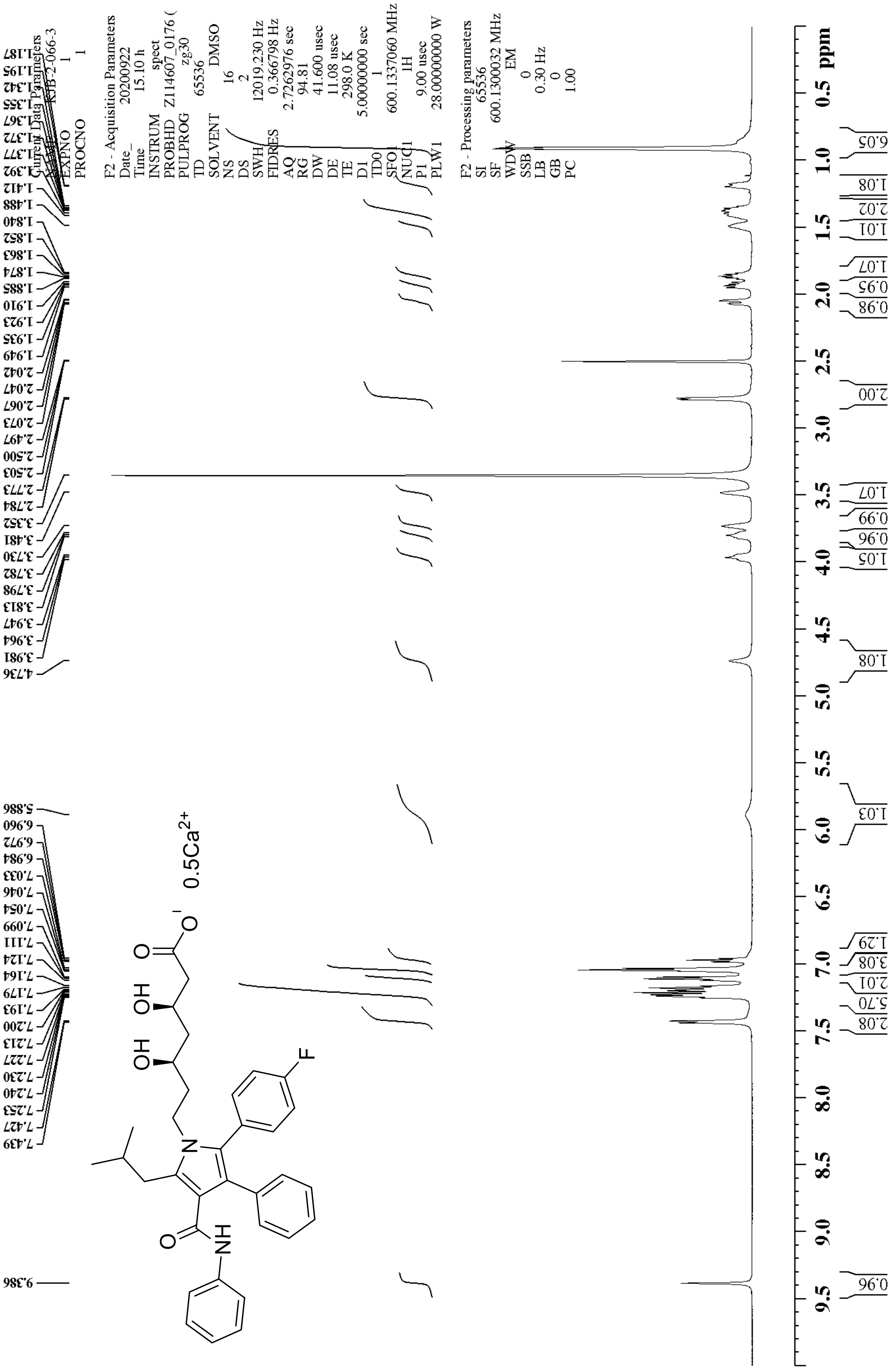

Supplementary Figure 53. **^{1}H NMR of (β*R*,δ*R*)-2-(4-fluorophenyl)-β,δ-dihydroxy-5-(2-methylpropyl)-3-phenyl-4-[(phenylamino)carbonyl]-1*H*-pyrrole-1-heptanoic acid hemicalcium salt (5).**

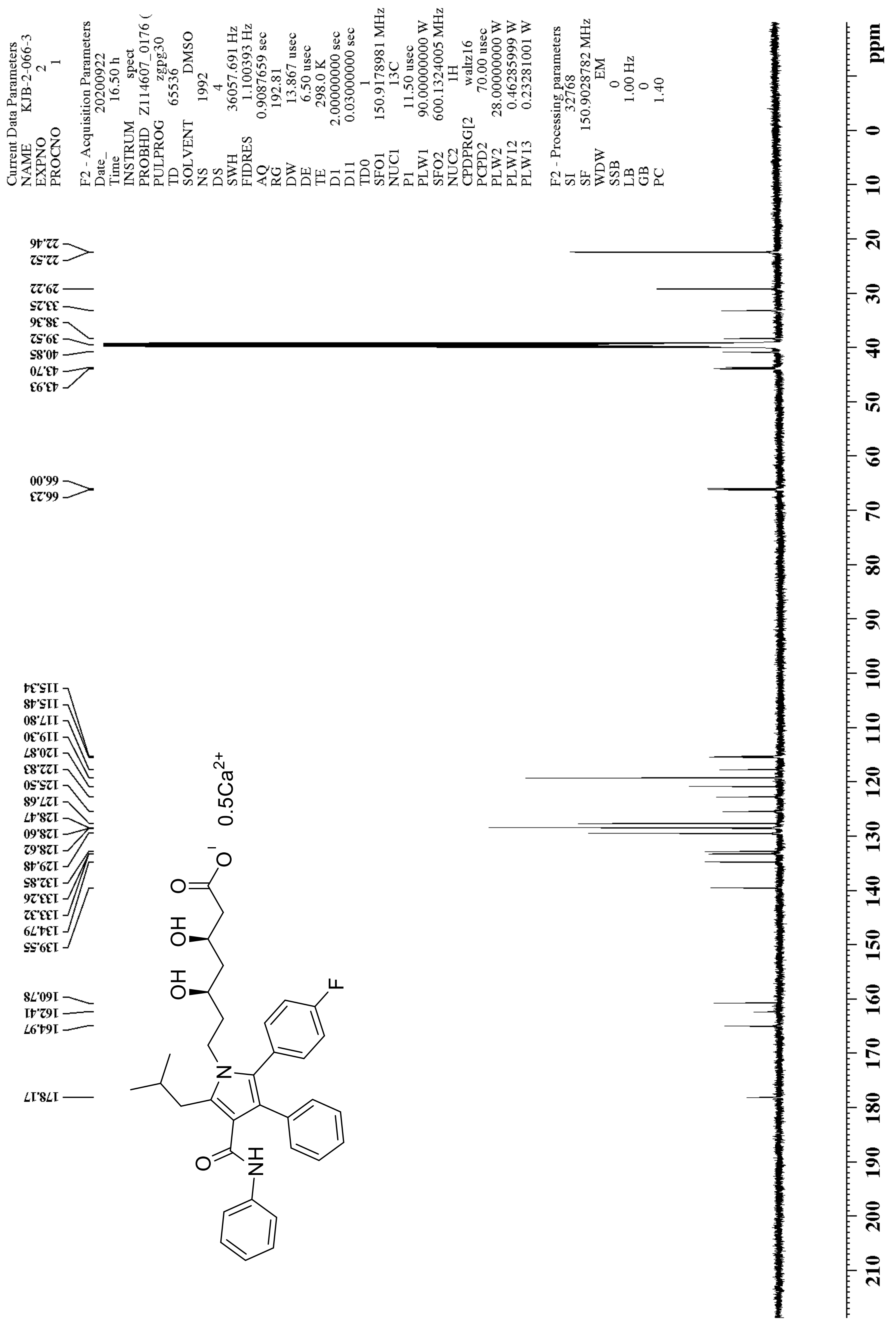

Supplementary Figure 54. ^{13}**C NMR of (β*R*,δ*R*)-2-(4-fluorophenyl)-β,δ-dihydroxy-5-(2-methylpropyl)-3-phenyl-4-[(phenylamino)carbonyl]-1*H*-pyrrole-1-heptanoic acid hemicalcium salt (5).**

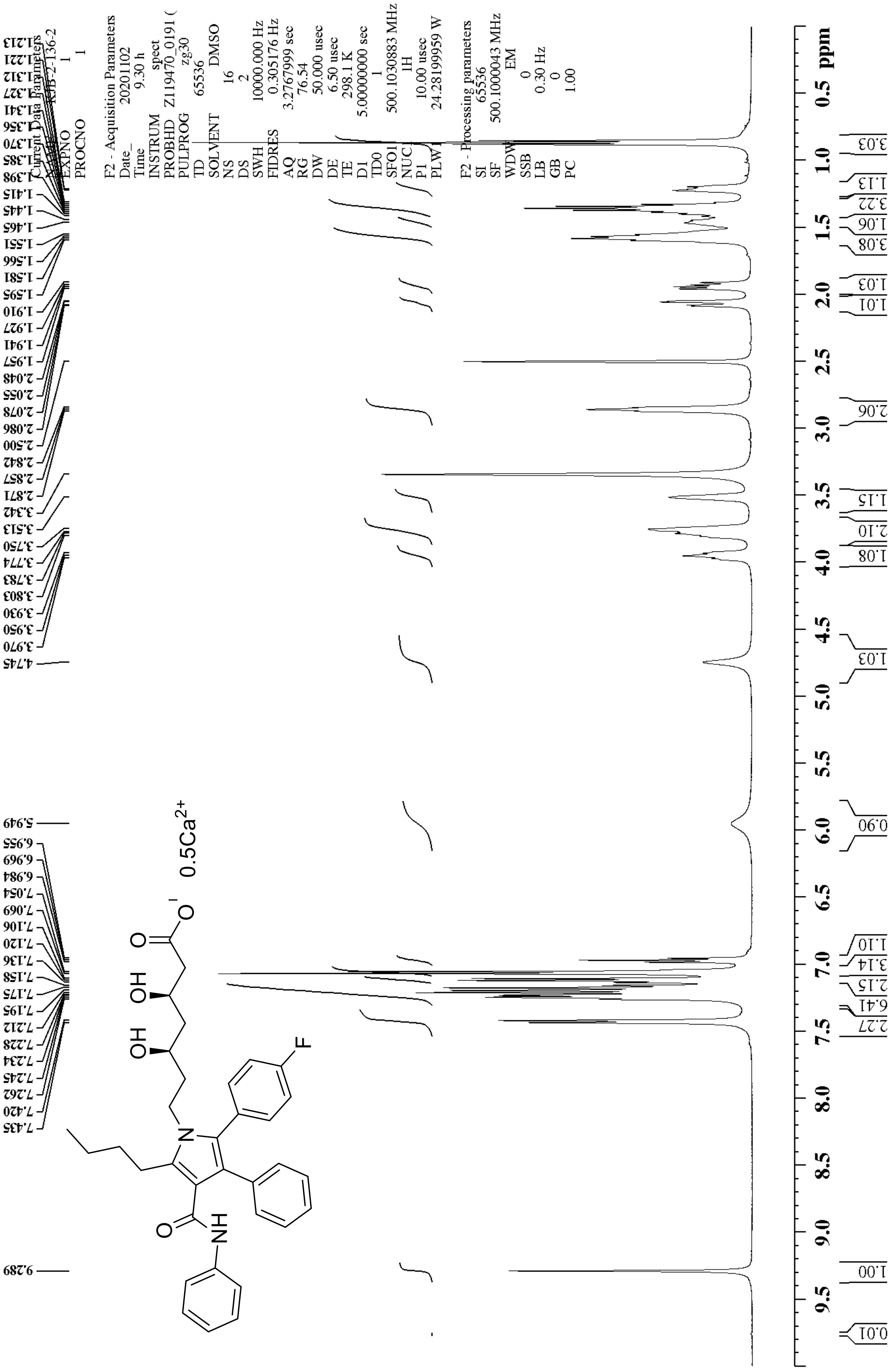

Supplementary Figure 55. ^{1}**H NMR of (β*R*,δ*R*)-5-butyl-2-(4-fluorophenyl)-β,δ-dihydroxy-3-phenyl-4-[(phenylamino)carbonyl]-1*H*-pyrrole-1-heptanoic acid hemicalcium salt (6).**

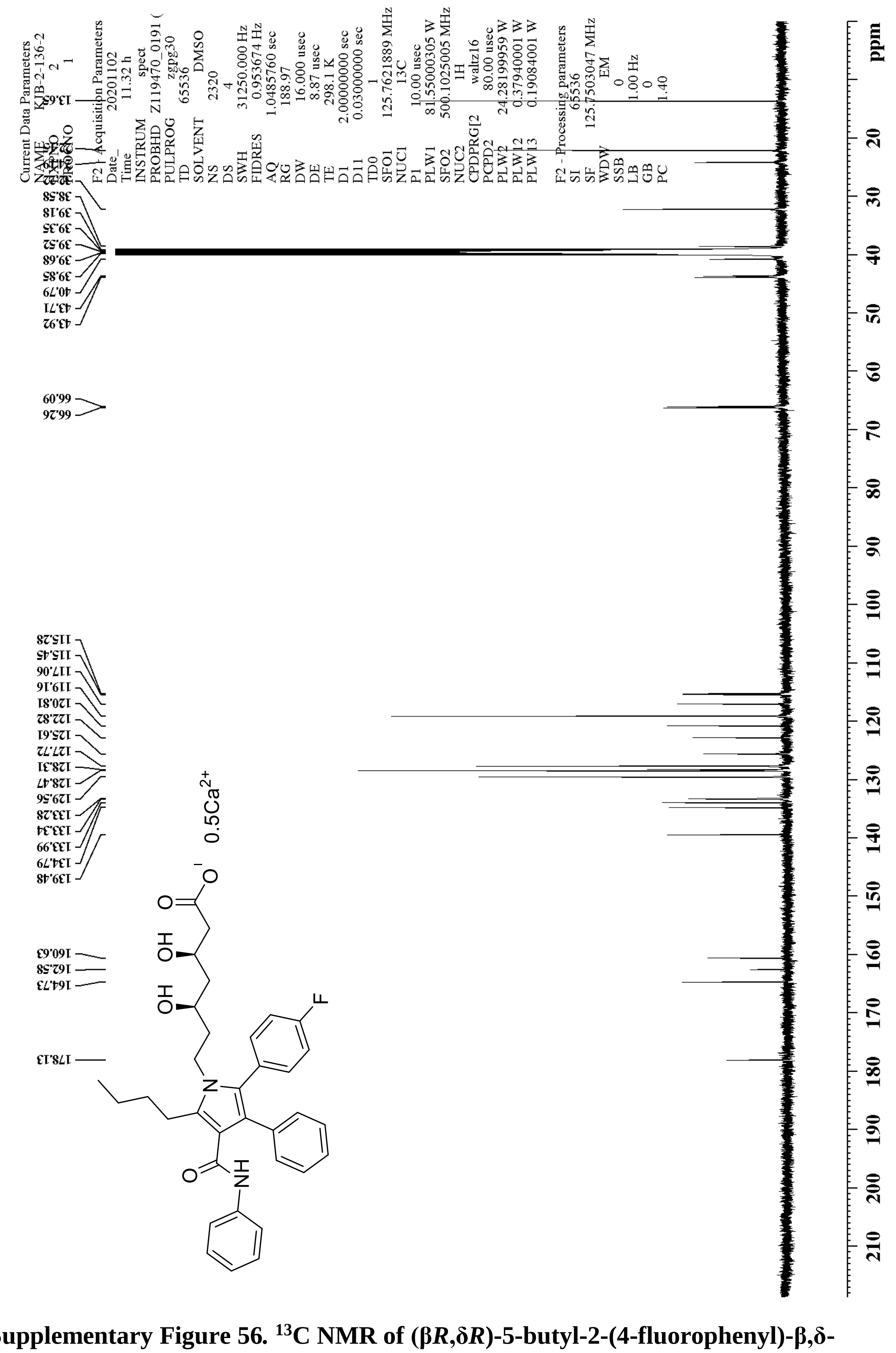

Supplementary Figure 56. ^{13}C NMR of (β*R*,δ*R*)-5-butyl-2-(4-fluorophenyl)-β,δ-dihydroxy-3-phenyl-4-[(phenylamino)carbonyl]-1*H*-pyrrole-1-heptanoic acid hemicalcium salt (6).

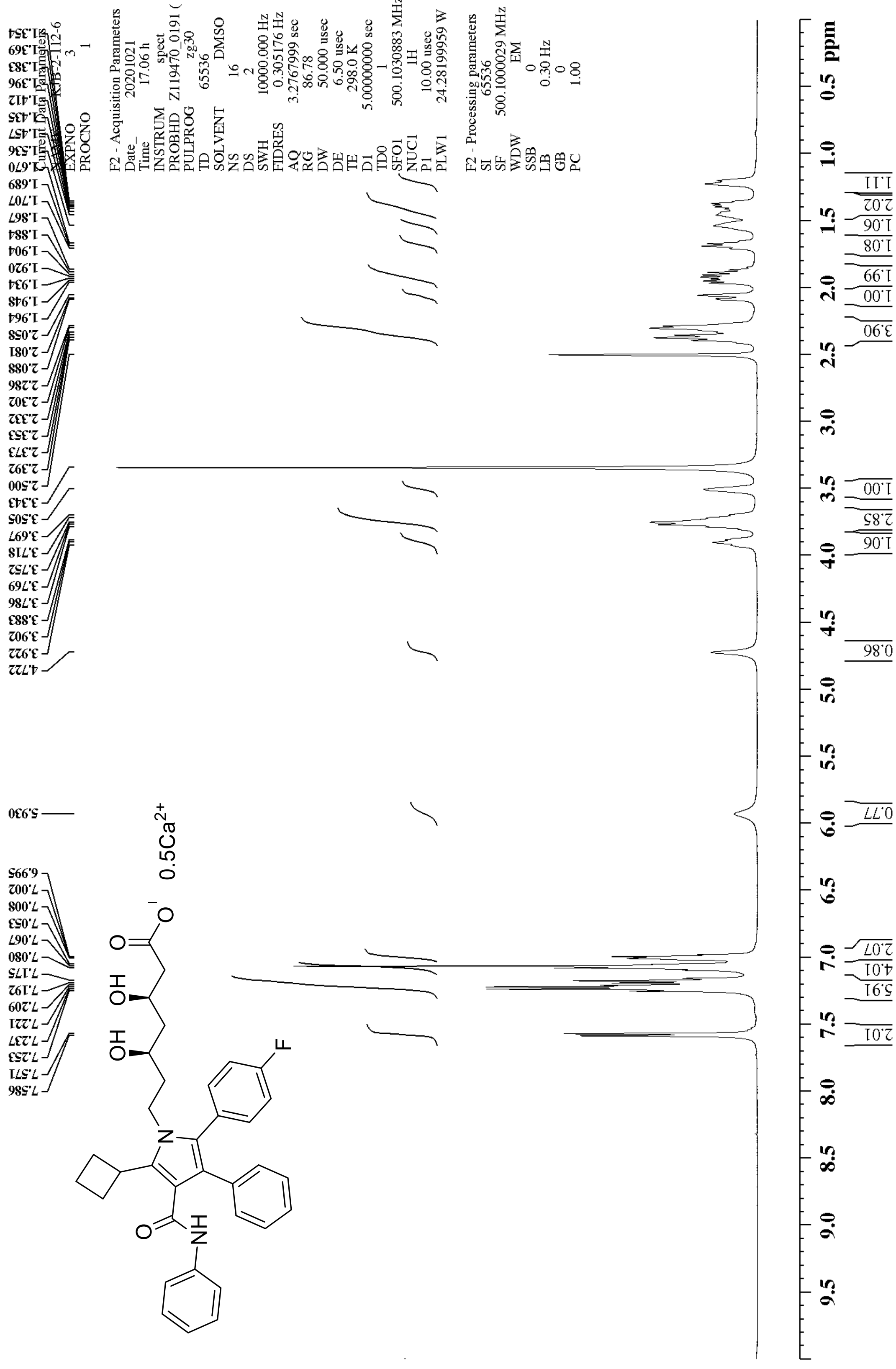

Supplementary Figure 57. ^{1}H NMR of (β*R*,δ*R*)-5-cyclobutyl-2-(4-fluorophenyl)-β,δ-dihydroxy-5-cyclobutyl-3-phenyl-4-[(phenylamino)carbonyl]-1*H*-pyrrole-1-heptanoic acid hemicalcium salt (7).

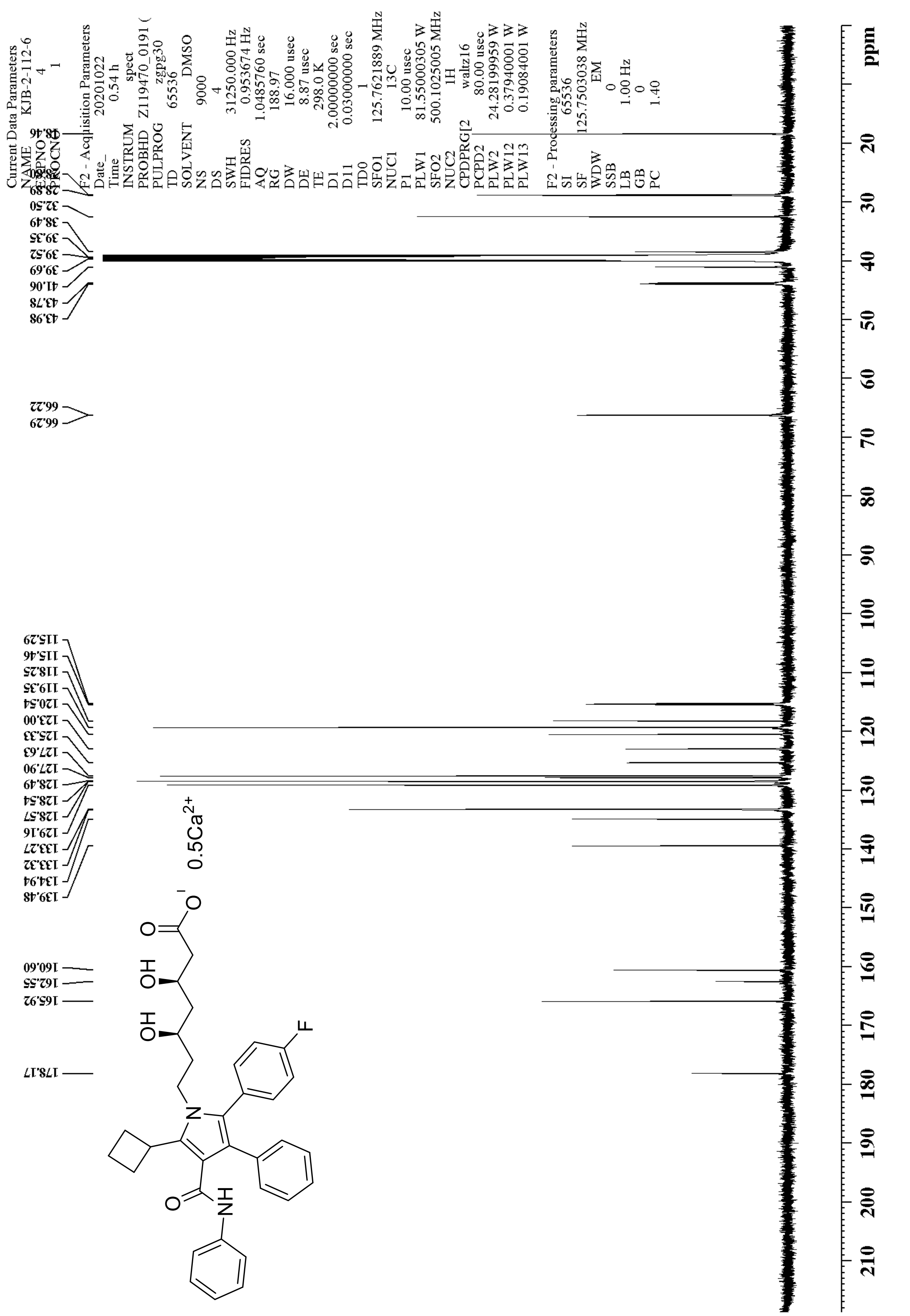

Supplementary Figure 58. ^{13}**C NMR of (β*R*,δ*R*)-5-cyclobutyl-2-(4-fluorophenyl)-β,δ-dihydroxy-5-cyclobutyl-3-phenyl-4-[(phenylamino)carbonyl]-1*H*-pyrrole-1-heptanoic acid hemicalcium salt (7).**

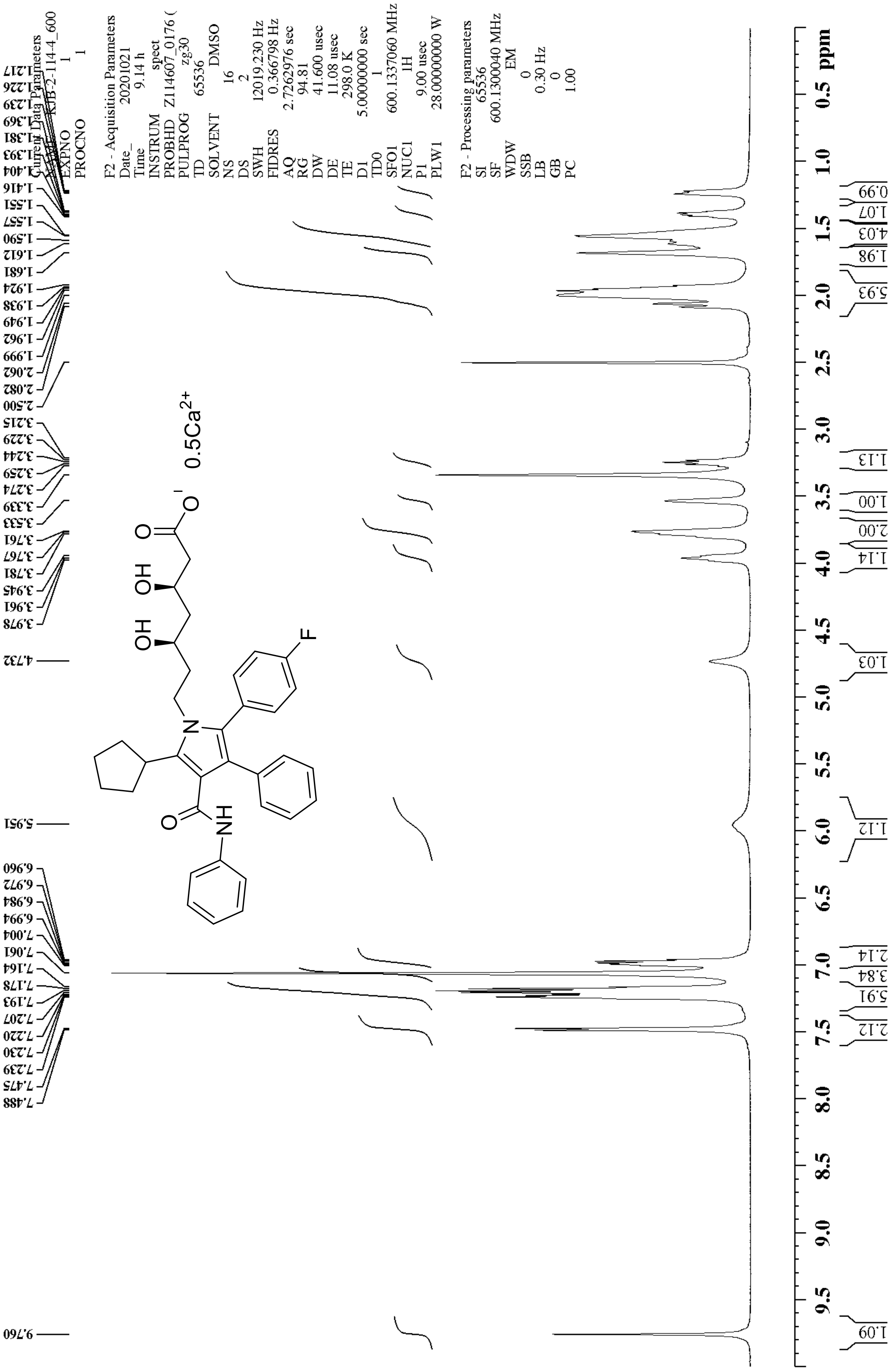

Supplementary Figure 59. ^{1}H NMR of (β*R*,δ*R*)-5-cyclopentyl-2-(4-fluorophenyl)-β,δ-dihydroxy-3-phenyl-4-[(phenylamino)carbonyl]-1*H*-pyrrole-1-heptanoic acid hemicalcium salt (8).

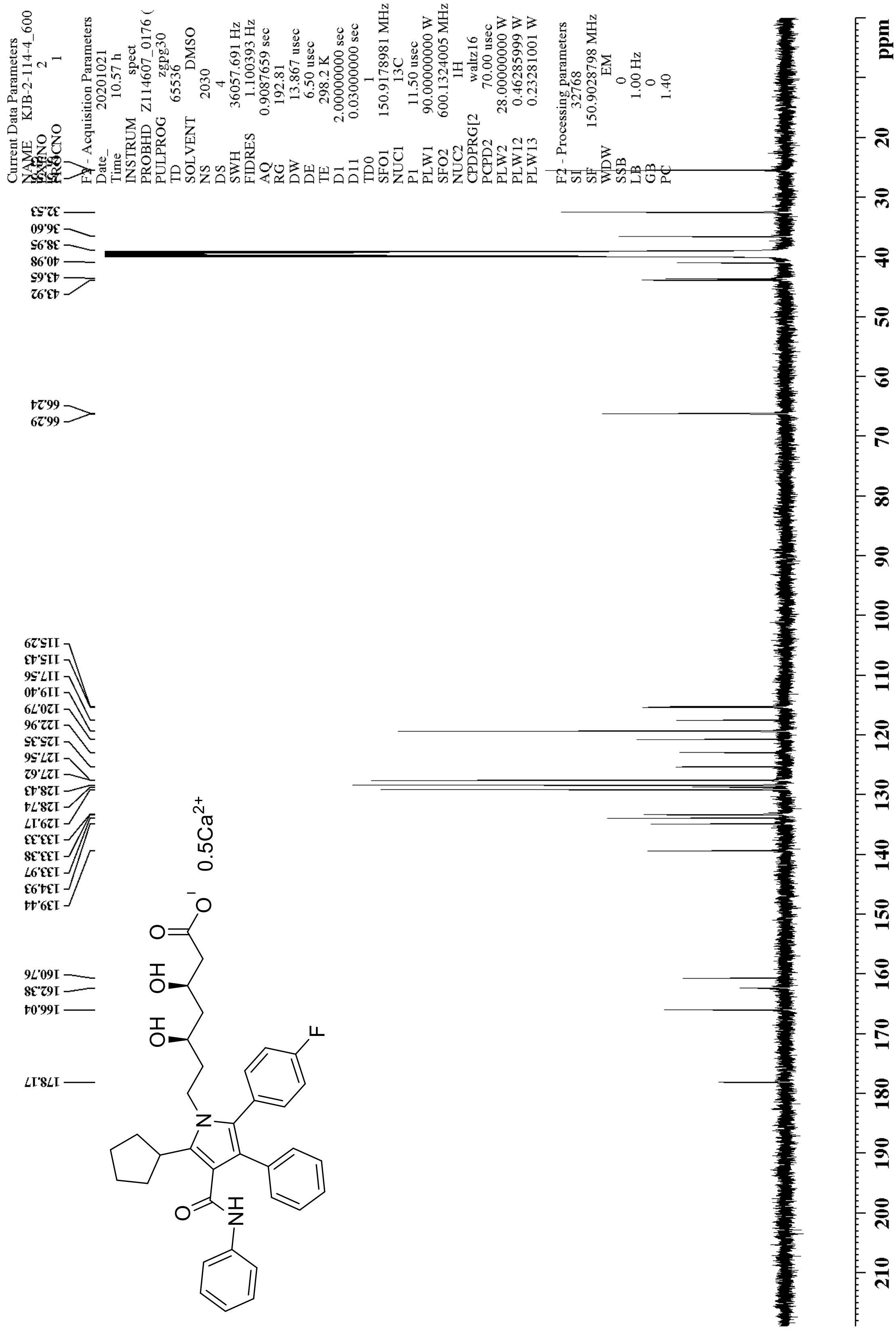

Supplementary Figure 60. ^{13}**C NMR of (β*R*,δ*R*)-5-cyclopentyl-2-(4-fluorophenyl)-β,δ-dihydroxy-3-phenyl-4-[(phenylamino)carbonyl]-1*H*-pyrrole-1-heptanoic acid hemicalcium salt (8).**

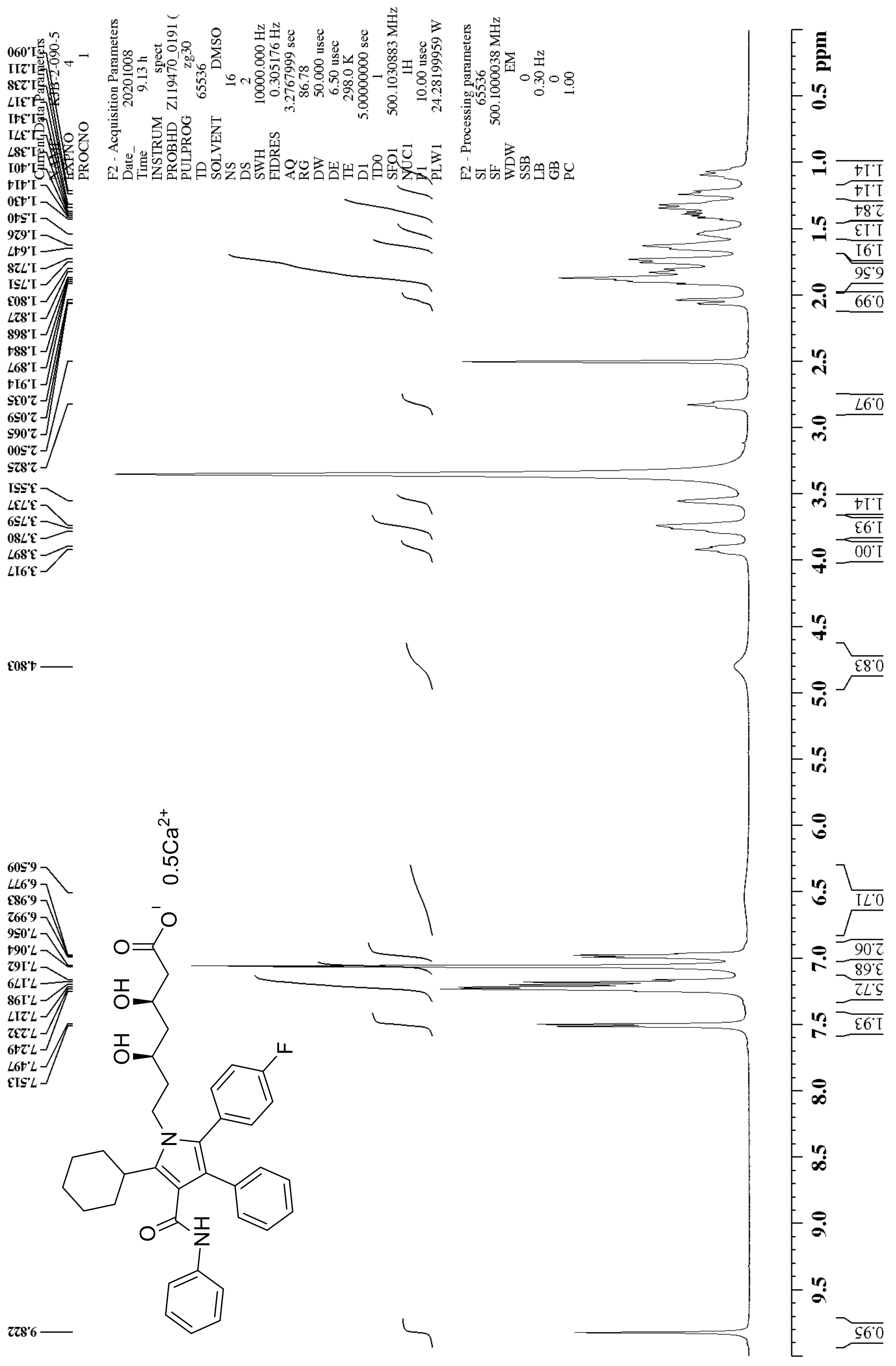

Supplementary Figure 61. **^{1}H NMR of (β*R*,δ*R*)-5-cyclohexyl-2-(4-fluorophenyl)-β,δ-dihydroxy-3-phenyl-4-[(phenylamino)carbonyl]-1*H*-pyrrole-1-heptanoic acid hemicalcium salt (9).**

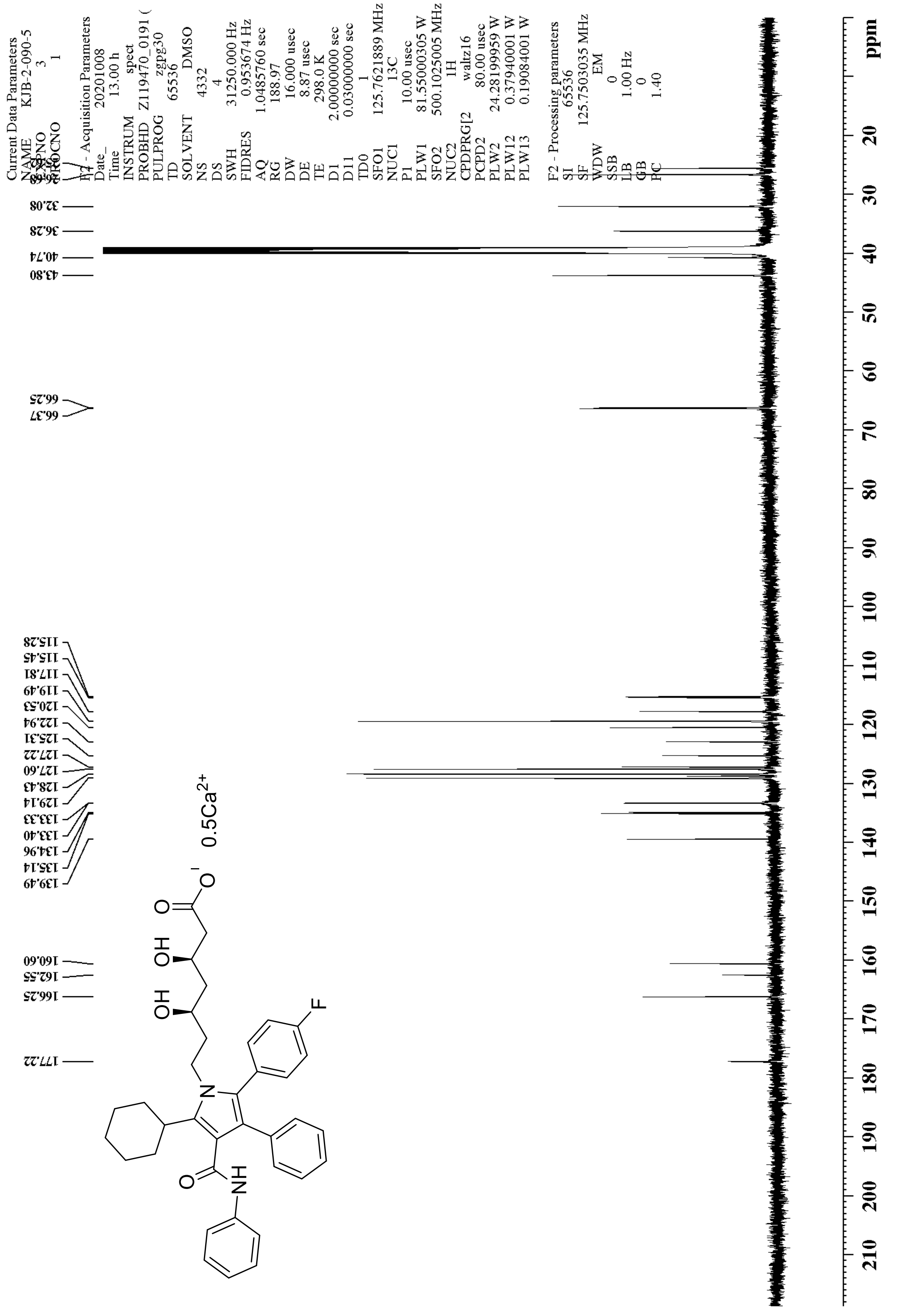

Supplementary Figure 62. **^{13}C NMR of (β*R*,δ*R*)-5-cyclohexyl-2-(4-fluorophenyl)-β,δ-dihydroxy-3-phenyl-4-[(phenylamino)carbonyl]-1*H*-pyrrole-1-heptanoic acid hemicalcium salt (9).**

Supplementary references

1. Kawade, R.K., Tseng, C.C. & Liu, R.S. Copper-catalyzed aerobic oxidations of 3-*N*-hydoxyaminoprop-1-ynes to form 3-substituted 3-amino-2-en-1-ones: oxidative Mannich reactions with a skeletal rearrangement. *Chemistry* **20**, 13927-31 (2014).
2. Boyle, R.G.e.a. CHK-1 Inhibitors. *PCT Int. Appl.* WO 2005028474 A2 (2005).
3. Yuan, Y. *et al*. One-pot synthesis of 3-hydroxyquinolin-2(1H)-ones from N-phenylacetoacetamide via PhI(OCOCF3)2-mediated α-hydroxylation and H2SO4-promoted intramolecular cyclization. *J Org Chem* **78**, 5385-92 (2013).
4. Xing, Y. *et al*. Efficient synthesis of the nucleus of atorvastatin calcium. *Synthetic Communications* **45**, 2832-2840 (2015).
5. Naidu, A.A. Synthesis of novel impurities in 2-(2-(4-fluorophenyl)-2-oxo-1-phenylethyl)-4-methyl-3-oxo-N-phenylpentanamide; an atorvastatin intermediate. *Organic Communications* **10**, 314 (2017).
6. Sattigeri, J.A., Sethi, S. & Kishore, K. Process for preparation of (3R, 5R)-7-[2-(4-fluorophenyl)-5-isopropyl-3-phenyl-4-[(4-hydroxy methyl phenyl amino) carbonyl]-pyrrol-1-yl]-3, 5-dihydroxy-heptanoic acid hemi calcium salt. (Google Patents, 2010).
7. Estévez, V., Villacampa, M. & Menéndez, J.C. Concise synthesis of atorvastatin lactone under high-speed vibration milling conditions. *Organic Chemistry Frontiers* **1**, 458-463 (2014).
8. Sukhoverkov, K.V. *et al.* Improved herbicide discovery using physico-chemical rules refined by antimalarial library screening. *RSC Advances* **11**, 8459-8467 (2021).
9. Istvan, E.S., Palnitkar, M., Buchanan, S.K. & Deisenhofer, J. Crystal structure of the catalytic portion of human HMG-CoA reductase: insights into regulation of activity and catalysis. *EMBO Journal* **19**, 819-830 (2000).
10. Robert, X. & Gouet, P. Deciphering key features in protein structures with the new ENDscript server. *Nucleic Acids Research* **42**, W320-W324 (2014).
11. Crooks, G.E., Hon, G., Chandonia, J.-M. & Brenner, S.E. WebLogo: A sequence logo generator. *Genome Research* **14**, 1188-1190 (2004).
12. Kennedy, J. *et al.* Modulation of polyketide synthase activity by accessory proteins during lovastatin biosynthesis. *Science* **284**, 1368-72 (1999).
13. Hutchinson, C.R. *et al.* Aspects of the biosynthesis of non-aromatic fungal polyketides by iterative polyketide synthases. *Antonie Van Leeuwenhoek* **78**, 287-95 (2000).
14. Martín, J.-F., García-Estrada, C. & Zeilinger, S. *Biosynthesis and Molecular Genetics of Fungal Secondary Metabolites*, (Springer, 2014).
15. McNutt, A.T. *et al.* GNINA 1.0: molecular docking with deep learning. *Journal of Cheminformatics* **13**, 1-20 (2021).
