# Supplementary material for: A fungal tolerance trait and selective inhibitors proffer HMG-CoA reductase as a herbicide mode-of-action
Source: Nat Commun. 2022 Sep 22;13:5563. doi: 10.1038/s41467-022-33185-0 (PMC9500038; doi:10.1038/s41467-022-33185-0)
Supplement: Supplementary file 2 — Reporting Summary [file 41467_2022_33185_MOESM2_ESM.pdf]

nature portfolio

Corresponding author(s): Joel Haywood & Joshua S. Mylne

Last updated by author(s): Aug 19, 2022

Reporting Summary

Nature Portfolio wishes to improve the reproducibility of the work that we publish. This form provides structure for consistency and transparency in reporting. For further information on Nature Portfolio policies, see our Editorial Policies and the Editorial Policy Checklist.

Statistics

For all statistical analyses, confirm that the following items are present in the figure legend, table legend, main text, or Methods section.

n/a	Confirmed	
☐	☒	The exact sample size (*n*) for each experimental group/condition, given as a discrete number and unit of measurement
☐	☒	A statement on whether measurements were taken from distinct samples or whether the same sample was measured repeatedly
☐	☒	The statistical test(s) used AND whether they are one- or two-sided<br>*Only common tests should be described solely by name; describe more complex techniques in the Methods section.*
☒	☐	A description of all covariates tested
☒	☐	A description of any assumptions or corrections, such as tests of normality and adjustment for multiple comparisons
☐	☒	A full description of the statistical parameters including central tendency (e.g. means) or other basic estimates (e.g. regression coefficient) AND variation (e.g. standard deviation) or associated estimates of uncertainty (e.g. confidence intervals)
☐	☒	For null hypothesis testing, the test statistic (e.g. *F*, *t*, *r*) with confidence intervals, effect sizes, degrees of freedom and *P* value noted<br>*Give P values as exact values whenever suitable.*
☒	☐	For Bayesian analysis, information on the choice of priors and Markov chain Monte Carlo settings
☒	☐	For hierarchical and complex designs, identification of the appropriate level for tests and full reporting of outcomes
☒	☐	Estimates of effect sizes (e.g. Cohen's *d*, Pearson's *r*), indicating how they were calculated

Our web collection on statistics for biologists contains articles on many of the points above.

Software and code

Policy information about availability of computer code

Data collection	*Provide a description of all commercial, open source and custom code used to collect the data in this study, specifying the version used OR state that no software was used.*
Data analysis	To analyse the data in this study the following software was used were: XDS (Version: Jan 10, 2022), CCP4 program suite (v8), Clustal O (v1.2.4), Coot (v0.9.8.1), PyMol (v2.5), ImageJ (v1.53), GraphPad Prism (v9), WebLogo (v2.8.2), Gnina (v1.0.1)

For manuscripts utilizing custom algorithms or software that are central to the research but not yet described in published literature, software must be made available to editors and reviewers. We strongly encourage code deposition in a community repository (e.g. GitHub). See the Nature Portfolio guidelines for submitting code & software for further information.

Data

Policy information about availability of data

All manuscripts must include a data availability statement. This statement should provide the following information, where applicable:

- Accession codes, unique identifiers, or web links for publicly available datasets
- A description of any restrictions on data availability
- For clinical datasets or third party data, please ensure that the statement adheres to our policy

Provide your data availability statement here.

Human research participants

Policy information about studies involving human research participants and Sex and Gender in Research.

Reporting on sex and gender	This study does not include any gender- or sex-based analyses
Population characteristics	See above
Recruitment	N/A
Ethics oversight	N/A

Note that full information on the approval of the study protocol must also be provided in the manuscript.

Field-specific reporting

Please select the one below that is the best fit for your research. If you are not sure, read the appropriate sections before making your selection.

☒ Life sciences ☐ Behavioural & social sciences ☐ Ecological, evolutionary & environmental sciences

For a reference copy of the document with all sections, see nature.com/documents/nr-reporting-summary-flat.pdf

Life sciences study design

All studies must disclose on these points even when the disclosure is negative.

Sample size	Fig 1. Herbicidal activity of statins between a model dicot and a monocot, n=3. Sample size was constrained by balancing the cost of performing the experiment with statistical power. n=3 was chosen as minimum required to demonstrate variation as the experiment. No statistical methods were used to predetermine sample size.<br>Fig.4. In vitro species selectivity assays, n=3 independent reactions. Sample size was constrained by balancing the cost of performing the experiment with statistical power. n=3 was chosen as minimum required to demonstrate variation as the experiment. No statistical methods were used to predetermine sample size.<br>Fig.5. In vitro resistance assays, n=3 independent reactions. Sample size was constrained by balancing the cost of performing the experiment with statistical power. n=3 was chosen as minimum required to demonstrate variation as the experiment. No statistical methods were used to predetermine sample size.<br>Fig.6. In planta resistance assay, n=19 transgenic lines 35S::AtHMG1 and n=14 transgenic lines 35S::AtHMG1-L558T. Sample size chosen using all available lines with similar levels of hygromycin resistance.
Data exclusions	Fig.4. In vitro species selectivity assays, n=3 independent reactions except for a single point (AtHMG1 333 µM At. n = 2). Point excluded due to pipetting error - bubbles in well
Replication	Fig 1. Herbicidal activity of statins between a model dicot and a monocot, n=3. Performed once.<br>Fig.4. In vitro species selectivity assays, b, n=3 independent reactions. Repeated in dose range experiments, d, shows trends reproducible.<br>Fig.5. In vitro resistance assays, n=3 independent reactions. Repeated in dose range experiment, c, and in planta (Fig 6) shows resistance reproducible.<br>Fig.6. In planta resistance assay, n=19 transgenic lines 35S::AtHMG1 and n=14 transgenic lines 35S::AtHMG1-L558T. Performed once.
Randomization	Selection of plants for treatments was done randomly.<br>Fig 1. Approximately equal numbers of seeds from the same Col-0 or Tef seeds were used for sowing in pots and pots randomly selected for treatment.<br>Fig. 4a. Approximately equal numbers of seeds from the same Col-0 seeds were used for sowing in pots and pots randomly selected for treatment.<br>Fig. 6. WT col-0 A. thaliana or transformed seeds were distributed evenly on agar plates.
Blinding	Fig. 1. Experiments were performed by single individual and blinding not performed.<br>Fig. 4a. Experiments were performed by single individual and blinding not performed.<br>Fig. 4b-d. In vitro species selectivity assays were performed blinded from the structure of compounds 1-9<br>Fig. 5. Experiments were performed by single individual and blinding not performed.<br>Fig. 6. Experiments were performed by single individual and blinding not performed.

Reporting for specific materials, systems and methods

We require information from authors about some types of materials, experimental systems and methods used in many studies. Here, indicate whether each material, system or method listed is relevant to your study. If you are not sure if a list item applies to your research, read the appropriate section before selecting a response.

Materials & experimental systems

n/a	Involved in the study
☒	☐ Antibodies
☒	☐ Eukaryotic cell lines
☒	☐ Palaeontology and archaeology
☒	☐ Animals and other organisms
☒	☐ Clinical data
☒	☐ Dual use research of concern

Methods

n/a	Involved in the study
☒	☐ ChIP-seq
☒	☐ Flow cytometry
☒	☐ MRI-based neuroimaging
